# Research methods and efficacy of acupuncture in the treatment of Parkinson's disease: a scoping review of systematic reviews and meta-analyses

**DOI:** 10.3389/fneur.2023.1196446

**Published:** 2023-06-02

**Authors:** Bin Yu, Shi-qi Ma, Hai-peng Huang, Zhen Zhong, Shuo Yu, Ke Huang, Li-ying Zhang, Meng-yuan Li, Lin Yao

**Affiliations:** ^1^College of Traditional Chinese Medicine, Changchun University of Chinese Medicine, Changchun, China; ^2^College of Acupuncture and Massage, Changchun University of Chinese Medicine, Changchun, China; ^3^Institute of Acupuncture and Tuina, Northeast Asia Academy of Traditional Chinese Medicine, Changchun University of Chinese Medicine, Changchun, China; ^4^The First Affiliated Hospital of Guangzhou University of Chinese Medicine, Guangzhou, China

**Keywords:** acupuncture, Parkinson's disease, scoping review, systematic reviews, meta-analyses

## Abstract

**Introduction:**

Research on acupuncture for Parkinson's Disease is growing rapidly. A scoping review examines emerging evidence and is important to guide policy and practice. The purpose of this scoping review was to examine the breadth and methodological quality of systematic reviews and meta-analyses, and to map the quality of evidence of these studies to evaluate the efficacy of acupuncture for treatment of PD.

**Methods:**

Seven literature databases were searched. Two researchers independently screened the literature and extracted relevant information (such as general characteristics, inclusion criteria, study results, and report quality).The inclusion criteria include publicly published systematic reviews/meta-analyses/systematic reviews of acupuncture treatment for Parkinson's disease. The research subjects are any patients who meet the diagnostic criteria for Parkinson's disease, and intervention measures include acupuncture treatment including electro acupuncture, scalp acupuncture, or combination with other treatment methods. The outcome indicators are all types of results related to PD and the effective measurement tools used.

**Results:**

A total of 23 systematic reviews and/or meta-analyses of studies were included. Most of the articles were published between 2019 and 2023 (47.8%). A total of 14 articles (60.9%) were evaluated and classified, and 89 (36.8.1%) of the 242 included articles were of medium and high quality.

**Discussion:**

This study comprehensively evaluates the quality and research methods of incorporating SRs/MAs, and concludes that acupuncture treatment for Parkinson's disease may be significant. Considering the shortcomings in research design and methodology, it is not possible to draw conclusions on the evidence of acupuncture treatment for PD at this stage, but it does not mean that acupuncture treatment is ineffective. We hope to focus on improving research design and methods in the study of acupuncture treatment for Parkinson's disease, an increase the credibility of research results.

## Introduction

Parkinson's disease (PD) is a chronic neurological condition that worsens with age. The main symptoms include the development of motor and nonmotor symptoms, degeneration of dopaminergic neurons in the substantia nigra pars compact of the midbrain, lowering of dopamine levels in the striatum, accumulation of α-synuclein in the cytoplasm, and formation of Lewy bodies (1, 2). Bradykinesia, tremor, rigidity, freezing of gait, and unstable posture and gait are among the primary symptoms of the disease (3). Although the exact cause of PD is unknown, it is generally accepted that immunological, inflammatory, and mitochondrial abnormalities may play a role (4). Between 5/100,000 and 35/100,000 individuals are diagnosed with PD each year (5). Incidence increases 5- to 10-fold between the ages of 60 and 90 years (6), and also increases with age. It is estimated that the prevalence of PD will double over the next 20 years as the world population ages (7). Without the development of more potent therapies, cures, or preventative measures, the social and financial burden of PD will only continue to increase (8).

The evidence for a cure for PD is still insufficient. Although levodopa and madopar, two traditional medications used to treat PD, have good efficacy, they can only cure symptoms, and long-term use can cause negative side effects such as nausea, vomiting, and arrhythmias. The incidence of patients with PD who use complementary and alternative medicine ranges from 25.7 to 76% according to epidemiological statistics from seven different countries (9, 10). As a result, an increasing number of patients with PD seek complementary and alternative medicine (11–13).

The most common adjuvant therapy for Parkinson's disease is acupuncture (14). Acupuncture has long been used clinically to treat PD, especially in China, and is a crucial part of traditional Chinese medicine. The efficacy of acupuncture has been demonstrated in clinical settings and has its roots in naïve philosophy and empirical medicine from antiquity (15). Acupuncture has been shown to be beneficial in the treatment of PD in numerous clinical investigations to date (16–18).

Scoping reviews are used to identify knowledge gaps, examine the relevant literature, clarify concepts, or study behavior (19). They emphasize benefits and limitations (i.e., methodological quality) of treatments, review the existing data, identify knowledge gaps, and determine the future research direction based on relatively new evidence, and thus represent a comprehensive evidence synthesis method. Future clinical trials can use these data to prioritize different diseases or treatments (20). Scoping reviews may be a suitable method for examining available information on acupuncture for the treatment of PD, which can serve as the basis for a systematic review.

The purpose of this scoping review was to examine the research methods and methodological quality of systematic reviews and meta-analyses, and to map the available evidence evaluating the efficacy of acupuncture for PD. The scoping review of this project will aid in the design of subsequent systematic reviews and will indicate areas for future research to address knowledge gaps.

## Methods

### Database and search strategy

The PRISMA-ScR checklist (21) was followed for conducting this scoping review, and the following databases were searched without regard to language or year of publication from the time of their inception through 25 April 2023. Data were collected from the following databases: CNKI, VIP, Wanfang Database, CBM, PubMed, Web of Science, and Cochrane Library. Several keywords were used in the search strategy, including acupuncture, electroacupuncture, scalp and auricular acupuncture, systematic review assessment, meta-analysis, and PD. We manually looked through articles mentioning relevant systematic reviews and meta-analyses, as well as their reference lists, in addition to the primary database search. For the comprehensive search strategy, PubMed uses the following retrieval strategy, see Supplementary material 1.

#4 #1 AND #2 AND #3

#3 Search: ((((((((System evaluation) OR (systematic review)) OR (system assessment))) OR (Meta analysis)) OR (systematic assessment)) OR (systematic evaluation)) OR (systematical review)) OR (evaluation of system)

#2 Search: ((Parkinson's disease) OR (parkinsonian)) OR (parkinson disease)

#1 Search: ((((acupuncture) OR (electroacupuncture)) OR (Electric acupuncture)) OR (scalp acupuncture)) OR (warm acupuncture).

### Inclusion and exclusion criteria

The following were the inclusion criteria: (i) type of study: published systematic reviews/meta-analyses/systematic reviews of acupuncture for PD were not limited to study region or language; (ii) subjects: any patient who exhibited symptoms consistent with PD, regardless of sex, age, race, onset of the disease, prognosis, or severity; (iii) intervention measures: treatment groups included acupuncture treatments such as electroacupuncture, scalp acupuncture alone or used in conjunction with other therapy modalities; other acupuncture techniques, such as moxibustion and injection of acupuncture points, were not included. The baseline of the control group was similar to that of the treatment group, if individuals did not receive nonacupuncture treatments, such as drugs or a blank control; and (iv) outcome measures: all types of outcomes related to PD and the use of valid measurement tools.

The following were the exclusion criteria: (i) published research that was reported in duplicate; (ii) nonsystematic reviews, including narrative reviews, reviews of reviews, summaries of reviews, dissertations, conference papers, conference abstracts, research protocols, opinion pieces, and letters to the editor; (iii) literature that contrasted two acupuncture techniques; (iv) studies with insufficient data or missing entire texts; (v) research involving animal studies; or (vi) studies in which access to the complete text was not possible.

### Study selection

The titles and abstracts of the remaining records were evenly divided among the researchers and individually evaluated by two researchers after all articles were imported into Endnote software, duplicate records were eliminated, and the remaining records were evaluated, During the screening process, there were no language restrictions, and the two researchers were proficient in both Chinese and English. If encountering languages other than Chinese and English, NetEase Youdao translation software was used. With the senior author (SYZ), all differences were discussed and a consensus was reached. After the initial screening of the study title and abstract, two researchers independently evaluated all the texts of any potentially eligible studies. The reasons for exclusion were noted. Any differences that occurred during the full text review process were also resolved by discussion and agreement with the other researcher (SYZ).

### Data extraction and rigorous evaluation

One researcher independently extracted the data, while a second researcher used a preset form to verify the information. The following data were extracted according to the features of the study: (i) general bibliographic data of the review, such as the name of the author, the year of publication, the number of authors and the total number of significant research publications cited; (ii) the conclusions of systematic reviews or meta-analyses, which were established by qualitative evidence summarization or quantitative evidence analysis (22); (iii) information on the search method (such as the start and end dates of the pilot study, the number of databases used, and other searches); (iv) the inclusion standards of the review (such as age, sex, group characteristics, study methodology, and language); (v) quality of the review (including compliance with the PRISMA or MOOSE criteria and research protocols reported in PROSPERO) and the methodological quality of the primary studies included in the review (i.e., tools used and classification of the main studies).

The results were independently evaluated by two researchers and disagreements were resolved by discussion or by a third researcher (SYZ). Using AMSTAR2, a review evaluation strategy consisting of 16 items that were individually graded as yes, no, and sometimes with a partial yes; all included studies were critically evaluated. The crucial components were 2, 4, 7, 9, 11, 13, and 15. The methodological quality was strong when the results were precise, and there was only one nonkey element flaw. When there were multiple nonkey item flaws but no major flaws, the methodological quality was of a moderate level, and the results were considered correct. If a key item was lacking, independently of a missing nonkey item, the methodological quality was considered poor, and the systematic review's results might not be precise or complete. The methodological quality of the systematic review was considered very poor if there were many key element defects, even in the absence or presence of nonkey element defects, and the findings were considered incorrect and lacking (23).

### Synthesis results

To report the amount of research in each category of data extraction, we conducted numerical analyses. Inclusion criteria (such as sex and age), reporting quality, outcome measures, and systematic review and meta-analysis characteristics were a few examples of these categories. The methodological quality was assessed using the AMSTAR2 checklist.

The AMSTAR2 scale contains a total of 16 entries. Each entry is answered either “yes” or “no,” and some entries can be answered as “partial yes.” Entries 2, 4, 7, 9, 11, 13, 15 are key entries. If no items are defective or there is only one non-key item which is defective, the methodological quality is high, and the SR conclusion is judged to be accurate and comprehensive. If there is more than one non-key item defect, but there are no key item defects, the methodological quality is judged to be medium. These results indicate that the conclusions of the SR are considered accurate. If a key item is defective, with or without non-critical item defects, the methodological quality is low, and the conclusions of the SR may not be accurate and comprehensive. If there is more than one key item defect, accompanied or not accompanied by non-critical item defects, the methodological quality is extremely low, and the conclusions of the SR are considered inaccurate and incomplete (24).

### Data analysis

SPSS v.26 was used to perform the analyses. For categorical variables [such as language (inclusion criteria)], numbers and percentages were used, but for continuous variables (such as number of authors), means and standard deviations or medians and ranges were used, and the results are presented in tables and bar graphs. Descriptive methods were used for qualitative data.

### Patients and public involvement

There were no patients nor public directly involved in this review. Only existing data in the literature and the aforementioned sources were used for this study.

## Results

**Figure 1:**
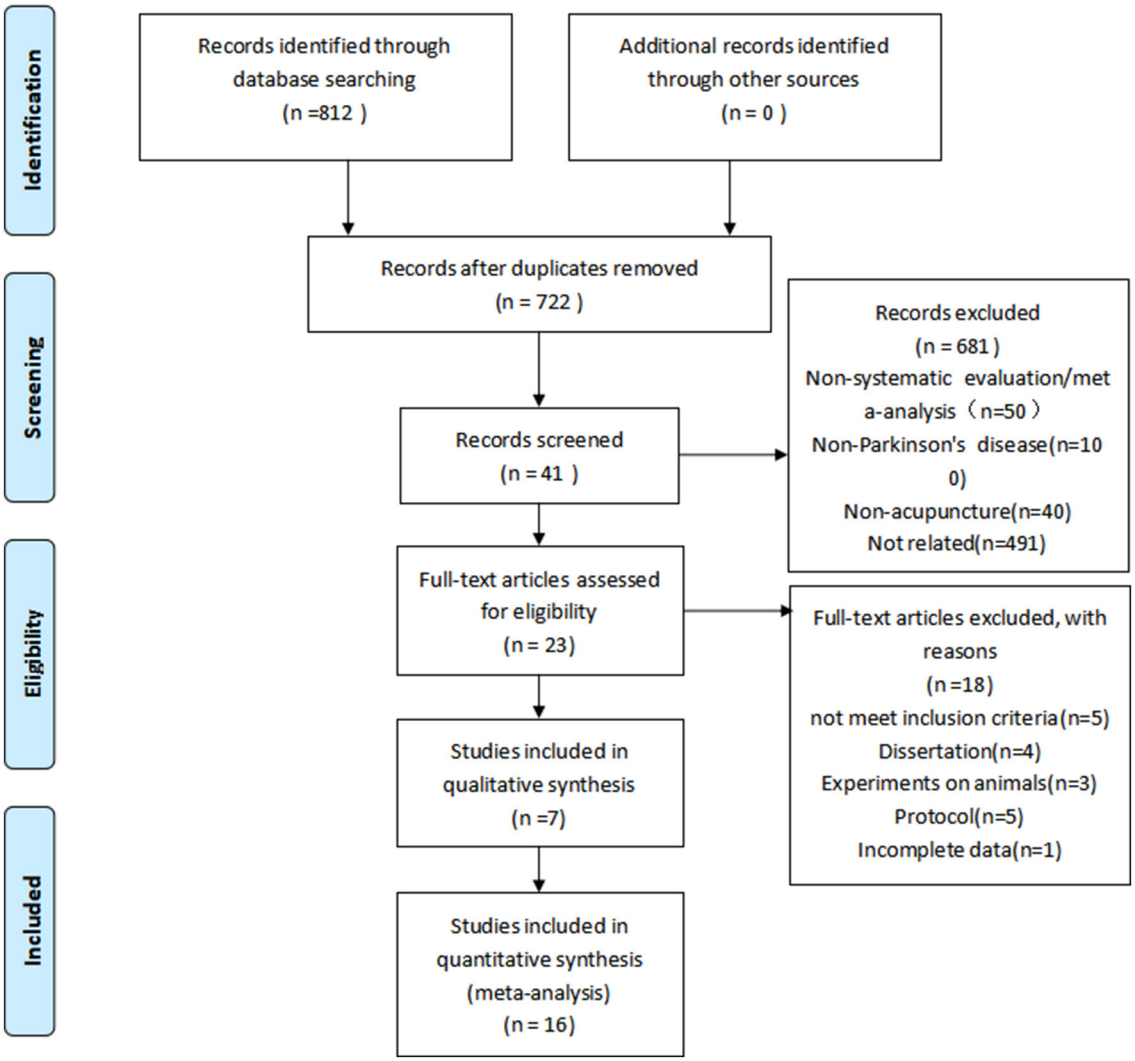
Literature selection process.

After retrieving 812 studies in total, 90 duplicates were eliminated and the remaining 722 study titles and abstracts were also checked. Six hundred eighty-one studies were eliminated after evaluation of the title and abstract. After reviewing all of the full-texts of the remaining 41 articles, we eliminated 18 papers. Therefore, a total of 23 systematic reviews and/or meta-analyses on acupuncture for treatment of PD were incorporated into this scoping review (25–47). Figure 1 shows the search procedure in more detail, and Supplementary material 2 contains a detailed list of exclusions.

### Characteristics of systematic reviews and/or meta-analyses

A total of 23 studies were included (median: 13; range: 1 to 62), including 6 qualitative studies (33.3%) and 12 quantitative studies (66.7%). The studies considered in this review were published between 2008 and 2023, and most of the literature (*n* = 11; 47.8%) was published between 2019 and 2023. Most research (4) was released in 2017 and 2022. Almost all the included articles (82.6%) had more than two authors, with the average number of authors per study being 5.6 ± 2.7 (range: 1 to 9). Table 1 provides a summary of the characteristics of the included studies, and Supplementary material 3 provides a list of the fundamental characteristics of the studies.

**Table 1:** Characteristics of systematic review and meta-analyses included in the review.

**Variable**	**Category**	**Frequency (%)**
Publication year	2008–2013	5 (21.7)
	2014–2018	7 (30.4)
	2019–2023	11 (47.8)
Number of authors	Mean ± SD (range)	5.6 ± 2.7 (1–9)
	Two authors	4 (17.4)
	Multiple authors (>2 authors)	19 (82.6)
Number of studies included	Total (median)	413 (13)
	Mean ± SD (range)	18.0 ± 13.5 (1–62)
Summary findings	Systematic review only	7 (30.4)
	Meta-analysis only	8 (34.8)
	Combination of both (systematic review and meta-analysis)	6 (26.1)
	Meta-analysis and qualitative review	1 (4.3)
	Network meta-analysis	1 (4.3)
Number of databases included in search	Mean ± SD (range)	8.0 ± 3.1 (1–12)
	3–4	2 (8.7)
	5–7	11 (47.8)
	8–10	6 (26.1)
	>10	4 (17.4)
Other search strategies used (not mutually exclusive)	Not reported (No other strategies were used)	8 (34.8)
	Search reference list of included studies	8 (34.8)
	Other additional searches done (online/through reviews/personal database)	3 (13.0)
	Combination of both	4 (17.4)
Search start date	Setting up a database	13 (56.5)
	1950–1979	2 (8.7)
	1980–2009	2 (8.7)
	2010–2023	2 (8.7)
	Not reported	4 (17.4)
Search end date	Before 2010	3 (13.0)
	2010–2014	3 (13.0)
	2015–2019	10 (43.5)
	2020–2022	7 (30.4)
Participant inclusion criteria: Age	No limit	12 (52.3)
	Not reported/stated	11 (47.8)
Participant inclusion criteria: Sex	No limit	11 (47.8)
	Not reported/stated	12 (52.3)
Study design inclusion criteria (not mutually exclusive)	Randomized controlled trials	20 (87.0)
	Randomized controlled design	2 (8.7)
	Randomized controlled trials and Semi-randomized controlled clinical trial and Controlled clinical trials with the word randomized but without a specific description of the randomized method	1 (4.3)
Language inclusion criteria	Two languages	7 (30.4)
	Multiple languages	9 (39.1)
	Not reported	7 (30.4)
Reporting guidelines	Yes	6 (26.1)
	No	17 (73.9)
Registered in PROSPERO	Yes	3 (13.0)
	No	20 (87.0)
Quality assessment of the studies included in the systematic reviews and meta-analyses	Reported but no categorization possible	8 (34.8)
	Reported with categorization	14 (60.9)
	Not reported	1 (4.3)
Quality of the primary studies (*n* = 242) included in the systematic reviews and meta-analyses where quality was reported with categorization (*n* = 14)	High	45 (18.6)
	Moderate	44 (18.2)
	Low	106 (43.8)
	Undetermined (unclear)	47 (19.4)
Quality assessment tools used in the systematic reviews and meta-analyses	Not reported (or not applicable)	2 (8.7)
	Adapted/modified from previous study or tool	3 (13.0)
	Cochrane Collaboration's Tool for Assessing Risk of Bias	15 (65.2)
	Others	3 (13.0)

### Search strategies in systematic reviews

The authors searched 8.0 ± 3.1 literature databases on average for the primary screening (range: 1 to 12). To find the relevant literature, two investigators (8.7%) used three to four databases, 11 investigators (47.8%) used five to seven databases, and six investigators (26.1%) used eight to 10 databases. Thirteen searches (56.5%) included studies from the date of inception of the database, while four studies (17.4%) did not. Ten studies (43.5%) indicated that the date range of the search strategy was between 2015 and 2019.

Instead of screening literature databases, 15 searches (65.2%) relied on other screening techniques. The most frequent additional search method (52.2%) was screening of reference lists of studies that were already eligible for inclusion in the review. Three other additional search strategies (i.e., online, review, and personal database searches) made up the remaining 13.0%. Bibliographic databases alone were used in eight studies (34.8%), while the remainder used alternative search methods.

### Inclusion criteria for systematic reviews and/or meta-analyses

In 12 studies (52.3%), participants of any age were included, while in 11 studies (47.8%) ages were not specified. There were no restrictions on the sex of the participants in 11 (47.8%) studies. Twelve (52.3%) studies omitted information on sex.

Randomized controlled trials were represented in 20 studies (80.0%), and few included the relative follow-up studies (*n* = 2; 8.7%). One review (4.3%) consisted of RCTs, semi-RCTs, and controlled clinical trials, all of which contained the word “randomized” but lacked a detailed explanation of the randomized methodology.

### Quality evaluation of systematic reviews and/or meta-analyses

Only three of the 23 articles included in the analysis provided PROSPERO registration numbers and six (26.1%) of the studies adhered to the reporting requirements for systematic reviews and meta-analyses (such as PRISMA).

Twenty-two studies (95.7%) underwent methodological quality assessment, of which 8 (34.8%) reported unclassified methodological quality assessment and 14 (60.9%) reported classified methodological quality assessment (high, medium, and low quality). Fourteen publications assessed the caliber of 242 original studies, of which 45 (18.6%) had high caliber, 44 (18.2%) had medium caliber, 106 (43.8%) had low caliber, and 47 (19.4%) were ambiguous.

Three studies (13.0%) used adapted or modified tools and 15 studies (65.2%) used the Cochrane risk of bias tool, while 3 studies (13.0%) used other quality assessment tools, such as the Physical Therapy Evidence Database (PEDro) scale, the Cochrane Risk of Bias (ROB) scale, or the quality evaluation suggested by Cochrane Reviewers Handbook 4.2.8 combined with Jadad. A small percentage of studies (8.7%) did not mention using any quality assessment methods.

### Outcomes included in the review

**Figure 2:**
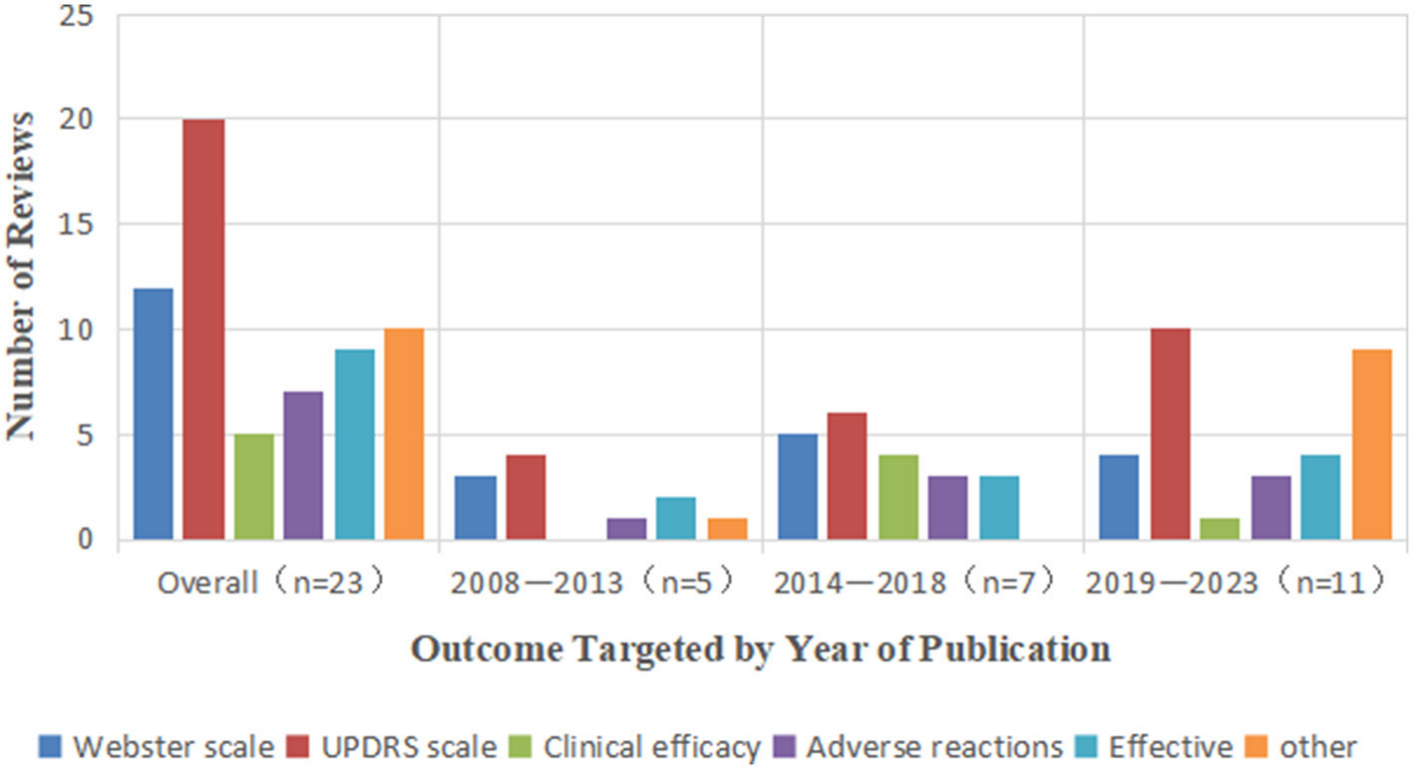
Outcomes by review's year of publication (*n* = 23).

The Webster scale (12/23), UPDRS scale (20/23), clinical efficacy (5/23), adverse reactions (7/23), and efficacy (9/23) were the outcomes that were most frequently analyzed. Studies using Webster and UPDRS scales were included in numerous evaluations. Clinical efficacy was taken into account in five studies, but not in evaluations published before 2012. There was only three study that included outcome measures other than clinical efficacy, and few studies used only the 39 PD questionnaires, the Hamilton Depression Scale and the Mini-Mental State Examination. The results evaluated are shown in Figure 2.

### Quality of the included studies

Table 2 shows the level of excellence of the systematic reviews and meta-analyses as assessed using AMSTAR2 checklist. One study among 23 was of low quality, while the other 22 were of very low quality. In general, almost all studies met the nonkey requirements of Items 6 and the key requirements of Items 9 and 11. Most studies (11/23) or (12/23) had acceptable or sufficient search methods, respectively, for important items. A pre-published research protocol was mentioned in one publication. Only one study included a list of the literature that was eliminated and an explanation for each item on the list. Most studies employed logical tools to assess the risk of bias for each included study.

**Table 2:** Evaluation results on AMSTAR2 scale.

**Included studies**	**Item 1**	Item 2^*^	**Item 3**	Item 4^*^	**Item 5**	**Item 6**	Item 7^*^	**Item 8**	Item 9^*^	**Item 10**	Item 11^*^	**Item 12**	Item 13^*^	**Item 14**	Item 15^*^	**Item 16**
Li (25)	Y	N	N	PY	Y	Y	N	PY	Y	N	Y	N	Y	N	N	N
Miaoxuan (26)	Y	N	N	PY	N	N	N	PY	Y	N	Y	N	N	Y	N	N
Hong-Na (27)	N	N	N	Y	Y	Y	N	PY	Y	N	Y	N	Y	Y	Y	N
Jing (28)	Y	N	N	Y	Y	Y	N	PY	Y	N	Y	N	N	N	Y	N
Lihong (29)	Y	N	N	Y	Y	Y	N	PY	Y	N	Y	N	N	N	N	N
Mohan (30)	N	N	N	PY	Y	Y	N	PY	Y	N	Y	N	Y	N	N	N
Sui (31)	Y	N	N	Y	N	Y	N	N	N	N	Y	N	Y	Y	Y	N
Yanhui (32)	Y	N	N	PY	Y	Y	N	N	Y	N	Y	Y	Y	N	N	N
Sha (33)	Y	N	N	PY	Y	Y	N	PY	Y	N	Y	N	Y	N	N	N
Wen (34)	Y	N	N	Y	Y	Y	N	PY	Y	N	Y	N	Y	N	N	Y
Kwon (35)	Y	Y	N	PY	Y	Y	Y	PY	Y	N	Y	N	Y	N	Y	Y
Lam (36)	Y	N	N	Y	N	Y	N	PY	Y	N	NO Data consolidation	NO META	N	N	N	Y
Lee (37)	N	N	N	Y	N	Y	N	PY	Y	N	Y	N	Y	Y	N	Y
Lee (38)	Y	N	N	Y	Y	Y	N	Y	Y	N	Y	N	Y	Y	N	Y
Lee (39)	N	N	N	Y	N	Y	N	PY	Y	N	Y	N	N	Y	N	Y
Liu (40)	Y	N	N	PY	Y	Y	N	PY	Y	N	Y	N	Y	N	N	Y
Noh (41)	Y	N	N	Y	Y	Y	N	PY	Y	N	Y	N	Y	Y	N	Y
Qiang (42)	Y	N	N	Y	Y	Y	N	Y	Y	N	Y	N	N	N	N	Y
Sun (44)	Y	N	N	PY	Y	Y	N	PY	Y	N	Y	N	N	N	N	N
Fu (43)	Y	N	N	PY	Y	Y	N	PY	Y	N	Y	N	N	N	Y	N
Pereira (45)	Y	N	N	Y	Y	Y	N	PY	N	N	NO Data consolidation	NO META	N	N	N	Y
Li (46)	Y	N	N	PY	Y	Y	N	PY	Y	N	Y	N	Y	N	Y	Y
Wei (47)	N	N	N	PY	Y	Y	N	PY	Y	N	Y	N	N	N	N	Y

^*^, Key item; Y, Yes; N, No; PY, Partially yes; Item 1: do the study questions and inclusion criteria include PICO? Item 2: Are there pre-published plans? Is there a clear bias between research and programmes? Item 3: Did the author explain the type of study design included? Item 4: Are comprehensive literature retrieval strategies used? Item 5: Were repeated studies screened? Item 6: Do you perform repeated data extraction? Item 7: Do you provide a list of excluded documents and explain why? Item 8: Are the included studies described in detail? Item 9: Were reasonable tools used to assess the risk of bias for each included study? Item 10: Are the funding sources included in the study reported? Item 11: If a meta-analysis is performed, are the results statistically combined using appropriate methods? Item 12: If a meta-analysis is performed, is the impact of bias risk explained in the results? Item 13: If a meta-analysis was performed, is the discussion explaining the effect of the risk of bias? Item 14: Is there a reasonable explanation for heterogeneity in the discussion? Item 15: If quantitative analysis is performed, are publication biases sufficiently investigated and their possible impacts discussed? Item 16: Are there any potential sources of conflicts of interest reported?

Approximately one-fourth of the studies evaluating non-critical domains (participants, interventions, comparisons, and results, 5/23) did not incorporated all PICO components. More than half of the studies (18/23) performed study selection and almost all (22/23) performed data extraction. Many of the included studies were listed (19/23) or indicated complete descriptions (2/23) were available. Seven studies did not identify heterogeneity in the results or investigated variables that regulate heterogeneity in the data, while only one study indicated a low risk of bias in the included studies or highlighted the role of bias in the results. Approximately half of the studies (12/23) disclosed potential conflicts of interest.

## Discussion

This was the first scoping review of a systematic review and meta-analysis of the application of acupuncture for the treatment of PD to the best of our knowledge. This review covered the breadth of acupuncture treatment due to our thorough search strategy, the rigorous screening of reviews against the PRISMA-ScR checklist (21), the rigorous evaluation of the included reviews using the AMSTAR 2 tool, and the inclusion of treatments related to acupuncture, such as bee venom needles.

Twenty-three systematic reviews and meta-analyses including 20 RCTs (87.0%); 2 randomized controlled studies (8.7%), randomized controlled trials, semi-randomized controlled clinical trials, and controlled clinical trials; and 1 (4.3%) study with the word “randomization” but no specific description of the randomization method were included. A total of 25,159 patients were included, with no restrictions on sex or age. These patients were diagnosed with PD, idiopathic PD, or primary PD. In 76 (18.4%) studies and in 20 (87.0%) RCTs acupuncture was used exclusively, while in the remaining studies, acupuncture was used in conjunction with other therapies, although, the practitioners of the interventions were not disclosed. Only 7 studies, or 30.4%, documented negative effects. Ebster, UPDRS, clinical efficacy, adverse reactions, and effective were used to quantify the results. The UPDRS scale is the scale that was the most frequently used. Only 8 studies were not disclosed and most studies used different search techniques. However, the other included RCTs may not have been complete because they were only manually searched for references or were obtained from other databases, which may have had an impact on the final results. A few studies conducted age and sex subgroup analyses, and nearly half of the studies did not record either the age or sex of the individual with PD. Furthermore, depending on the patient's age and sex, as well as outcome indicators, acupuncture treatment may have various outcomes.

The included RCTs demonstrated the extensive and ongoing clinical research on acupuncture treatment for PD. Although treated cases offered evidence of success in clinical practice, this evidence could not be regarded as a proof of efficacy (48), and most RCTs (63.2%) were of low or ambiguous quality. The results of the included systematic review and meta-analysis demonstrated that acupuncture and combined treatment of PD had considerable efficacy and significant improvement in symptoms, although the 23 included studies exhibited methodological flaws. The 22 research projects evaluated on the AMSTAR scale may be impacted by the lack of a preceding study design. Eleven studies did not take conflicts of interest into account, making it difficult to determine whether the studies had any possible biases, such as the impact of artifacts related to conflict of interest on the evaluation of results. It is important to proceed cautiously with the results because there is no adequate proof supporting the efficacy of acupuncture as treatment for PD due to methodological flaws.

This study also contains certain limitations that should be considered. First, we included systematic review and a meta-analysis as part of our search process. The use of terms “systematic review” or “meta-analysis” or both in the study titles is recommended by the PRISMA statement (49), although previously published studies could have been overlooked. Second, only systematic reviews and meta-analyses written in Chinese and English were searched, excluding systematic reviews and meta-analyses written in other languages; Third, although efficacy was evaluated, there may be less trust in the findings because the included studies varied in terms of participants, interventions, controls, and results. Fourth, most of the included trials lacked follow-up information that could have provided additional details on long-term outcomes and patient quality of life, making it difficult to assess efficacy.

Despite promising results, it is challenging to draw definitive conclusions about the efficacy of these therapies for PD due to variations in study design and the shortcomings of intervention processes, such as those used to administer the interventions. There are still gaps in knowledge and quality of evidence required to support the use of acupuncture for the treatment of PD due to the methodological flaws of these studies, including limited justification for interventions and symptoms, variations in measurement techniques, variations in study designs and intervention doses, and general limitations in internal and external validity. We hope to focus on improving research design and methods in acupuncture treatment of Parkinson's disease in the future, in order to increase the credibility of research results.

## Conclusions

Acupuncture therapy has received significant research interest both nationally and internationally and appears to be a potential alternative therapy for the treatment of PD, with no unique adverse events recorded compared to other forms of treatment. This study comprehensively evaluated the quality and research methods included in systematic reviews and meta-analyses, discussed the study design, outcome measures, and limitations, which limited the efficacy of the evidence provided; however, no definitive conclusions can be drawn on the evidence supporting acupuncture treatment for PD at this stage. The conclusions of this study are based on the results of the literature analysis without subjective judgment, but there may be some bias due to the quality and quantity of the literature. But the lack of scientific evidence does not necessarily mean that treatment is ineffective (50). We hope that by providing updated evidence on methodological issues in systematic reviews and meta-analyses of acupuncture for PD, this study will attract more attention and stimulate further discussion and, we hope, better solutions to address discussions in this filed. These findings may also provide information for the design and evaluation of efficacy of future studies.

## Data availability statement

The original contributions presented in the study are included in the article/Supplementary material, further inquiries can be directed to the corresponding author.

## Author contributions

All authors listed have made a substantial, direct, and intellectual contribution to the work and approved it for publication.
