# Supplementary material for: Research methods and efficacy of acupuncture in the treatment of Parkinson's disease: a scoping review of systematic reviews and meta-analyses
Source: Front Neurol. 2023 Jun 2;14:1196446. doi: 10.3389/fneur.2023.1196446 (PMC10272821; doi:10.3389/fneur.2023.1196446)
Supplement: Supplementary file 1 [file Data_Sheet_1.docx]

**Supplementary Material 1: Search strategies for each database**

**VIP Chinese Periodical Service Platform:**

((((((((Any field = acupuncture OR any field = acupuncture) OR any field = acupuncture method) OR any field = electroacupuncture) OR any field = scalp acupuncture) OR any field = warm acupuncture) OR any field = warm acupuncture) AND (((((((((((any field = Parkinson's disease OR Any field =parkinson's disease (OR any field =parkinsonian) OR any field = Parkinson's disease (OR any field = Parkinsonian) OR Arbitrary field = Parkinsonism) OR arbitrary field = Parkinsonism) OR arbitrary field = parkinsonism) AND (((((((((arbitrary field = systematic review OR arbitrary field =evaluation of system) OR any field =system assessment) OR any field =system evaluation) OR any field =systematic assessment) OR any field =systematic evaluation) OR any field =systematic review) OR any field =systematic review) OR any field =systematic review) OR any field =meta analysis))

**Wanfang Data Knowledge Service Platform:**

All :(acupuncture or acupuncture or acupuncture or electroacupuncture or scalp acupuncture or warm acupuncture or warm acupuncture) and all :(PD or PD or parkinsonism or Parkinsonism or parkinsonism or parkinsonism or parkinsonism) and All :(Systematic review or systematic review or Meta-analysis)

**CNKI:**

(((( (topic = = acupuncture needle or title or v_subject = extension (acupuncture) or in both English and Chinese title = extension (acupuncture) in both English and Chinese) or topic (= = acupuncture acupuncture or title, or v_subject = extension (acupuncture) or in both English and Chinese title = extension (acupuncture) in both English and Chinese) ) or ( (topic = stitch or title = stitch or v_subject = extension (stitch) or in both English and Chinese title = extension (stitch) in both English and Chinese) or (topic = cupping or title = the curative or v_subject = extension in both English and Chinese (including) or title = expansion (including) in both English and Chinese) )) or ( (topic or title = = head needle or v_subject = extension in both English and Chinese (pin) or the title = extension (pin) in both English and Chinese) or (= subject or title = wen wen needle or v_subject = extended temperature (needle), or in both English and Chinese title = extended temperature (needle) in both English and Chinese) )) or (topic = acupuncture or title = temperature acupuncture or v_subject = extension in both English and Chinese acupuncture (temperature) or title = extension (warm acupuncture) in both English and Chinese)) and ((((( (Subject = PD or title= PD or v_subject= Chinese and English extension (PD) or title= Chinese and English extension (PD)) or (subject= PD or title= Parkinson's disease or v_subject= Chinese and English extension (PD) or (Parkinson's disease)) or ( (Subject = Parkinsonism or title= Parkinsonism or v_subject= Chinese-English extension (Parkinsonism) or title= Chinese-English extension (Parkinsonism)) or (subject= Parkinsonism or title= Parkinsonism or v_subject= Chinese-English Extended (Parkinson's syndrome) or title= Chinese/English extended (Parkinson's syndrome)))) or ( (Subject = Parkinson's disease or title= Parkinson's disease or v_subject= Chinese and English extension (Parkinson's disease) or title= Chinese and English extension (Parkinson's disease)) or (subject= Parkinson's syndrome or title= Parkinson's syndrome or v_subject= Chinese and English extension (Parkinson's syndrome) Syndrome) or title= Chinese English extension (Parkinson's syndrome)))) or ( (Subject = Parkinsonism or title= Parkinsonism or v_subject= Chinese and English extension (Parkinsonism) or (subject= Parkinsonism or title= Parkinsonism or v_subject= Chinese and English extension (Parkinsonism)) Or title= Chinese/English extension (Parkinsonism)))) or ( (Subject = Parkinson's disease or title= Parkinson's disease or v_subject= Chinese-English extension (PD) or title= Chinese-English extension (PD)) and (subject= Parkinson's disease or title= Parkinson's disease or v_subject= Chinese-English extension (PD) or t itle= Chinese and English extension (Parkinson)))) and (( (Subject = systematic review or title= Systematic review or v_subject= Chinese-English extension (Systematic Review) or title= Chinese-English extension (Systematic review)) or (subject= systematic review or title= systematic review or v_subject= Chinese-English extension (systematic review) or ti tle= Chinese and English extension (systematic review)) or (subject= meta-analysis or title= meta-analysis or v_subject= Chinese and English extension (meta-analysis) or title= Chinese and English extension (Meta-analysis)) (fuzzy matching)

**China Biology Medicine disc:**

((" evaluation system "[all fields: intelligent] OR" systematic review "[all fields: intelligent]) OR (" meta-analysis" [weighted: extension])) AND ((" Parkinson's "[all fields: intelligent] OR" Parkinson's "[all fields: intelligent] the OR "Parkinson's syndrome "[all fields: intelligent] OR" Parkinson's syndrome "[all fields: intelligent] OR "Parkinsonism "[all fields: intelligent] OR" Parkinsonism "[all fields: intelligent] OR "Parkinson's syndrome "[all fields: intelligent] OR" Parkinson's disease "[all fields: intelligent] OR "Parkinson's" [all fields: intelligent]) OR (" Parkinson's "[weighted: extension])) AND ((" wen needle [all fields: intelligent]" OR "warm acupuncture" [all fields: intelligent]) OR (" wen acupuncture "[weighted: extension]) OR (" pin" [weighted: extension]) OR (" cupping "[weighted: extension]) OR (" acupuncture" [common fields: intelligent]) OR (" stitch "[common fields: intelligent]) OR (" acupuncture" [not weighted: extension]) OR (" acupuncture "[weighted: extension]))

**Pubmed：**

**#4 #1 AND #2 AND #3**

**#3 Search: **((((((((System evaluation) OR (systematic review)) OR (system assessment)) ) OR (Meta analysis)) OR (systematic assessment)) OR (systematic evaluation)) OR (systematical review)) OR (********evaluation of system)****

**#2 Search: **((Parkinson's disease) OR (parkinsonian)) OR (parkinson disease)****

**#1 Search: **((((acupuncture) OR (electroacupuncture)) OR (Electric acupuncture)) OR (scalp acupuncture)) OR (warm acupuncture)****

**Web of science:**

**#4 ((#1) AND #2) AND#3**

**#3 (((((((ALL=(System evaluation)) OR ALL=(systematic review)) OR ALL=(system assessment)) OR ALL=(Meta analysis)) OR ALL=(systematic assessment)) OR ALL=(systematic evaluation)) OR ALL=(systematical review)) OR ALL=(evaluation of system)**

**#2 ((((ALL=(acupuncture)) OR ALL=(electroacupuncture)) OR ALL=(Electric acupuncture)) OR ALL=(scalp acupuncture)) OR ALL=(warm acupuncture)**

**#1 ((ALL=(Parkinson's disease)) OR ALL=(parkinsonian)) OR ALL=(parkinson disease)**

**Cochrance library：**

**#1 MeSH descriptor: [Parkinson Disease] explode all trees**

**#2 (Parkinson's disease):ti,ab,kw OR (parkinsonian):ti,ab,kw**

**#3 #1 OR #2**

**#4 MeSH descriptor: [Systematic Review] explode all trees**

**#5 evaluation):ti,ab,kw**

**#6 (systematical review):ti,ab,kw OR (evaluation of system):ti,ab,kw**

**#7 #4 OR #5 OR #6**

**#8 MeSH descriptor: [Acupuncture] explode all trees**

**#9 MeSH descriptor: [Electroacupuncture] explode all trees**

**#10 (Electric acupuncture):ti,ab,kw**

**#11 MeSH descriptor: [Electroacupuncture] explode all trees**

**#12 (warm acupuncture):ti,ab,kw**

**#13 #8 OR #9 OR #10 OR #11 OR #12**

**#14 #3 AND #7 AND #13**
