# Supplementary material for: Research methods and efficacy of acupuncture in the treatment of Parkinson's disease: a scoping review of systematic reviews and meta-analyses
Source: Front Neurol. 2023 Jun 2;14:1196446. doi: 10.3389/fneur.2023.1196446 (PMC10272821; doi:10.3389/fneur.2023.1196446)
Supplement: Supplementary file 2 [file Data_Sheet_2.docx]

**Supplementary Material 2：Exclusion list**

**No-systematic review (50 articles)**

[1] Jiang Xuemei, Huang Yong, Wang Shengxu. Effect evaluation and thinking of acupuncture on Parkinson's disease [J]. Chin J Med,2005(07):1240-1242.

[2] Wang Feng, Liu Li, Li Xiaoling, Cao Danna, Liu Li, Qu Bing. Evaluation of the changes of substantia nigra dense zone in Parkinson's disease treated with acupuncture by susceptibility weighted imaging [J]. Clinical Journal of Acupuncture and Moxibustion,2012,28(06):24-26.

[3] Ren Hongyu, Wang Xingxing, Zheng Yu, Huang Yong. Study on the rules of acupointselection in the treatment of Parkinson's Disease with acupuncture and moxibustion [J]. Shanghai Journal of Acupuncture and Moxibustion,2015,34(01):70-72.

[4] Sun Yanan, Yu Changhe, Huang Xiaobo. A systematic review of the treatment of Parkinson's disease with traditional Chinese medicine [J]. Beijing Traditional Chinese Medicine,2016,35(05):430-435.

[5] Li Lihong, Zhang Haifeng, Chen Sheng, Wang Fan, Qi Liang, Wang Le. Effect of intradermal therapy on relieving constipation symptoms in patients with Parkinson's disease [J]. Journal of Medical Postgraduate,2017,30(07):762-766.

[6] Lin Wan-min, Xu Zhi-feng, Peng Xiao-wen. Efficacy evaluation of 3.0T MR Quantitative magnetization mapping in the treatment of Parkinson's disease with acupuncture [J]. Chinese Journal of Medical Imaging,2018,28(04):521-524.

[7] Wang Zheng, Zhang Hu, Zhang Dawei. Clinical efficacy evaluation of acupuncture combined with levodopa and benserazide in the treatment of Parkinson's syndrome [J]. Journal of Hubei University of Chinese Medicine,2019,21(05):82-85.

[8] Liu Xingan, Zhao Xia. Clinical observation of Dihuang Yinzi combined with acupuncture in the treatment of Parkinson's disease [J]. Modern Distance Education of Chinese Traditional Medicine,2019,17(14):54-55.

[9] jing wang, Wang Anlong, xiao-ming fan, wang Ceng, ting-ting sun, Li Lihong. Evaluation of the effect of transcranial ultrasound on Parkinson's disease model rats treated with electroacupuncture [J]. Chin J Med Ultrasound (Electronic Edition),2020,17(01):70-75.

[10] Anming Lu, Wei Feng, Yujiao Yuan, Jiaqi Wei, Jing Lin, Tong Wang, Jun Liang. Clinical research progress of acupuncture in the treatment of Parkinson's disease motor symptoms [J]. World Traditional Chinese Medicine,2021,16(02):339-345.

[11] Wei Yiqiong, Lai Lisha, Xue Dan, Cheng Qiusheng. Evaluation of the changes of substantia nigra dense zone in Parkinson's disease treated with acupuncture by susceptibility weighted imaging [J]. J Practical Med,2021,37(10):1342-1345.

[12]Jiang F, Yang T, Yin H, Guo Y, Namba H, Sun Z, Asakawa T. Evidence for the Use of Acupuncture in Treating Parkinson's Disease: Update of Information From the Past 5 Years, a Mini Review of the Literature. Front Neurol. 2018 Jul 25;9:596.

[13]Huang J, Qin X, Cai X, Huang Y. Effectiveness of Acupuncture in the Treatment of Parkinson's Disease: An Overview of Systematic Reviews. Front Neurol. 2020 Aug 25;11:917.

[14]Cao L, Li X, Li M, Yao L, Hou L, Zhang W, Wang Y, Niu J, Yang K. The effectiveness of acupuncture for Parkinson's disease: An overview of systematic reviews. Complement Ther Med. 2020 May;50:102383.

[15]Sun XB, Sun ZR, Yin HN, Liu SP. [Progress of researches on mechanisms of acupuncture in treatment of Parkinson's disease]. Zhen Ci Yan Jiu. 2021 Nov 25;46(11):973-9. Chinese.

[16]Otayza J, Juri C. Is acupuncture an alternative for the treatment of Parkinsons Disease? Medwave. 2018 May 3;18(3):e7198. Spanish, English.

[17]Han QQ, Fu Y, Le JM, Pilot A, Cheng S, Chen PQ, Wu H, Wan GQ, Gu XF. Electroacupuncture may alleviate behavioral defects via modulation of gut microbiota in a mouse model of Parkinson's disease. Acupunct Med. 2021 Oct;39(5):501-511.

[18]Shen X, Xie YY, Chen C, Wang XP. Effects of electroacupuncture on cognitive function in rats with Parkinson's disease. Int J Physiol Pathophysiol Pharmacol. 2015 Dec 13;7(3):145-51. PMID: 26823963; PMCID: PMC4697670.

[19]Shulman LM, Wen X, Weiner WJ, Bateman D, Minagar A, Duncan R, Konefal J. Acupuncture therapy for the symptoms of Parkinson's disease. Mov Disord. 2002 Jul;17(4):799-802. doi: 10.1002/mds.10134. PMID: 12210879.

[20]Doo KH, Lee JH, Cho SY, Jung WS, Moon SK, Park JM, Ko CN, Kim H, Park HJ, Park SU. A Prospective Open-Label Study of Combined Treatment for Idiopathic Parkinson's Disease Using Acupuncture and Bee Venom Acupuncture as an Adjunctive Treatment. J Altern Complement Med. 2015 Oct;21(10):598-603.

[21]Wang SJ, Ma J, Gong YX, Wang YC, Zeng XL, Liang Y, Sun GJ. [Effect of electroacupuncture intervention on ERK 1/2 signaling and TNF-α and IL-1β protein levels in the substantia Nigra in rats with Parkinson's Disease]. Zhen Ci Yan Jiu. 2014 Dec;39(6):456-60. Chinese. PMID: 25632569.

[22]Schröter M. Evidenz verliert sich in der methodischen Diversifizierung–das Beispiel Akupunktur bei Morbus Parkinson[J]. Deutsche Zeitschrift für Akupunktur, 2022: 1-3.

[23] Huang Yanxi, Jiang Ling, Lin Yanhua, Jiang Cangzhou. Clinical observation on 35 cases of Parkinson's disease combined with sleep disorders treated with scalp acupuncture combined with shallow acupuncture [J]. J Chinese Medicine,2023,64(4):358-364.

[24] Xu Junjun, Liu Chunhua. Clinical study of scalp acupuncture, rehabilitation training combined with levodopa and benserazide capsule in the treatment of Parkinson's disease [J]. New Traditional Chinese Medicine,2023,55(4):158-162.

[25] Li Yuxia, Luo Zhenglong, Yin Lei, Gu Meijuan, Yang Xinglong, Ren Hui. Advances in non-pharmacological and non-surgical treatment of Parkinson's disease [J]. Int J Gerontology,2023,44(2):233-236.

[26] Zhu Chan, Han Xuke, Yao Chengjiao, Zhang Qiang, Liu Jing, Shao Ming. Acupuncture treatment of Parkinson's disease: the mechanism of action shown by animal experiments [J]. Chinese Tissue Engineering Research, 222,26(8):1272-1277.

[27] Zhang Jianbo, Han Bing, Wei Ruipeng. Clinical observation of acupuncture in the treatment of Parkinson's disease with Yin deficiency of liver and kidney [J]. Journal of Gannan Medical College, 2021,42(5):454-457.

[28] Yu Jiajia. Clinical observation of Xingnao Kaiqiao acupuncture combined with repetitive transcranial magnetic stimulation in the treatment of Parkinson's disease [J]. Guangming Traditional Chinese Medicine, 222,37(4):659-662.

[29] Kang Jian-lou, Zhan Jia-milian, Chen Jian, Lin Xiu-jie. Effect of warm acupuncture combined with r-TMS on patients with Parkinson's disease complicated with swallowing dysfunction [J]. Chinese and Foreign Med Research, 2012,20(21):157-160.

[30] Wang Shuo, Liu Chong-yue, Bai Yan, Wang Shun. Clinical research status of acupuncture and moxibustion in the treatment of tremor-type Parkinson's disease [J]. Chinese Journal of Clinical Medicine, 2012,34(5):972-976.

[31] Yang Miaolin, Feng Weixing, Yan Feifei. Mechanism and clinical research progress of acupuncture and moxibustion in the treatment of dysphagia in Parkinson's disease [J]. International Journal of Chinese Materia Medica, 2021,44(10):1192-1195.

[32] Li Shen-wei, WANG Shun. Research progress of acupuncture and moxibustion in the treatment of Parkinson's disease sleep disorders based on intestinal flora [J]. Chinese Journal of Traditional Chinese Medicine, 2021,37(2):304-310.

[33] Liu Jingyi, Zhang Zichen, Zhao Yadan, Chen Haixia, Cui Yinjie, Xu Zifang. Research progress on clinical efficacy and mechanism of acupuncture in the treatment of Parkinson's disease [J]. International Journal of Traditional Chinese Medicine and Chinese Materia Medica, 2021,44(11):1323-1326+F0003.

[34] ZHU Q.Clinical observation on the treatment of Parkinson's disease with acupuncture at Jiaji point combined with acupoint catgut embedding [J]. Modern distance education of traditional Chinese Medicine in China, 2020,20(21):124-125.

[35] Wang Shun, Li Shouli, Li Yuan, Bai Yan. Research overview of acupuncture and moxibustion on the comorbidity of depression and constipation in Parkinson's disease [J]. Chinese Journal of Traditional Chinese Medicine, 2012,50(11):47-50.

[36] Zhao Yingqian, Kang Kaiwen, Liu Qi, Ma Xue, Li Jie, Lu Gang, Wang Qiang. Effect of electroacupuncture intervention on the expression of tyrosine hydroxylase and oxidative stress factors in the substantia nigra of Parkinson's disease model mice [J]. Shanghai Journal of Acupuncture and Moxibustion, 2021,41(5):521-527.

[37] Li Yu, Liu Yanni, Wang Dou, Li Tao, Yan Yongmei. Clinical research progress of traditional Chinese medicine in the treatment of Parkinson's disease constipation [J]. International Journal of Traditional Chinese Medicine and Traditional Chinese Medicine, 2021,44(8):950-953.

[38] Yang Mei, Xie Qin, Zhu Ronghua, Zhong Li. Xingimagingshen Yizhu Decoction combined with Tiaoshen acupuncture in the treatment of 42 cases of Parkinson's disease with mild cognitive impairment [J]. Global Chinese Medicine, 2021,15(9):1685-1688.

[39] Ma Xue, Wang Qiang, Wang Yuan, Yuan Wei, Liu Zhibin, Qiao Haifa. Effect of early electroacupuncture intervention on the expression of ionized calcium adaptor protein-1 and tumor necrosis factor-α in Parkinson's disease mice [J]. Acupuncture Research, 222,47(11):993-998.

[40] Zhao Yingqian, Kang Kaiwen, Liu Qi, Ma Xue, Li Jie, Lu Gang, Wang Qiang. Effect of electroacupuncture intervention on the expression of tyrosine hydroxylase and oxidative stress factors in the substantia nigra of Parkinson's disease model mice [J]. Shanghai Journal of Acupuncture and Moxibustion, 2021,41(5):521-527.

[41] Li Yu, Liu Yanni, Wang Dou, Li Tao, Yan Yongmei. Clinical research progress of traditional Chinese medicine in the treatment of Parkinson's disease constipation [J]. International Journal of Traditional Chinese Medicine and Traditional Chinese Medicine, 2021,44(8):950-953.

[42] Yang Mei, Xie Qin, Zhu Ronghua, Zhong Li. Xingimagingshen Yizhu Decoction combined with Tiaoshen acupuncture in the treatment of 42 cases of Parkinson's disease with mild cognitive impairment [J]. Global Chinese Medicine, 2021,15(9):1685-1688.

[43] Ma Xue, Wang Qiang, Wang Yuan, Yuan Wei, Liu Zhibin, Qiao Haifa. Effect of early electroacupuncture intervention on the expression of ionized calcium adaptor protein-1 and tumor necrosis factor-α in Parkinson's disease mice [J]. Acupuncture Research, 222,47(11):993-998.

[44] Guo Jie, Zhao Yingqian, Li Hua, Zhang Gaiyue, Lu Yuanrong, Guo Panpan, Wang Qiang. Effect of electroacupuncture on synaptic plasticity and complement-dependent memory impairment in hippocampus of Parkinson's disease dementia mice [J]. Acupuncture Research, 22,47(12):1041-1047.

[45] Zhang Min, Xue Bing, Liu Zijiao, Chen Zhihan, Wang Rui, Ren Yulan. Reevaluation of acupuncture and moxibustion for Alzheimer's disease: a systematic review [J]. World Science and Technology: Traditional Chinese Medicine Modernization, 2021,24(1):113-121.

[46]Li X, Wei W, Wang Y, Wang Q, Liu Z. Global Trend in the Research and Development of Acupuncture Treatment on Parkinson's Disease From 2000 to 2021: A Bibliometric Analysis. Front Neurol. 2022 Jul 8;13:906317.

[47]Song LZ, Li Y, Qian X, Xu N, Yu Z, Dai JW, Xu B, Hu XM. Parkinson's disease constipation effect of electroacupuncture at ST25 through colonic motility and enteric neuropathology. Front Neurol. 2023 Jan 11;13:1092127.

[48] Song LZ, Xu N, Yu Z, Yang H, Xu CC, Qiu Z, Dai JW, Xu B, Hu XM. The effect of electroacupuncture at ST25 on Parkinson's disease constipation through regulation of autophagy in the enteric nervous system. Anat Rec (Hoboken). 2023Jan 19.

[49]Raoul S, Brissot R, Lefaucheur JP, Nguyen JM, Rouaud T, Meas Y, Huchet A, Razafimahefa N, Damier P, Nizard J, Nguyen JP. Additional Benefit of Intraoperative Electroacupuncture in Improving Tolerance of Deep Brain Stimulation Surgical Procedure in Parkinsonian Patients. J Clin Med. 2022 May 10;11(10):2680.

[50]Sun Y, Li L, Chen Y, Wang L, Zhai L, Sheng J, Liu T, Jin X. Feasibility and positive effects of scalp acupuncture for modulating motor and cerebral activity in Parkinson's disease: A pilot study. NeuroRehabilitation. 2022;51(3):467-479.

**No-parkinson's disease (100 articles)**

[1]Lee MS, Shin BC, Choi SM, Kim JY. Randomized clinical trials of constitutional acupuncture: a systematic review. Evid Based Complement Alternat Med. 2009 Sep;6 Suppl 1(Suppl 1):59-64.

[2] Guo Kui, Chen Shu. Efficacy and safety of massage in the treatment of primary dysmenorrhea: a systematic review [J]. Hunan Journal of Traditional Chinese Medicine,2020,36(05):126-130.

[3] Xu Haiyan, Chen Lin, He Xiaoting, Chen Le, Tan Jin-qu, Yang Xian-yun. Clinical literature research on moxibustion therapy for primary dysmenorrhea based on Meta-analysis [J]. Hunan Journal of Traditional Chinese Medicine,2019,35(03):124-125+128.

[4] Xu Haiyan, Chen Lin, He Xiaoting, Chen Le, Tan Jin-qu, Yang Xian-yun. Clinical literature research on moxibustion therapy for primary dysmenorrhea based on Meta-analysis [J]. Hunan Journal of Traditional Chinese Medicine,2019,35(03):124-125+128.

[5] Ge Li, Jinhua Si, Chen Zhao, Long Ge, Jinhui Tian, Hongcai Shang, Zixin Wang, Xiangling Xu, Shuhe Wang. Chinese Journal of Evidence-based Medicine,2017,17(10):1212-1223.

[6] Qin Ai-ling, Ma Rui-ping, Xiao Wan, Yi Li-juan, Tian Xu. Effect of simple acupuncture and moxibustion related therapy on primary dysmenorrhea: a systematic review [J]. International Journal of Obstetrics and Gynecology,2014,41(04):453-458+461.

[7] Chen Wen, Yu Haihong, Liu Shihong, Huang Wanling, Tian Weizhen. Systematic review of acupuncture and moxibustion for primary dysmenorrhea in China [J]. Chin J Traditional Chinese Medicine,2013,31(02):321-325.

[8]Wei TH, Hsieh CL. Effect of Acupuncture on the p38 Signaling Pathway in Several Nervous System Diseases: A Systematic Review. Int J Mol Sci. 2020 Jun 30;21(13):4693.

[9]Park J Y , Kim Y K , Kim S Y , et al. Acupuncture modulates brain neural activity in patients: a systematic review and meta-analysis[J]. Oriental Pharmacy & Experimental Medicine, 2017, 17(2):1-16.

[10]Leung MC, Yip KK, Ho YS, Siu FK, Li WC, Garner B. Mechanisms underlying the effect of acupuncture on cognitive improvement: a systematic review of animal studies. J Neuroimmune Pharmacol. 2014 Sep;9(4):492-507.

[11] Tan Huiyuan, Dong Mingguo, Li Guangyao, Ruan Qiumei, Yang Chuanghui, Li Min. Systematic review and meta-analysis of acupuncture combined with traditional Chinese medicine in the treatment of dry eye [J]. Journal of Guangzhou University of Chinese Medicine,2023,40(4):1043-1052.

[12] Wan Yutong, WU Guixian. Clinical observation of warming acupuncture and moxibustion in the treatment of senile osteoporosis [J]. Modern Distance Education of Chinese Traditional Medicine,2023,21(8):136-138.

[13] Fang Zhenzhen, Fei Ke, Qian Chen, Zhang Mengyuan, Li Zeyu, Wang Feng. Bibliometric analysis and evaluation of acupuncture assisted gastroscopy [J]. Guangming Traditional Chinese Medicine,2023,38(6):1112-1116.

[14] He Yi-han, Zhao Jing-xia, Zhang Dong-mei, Xiong Jun, Geng Zi-han, Li Chao, Chen Che, Bi Minggang. Report and analysis of National Natural Science Foundation of China in the field of acupuncture and analgesia [J]. Chinese Journal of Acupuncture and Moxibustion Electronic Journal,2023,12(1):1-7.

[15] Bai Lu, Zou Wei, Wang Long. Application analysis of sham acupuncture in clinical research of acupuncture intervention for depressive disorder based on literature [J]. Chinese Journal of TCM,2023,64(6):633-640.

[16] Wang Yifei, Zhai Weihang, Xie Rong, He Jianghong, Rong Peijing. Chinese Journal of Acupuncture and Moxibustion,2023,12(1):19-23.

[17] Xu Yue, Nie Liming. Application progress of optical imaging technology in acupuncture research of traditional Chinese medicine [J]. Chinese Journal of Lasers,2023,50(3):65-77.

[18] Zou Jing, Zeng Science. Clinical application of Zhenqi Acupuncture in common diseases of orthopedics and Traumatology [J]. Massage and Rehabilitation Medicine,2023,14(2):47-49+53.

[19] Qin Zhuzhu, Ye Xinxin, Zheng Xutong, Zhan Chenju. Visualization analysis of knowledge map of acupuncture and moxibustion for constipation based on CiteSpace [J]. General Nursing,2023,21(3):289-295.

[20] Zhang Xuemei, Zhang Wei. Clinical research progress of acupuncture in the treatment of lumbodorsal myofascitis [J]. Modern Distance Education of Chinese Traditional Medicine,2023,21(8):203-205.

[21] Lai Yinjie, Liu Zhenxiu, Li Anhong. Acupoint selection rules of acupuncture for post-stroke motor aphasia based on data mining [J]. Massage and Rehabilitation Medicine,2023,14(2):37-41.

[22] Mingrui Chu, Qingjiang Yang, Beibei Diao, Chenbing Zhang, Saisai Gao, Runchen Yang. Research progress of acupuncture and moxibustion for insomnia [J]. Modern Distance Education of Chinese Traditional Medicine,2023,21(1):197-200.

[23] Li Lifang, Zhou Hongfei, Zhang Wei. Mechanism and research progress of acupuncture in the treatment of limb dyskinesia after stroke [J]. Massage and Rehabilitation Medicine,2023,14(3):53-57.

[24] Yan Haiyu, Hua Jinshuang, Li Hanjie, Zhang Yingya, Chen Fang, Gong Jiajia. Research on therapeutic advantages and evaluation indexes of acupuncture and moxibustion in the treatment of xerophthalmia [J]. World Journal of Traditional Chinese Medicine,2023,18(4):572-575+582.

[25] Dong Han, Huirong Liu, Huanyan Wu, Cuina Yan, Jing Chai, Zihui Yang, Changfang Zhang, Linshan Zhang, Qi Li. Research progress and thinking on the mechanism of acupuncture and moxibustion in the treatment of Crohn's disease [J]. Acupuncture Research,2023,48(2):139-146.

[26] Yin Xuejiao, Jiang Tongfei, Chen Zhaoyi, Song Zhangxiao, Li Bin, Guo Jing. Application of machine learning in predicting the effect of acupuncture for insomnia based on brain functional connectivity [J]. Chinese Medicine Review, 2020,20(8):188-191+196.

[27] Zhang Lin-kun. Research progress of scalp acupuncture therapy for vascular cognitive impairment [J]. Chinese Medicine,2023,12(1):88-92.

[28] Deng Rou, Wu Yanxia, Lu Xuejing. Research progress of acupuncture in the treatment of glaucoma [J]. China Journal of Traditional Chinese Medicine Ophthalmology,2023,33(3):282-285.

[30] Ma Qianhui. Clinical observation of Yangxin Tongmai decoction combined with acupuncture in the treatment of angina pectoris of coronary heart disease [J]. Modern Distance Education of Chinese Traditional Medicine,2023,21(7):124-125.

[31] Ding Jiu force, liu wei, Yang Xiaoyan, Guo Xiaojing, Gu Qingxiang, YueQingYun, 婌 Chen yun. Clinical research progress of acupuncture and moxibustion in the treatment of Sjogren's syndrome [J]. Chinese Journal of Library and Information of Traditional Chinese Medicine,2023,47(1):100-103.

[32] Sun Lina. Clinical observation of Banxia Baizhu Tianma decoction combined with Bagua scalp acupuncture in the treatment of Alzheimer's disease [J]. Modern Distance Education of Chinese Traditional Medicine,2023,21(3):103-105.

[33] Liu Runqing, Wang Shudong. Clinical research progress of acupuncture and moxibustion for Alzheimer's disease [J]. Massage and Rehabilitation Medicine,2023,14(5):73-77.

[34] Sun Jifei, Chen Limei, Wang Zhi, Guo Chunlei, He Jiakai, Gao Deqiang, Fang Jiliang. Research progress on the mechanism and efficacy of acupuncture and moxibustion for depression [J]. World Journal of Traditional Chinese Medicine,2023,18(2):291-295.

[35] Liu Yefang, Chen Xugui, Liang Fufang, Li Yu, Xiong Chan, Qin Erqi, Xiao Wei, Du Longyi, Fu Juanjuan. Research progress on the anti-inflammatory mechanism of acupuncture and moxibustion in the treatment of chronic respiratory diseases [J]. Acupuncture Research,2023,48(2):147-152.

[36] Fu Tiancong, Li Yuxuan, Zhang Runchen, Ma Qingtao, Wang Can, Ma Yajing, Xu Zifang, Guo Yi, Wang Shu, Guo Yang. The Mechanism of brain protection of acupuncture in stroke patients [J]. Chin J Practical Neuropathy,2023,26(3):361-367.

[37] Qiang Xiaoyu, Chen Zhe, Ji Zhaochen, Yang Fengwen, Jin Xinyao. Meta-analysis of the effects of traditional Chinese medicine on pulmonary function in patients with coronavirus disease 2019 (COVID-19) during recovery period [J]. Global Chinese Medicine,2023,16(2):226-233.

[38] Lin Yuqin, Tang Xuxia, He Xiao. Chinese Otorhinolaryngology Head and Neck Surgery,2023,30(2):79-83.

[39] Tao Jing, Yang Xiaofan, Zhao Zhixuan, Li Hong. Experimental study on the therapeutic effect of acupuncture therapy on AD rats and its mechanism [J]. International Medicine and Health Review,2023,29(3):342-347.

[40] LU Kejia, Song Juxian. Research progress on the mechanism of acupuncture and moxibustion regulating autophagy-lysosomal pathway in the treatment of nervous system diseases [J]. Journal of Sun Yat-sen University: Medical Science Edition,2023,44(1):10-17.

[41] Zhang Ying, Wang Dongyan, Huo Hong, Dong Xu. Application progress of microrna in the research of disease mechanism of acupuncture and moxibustion intervention [J]. Journal of Clinical Acupuncture and Moxibustion,2023,39(1):110-114.

[42] Wang Xinru. Preliminary exploration in ancient and modern times -- Acupuncture and moxibustion treatment of emotional diseases [J]. Qingdao Medicine and Health,2023,55(1):75-77.

[43] Ma Yuxuan, Zhu Yixia, Ning Yu, Wang Yanli, Yu Jianjun. Research progress of acupuncture and moxibustion for depression [J]. Clinical Journal of Acupuncture and Moxibustion,2023,39(3):106-109.

Research progress of circulation ischemic vertigo after acupuncture intervention [J]. Guangming Traditional Chinese Medicine,2023,38(2):387-391.

[45] Guo Liang, Bi Yufeng, Ji Changchun, Sun Zhangyin, Sun Hui, Xue Yuhan. Research progress of electroacupuncture in the treatment of primary insomnia [J]. International Journal of Chinese Materia Medica,2023,45(3):381-384+F0003.

[46] Li Yaxue, Wu Minmin, Hu Saiqin, Li Sha, Wang Feng. Clinical efficacy and mechanism of Yuanluo Tongjing acupuncture combined with western medicine in the treatment of amnestic mild cognitive impairment [J]. Chin J Traditional Chinese Medicine,2023,38(3):1379-1383.

[47] Wang Hongbin, Dong Xue, Liu Shu, Guo Wenxuan, Cao Ning, Cui Jianmei, Zhao Shu, Wu Shujuan, Zou Dehui, Yu Yueyue, Li Jianning. Research design of time-effect relationship of acupuncture and moxibustion [J]. Chin J Traditional Chinese Medicine,2023,38(2):698-701.

[48] Xiao-Ji Zhu, Lu-Yi Wu, Han-Dan Zheng, Jia-Yi Chen, Ya-Ying Lin, Hui-rong Liu, Yan Huang, Yuan Lu, Ci-Li Zhou, Huan-gan Wu. Clinical research and acupoint selection rules of acupuncture and moxibustion in the treatment of Crohn's disease in the past 20 years [J]. World Journal of Traditional Chinese Medicine,2023,18(4):482-490.

[49] Li Yixuan, Zhao Jixiao, Meng Yan, Li Miaomiao, Liu Cunzhi, Zhang Tao. Chinese Journal of Basic Medicine of Traditional Chinese Medicine,2023,29(3):442-445.

[50] Zhang Mingxia, Huang Yingchun, Yang Xia, Jia Jin. Effect of electroacupuncture combined with medication and aerobic exercise on stable chronic obstructive pulmonary disease and its influence on exercise cardiopulmonary function [J]. Shanghai Journal of Acupuncture and Moxibustion,2023,42(3):221-226.

[51] Wang S L, Sun Y Z, YU T Y, Zhao G R, SUN Y. Early intervention of "Jiaoji" electroacupuncture can alleviate the progression of amyotrophic lateral sclerosis in mice by regulating Toll-like receptor 4/ nuclear factor-κb signaling pathway in lumbar spinal cord [J]. Acupuncture Research,2023,48(3):287-293.

[52] Li Li, Zhang Yu. Research progress on the mechanism of electroacupuncture alleviating morphine tolerance [J]. China Medicine Guide,2023,20(5):51-54.

[53] Xie Zuyao, Qin Weixuan, Yang Guiqin. Research progress on the mechanism of electroacupuncture in the treatment of knee osteoarthritis [J]. Journal of Liaoning University of Traditional Chinese Medicine,2023,25(1):212-215.

[54] Meng Qiao, Zhifei Wang, Chaoren Tan, Jinggang Dai. Effect of Daoyin therapy on quality of life in patients with stable angina pectoris: a Meta-analysis [J]. J Rehabilitation J,2023,33(1):77-89.

[55] Wang Xingbo, Wang Weiming. Literature analysis of the characteristics of acupuncture and moxibustion diagnosis and treatment of tumor myelosuppression after chemotherapy [J]. Western Chinese Medicine,2023,36(1):101-105.

[56] Zhang Yi-ping, Wang Yu, WU Xiao-ke. Clinical research overview of traditional Chinese medicine therapy in the intervention of spleen and stomach weakness type pregnancy resistance [J]. Chinese National and Folk Medicine,2023,32(2):40-44.

[57] Huang Heqing, Wang Fuchun. Research progress of acupuncture and moxibustion in the treatment of adolescent primary dysmenorrhea in recent 5 years [J]. Chinese Medicine Information,2023,40(1):75-78.

[58] Xu Chunxia, Li Rongjun, Yang Xiaoli, Wang Jian, Yang Yan. Clinical observation on the treatment of ulcerative colitis with Jingjia-Liqiao Decoction combined with acupuncture at Ghost eye Point [J]. Journal of Hunan University of Traditional Chinese Medicine,2023,43(2):338-342.

[59] Gao Xin, Wang Cheng. Research progress of acupuncture and Tuina therapy for cervical hypertension [J]. Inner Mongolia Traditional Chinese Medicine,2023,42(2):153-156.

[60] Ren Yi, Yu Dongdong. Construction of nomogram model of the effect of acupuncture treatment on the prognosis of cervical spondylotic radiculopathy [J]. Chinese Journal of Traditional Chinese Medicine,2023,38(2):415-421.

[61] Yang Jieyi, Lin Xiaoguang, Zhang Zili. Teaching effect of LBL combined with CBL in the treatment of orthopedic diseases with acupuncture and moxibustion [J]. Capital Food & Medicine,2023,30(1):103-106.

[62] Zhang Yunan, Li Yizhen, Dou Zhili, Zhao Lei, Zhang Jiawei, Han Dongran. Real world big data research status, problems and solutions [J]. Chin J Traditional Chinese Medicine,2023,38(2):695-698.

[63] Gu Wei-yu, SHI Xue-hui. A brief analysis of the clinical application of Zhenmen Point according to Acupuncture Dacheng [J]. Journal of Practical Traditional Chinese Medicine Internal Medicine,2023,37(1):20-22.

[64] Xing Jian-fei, SU Qian-yi, WU Hai-xin, Liang Shilu, Yang Qing, Song Zi-yu, Chen Qiao. Research progress on the application of external treatment of traditional Chinese medicine in chronic atrophic gastritis [J]. World Traditional Chinese Medicine,2023,18(1):137-140.

[65] Cui Mengjun, Chen Yiying, Chen Haotian, Liu Cong, Guan Dandan, Chen Li. Research progress of acupuncture and moxibustion for perimenopausal hypertension [J]. Massage and Rehabilitation Medicine,2023,14(5):65-68.

[66] Yu Mingcan, Chen Xinwang, You Yanwen, Hao Li. To explore the effect of electroacupuncture on learning and memory ability in mice with Alzheimer's disease and its mechanism [J]. Journal of Beijing University of Chinese Medicine,2023,46(1):132-140.

[67] Liu Yunting, Wang Shengchun, Ji Peiyuan, Wang Ziqi, Li Yuchao, Qiu Wenchao. Clinical research progress of acupuncture and moxibustion in the treatment of premature ovarian insufficiency [J]. Chinese Sexual Science,2023,32(3):139-142.

[68] Kang Yufen, Zhang Jiayu, Mi Yong. New progress of clinical research on acupuncture and moxibustion in the treatment of generalized anxiety disorder [J]. Progress in Clinical Medicine,2023,13(3):3083-3087.

[69] Zhang Jiayu, Kang Yufen, Mi Yong. New progress of clinical research on acupuncture and moxibustion for insomnia [J]. Progress in Clinical Medicine,2023,13(3):3550-3555.

[70] Lin Huize, Yan Wenqian, Zhang Pingping, Fei Jingwen, Shen Jianghong, Liu Lanping, Wang Xiang, Zhu Kexin, Yang Tao, Yu Jinna. Current research on the outcome indicators of randomized controlled trials of acupuncture and moxibustion for chronic non-specific low back pain [J]. Chin J General Med,2023,26(9):1053-1063.

[71] Jing Lu, Xiaomeng Liu, Ying Chen, Haiping Xu, Mingxin Xue, Jinhai Tang. Clinical study on the improvement of quality of life of breast cancer patients with arthralgia from the perspective of mind and body treatment with acupuncture [J]. World Journal of Traditional Chinese Medicine,2023,18(2):249-254.

[72] Gong Tingting, Wang Chao. Research progress on the mechanism of shallow needling therapy and its modern clinical application [J]. Chinese Journal of Clinical Medicine,2023,35(1):184-188.

[73] Li Pengfei, Wang Yu, Rehemutura Asimu. Clinical study on abdominal acupuncture combined with traditional acupuncture in the treatment of insomnia with deficiency of both heart and spleen [J]. Chinese Medicine Guide,2023,21(9):115-117.

[74] Sun Zhong-ren, Wang Rui-qi, Lu Ying-qi, Yin Hong-Na. Research progress of acupuncture and moxibustion in the treatment of spinal cord injury based on the regulation of MAPK signaling pathway [J]. Chin J Traditional Chinese Medicine,2023,38(3):1172-1175.

[75] Pang Ruikang, Feng Zhuo, He Leitao, Zou Zhuocheng, Xu Fu, He Juejie, Qin Ningjing. Research progress of neural cell apoptosis and acupuncture intervention mechanism after cerebral ischemia-reperfusion [J]. World Traditional Chinese Medicine,2023,18(4):576-582.

[76] Feng Guo, Li Shuangyan, Ye Yong, Tan Chaojian, Yan Jie, Li Li. Thoughts and methods of integrated treatment of cervical vertigo with acupuncture, pushing and Kang: Professor Yan Jie's experience in treating cervical vertigo [J]. Journal of Hunan University of Traditional Chinese Medicine,2023,43(3):528-531.

[77] Zou Yaqi, Ruan Zhizhong. Research progress of acupuncture and moxibustion in the treatment of dry eye syndrome of meibomian gland dysfunction [J]. Acta Medicae Sinica,2023,21(2):69-71.

[78] Gao Yuanjie, Sun Jingqing, Li Bin, Guo Jing, Ji Xu, Yuan Fang. Application of auxiliary examination in the evaluation of the curative effect of fire needling therapy for knee osteoarthritis [J]. Western Chinese Medicine,2023,36(3):151-154.

[79] Song Minmin, Dai Jingyu. Feasibility study of acupuncture and moxibustion in the treatment of lumbar muscle strain in adolescent swimmers [J]. Bulletin of Sports Science and Technology,2023,31(3):224-227.

[80] Wang Zhijie, Zhou Jie, Shen Yan. Research progress on the mechanism of moxibustion on Alzheimer's disease [J]. China Journal of Traditional Chinese Medicine Information,2023,30(3):176-176+177-180,F0003.

[81] Yu Xiaoyan, Liu Yang, Gu Xiaohui, Xu Xu, Shen Shuai, Zhu Wei. Application and evaluation of acupuncture anesthesia and analgesia pump in the field of postoperative analgesia of shoulder joint [J]. Science and Technology Review,2023,41(3):44-50.

[82] Wang Xiaohua, Xu Yanlong. Clinical research progress of moxibustion in the treatment of chronic atrophic gastritis [J]. Asia-pacific Traditional Medicine,2023,19(3):244-248.

[83] Liu Yuling, He Yadi, Lu Xuejing. Theoretical discussion and research status of new abdominal acupuncture in the treatment of insomnia and depression related dry eye [J]. China Journal of Traditional Chinese Medicine Ophthalmology,2023,33(3):278-281.

[84] Sun Yan, Ding Yuan, Wang Linjing. Clinical observation of Sun's Tiaoshen acupuncture combined with cognitive training in the treatment of 34 patients with post-stroke cognitive dysfunction [J]. Chin J TCM,2023,64(5):498-503.

[85] Ruimin Jiao, Wencui Xiu, Boling Li, Xiaoyi Hu, Lanjun Shi, Ziyu Tian, Jiwei Yang, Xiangyu Hu, Weijuan Gang, Xianghong Jing. Acupuncture for chronic eczema: evidence map based on randomized controlled trials, systematic reviews and meta-analyses [J]. World Journal of Acupuncture and Moxibustion: English edition, 222,32(4):261-270.

[86] Gao Kai, Wang Xiaofei, Li Yufu, Zheng Wei, Zheng Xiaofei, Wang Huajun, Li Jia, Li Ling, Gao Yanping. Effect of acupuncture and moxibustion on rehabilitation of shoulder injury: a Meta-analysis and systematic review [J]. Global Chinese Medicine, 22,15(10):1838-1844.

[87] Xiang Yu, Zhang Bei, Ding Xinru, Wu Xing. Meta-analysis of acupuncture therapy in the treatment of juvenile myopia [J]. Clinical Research of Traditional Chinese Medicine, 2012,14(20):136-141.

[88] Qi Hang, Huang Xiaoqi. Meta-analysis of the effect of traditional Chinese medicine acupuncture therapy on patients with stable chronic obstructive pulmonary disease [J]. Shenzhen Journal of Integrated Traditional Chinese and Western Medicine, 2012,32(14):21-24+I0002-I0005.

[89] Dai Lishuang, Wu Mingjuan, Zhao Yan. Clinical observation of Tiaoshen Yizi acupuncture in the treatment of AD with anxiety and depression [J]. Chinese Traditional Medicine Modern Distance Education, 2021,20(10):105-107.

[90] Wu Linna, Ma Qingtao, Song Ruihao, Lin Sheng, Li Guiping. Comparison of the effectiveness of acupuncture and moxibustion therapy and western medicine in the treatment of fibromyalgia syndrome: a network Meta-analysis [J]. Modern Medicine and Hygiene, 2021,38(14):2390-2398.

[91] Yin Bingqi, Yu Jianglin, Wu Junyan, Gu Xinyi. Meta-analysis of acupuncture combined with western medicine in the treatment of Alzheimer's disease [J]. Journal of Practical Chinese Medicine Internal Medicine, 2021,36(5):17-19+I0007,I0008.

[92] Zhao Yanran, Li Yanhui, Ma Qiaolin, Yang Fan, Luo Xiao, Hu Bin. Application progress of clinical efficacy evaluation scale for knee osteoarthritis treated with acupuncture [J]. Acupuncture Research, 222,47(8):734-737+743.

[93] Li Ruoqi, Wan Li, Zi Mingjie, Duan Wenhui, He Liyun, Gao Rongrong. Network Meta-analysis of different acupuncture and moxibustion therapies in the treatment of stable angina pectoris of coronary heart disease [J]. Chinese Acupuncture and Moxibustion, 222,42(12):1431-1438.

[94] Liu Qihong, Ke Xiao, Zhao Peilin, Fang Wenyi, He Yanqin, Hu Lunan, Chen Ying, Xu Yancheng, Lin Limin, Lin Mengying. Meta-analysis of acupuncture and moxibustion in the treatment of slow transit constipation [J]. Journal of Practical Chinese Medicine Internal Medicine, 2021,36(10):11-14+I0009-I0012.

[95] Zeng Jingchun, Lu Lihong, Lu Liming, Tang Chunzhi, Xu Nenggui, Lin Guohua. Systematic review of clinical efficacy and safety of acupuncture and cupping therapy in the treatment of herpes zoster [J]. World Science and Technology: Modernization of Traditional Chinese Medicine, 222,24(11):4347-4357.

[99] Wu Xia, Liu Min, Wang Yuanyuan, Zhao Wenshuo, Zhang Qing. Meta-analysis of acupuncture and moxibustion in the treatment of opioid-related constipation [J]. Chinese Journal of Traditional Chinese Medicine Information, 222,29(6):43-48.

[100] Li Yihan, Xin Qiqi, Zhan Tian, Wang Dianwen, Yan Lei, Wang Xinhui, Zeng Qin, Yu Renhuan. Systematic review of acupuncture and moxibustion in the treatment of primary glomerular diseases [J]. Chinese Journal of Integrated Traditional and Western Medicine Nephrology, 2021,23(11):985-991.

**No-acupuncture (40 articles)**

[1] Wen Xiaodong, Zheng Jinghui, Meng Bing, Zhang Yueling, Wang Chunling. Effect of traditional Chinese medicine on Parkinson's disease: a systematic review [J]. Journal of Liaoning University of Traditional Chinese Medicine,2014,16(07):120-124.

[2] Wentao Zheng, Jia Ouyang, Xu Xu, Zeyu Miao, Ruen Liu. Effect of subthalamic nucleus deep brain stimulation on neuropsychological function in patients with Parkinson's disease: a Meta-analysis [J]. Chin J Neurosurgery,2020,36(07):732-738.

[3] Wang Xuefei, Yuan Dezhi, Zhang Xiaotian, Zhao Qiuye, Li Jinfang. Efficacy and safety of deep brain stimulation combined with drugs in the treatment of Parkinson's disease: a Meta-analysis [J]. Chin J Neuro-Neurosurgery,2019,46(04):358-363.

[4] Wang Chao, Gu Yanxia, Li Tingting, Chen Jutao, Zhang Zheng, Chen Yang, Shi Zhaokun, Qu Yuan, Zhang Zhaohui. Stereotactic brain stimulation versus deep brain stimulation in the treatment of Parkinson's disease: a Meta-analysis [J]. Occupational and Health,2017,33(04):465-469.

[5] Zhang Wei, Li Nan, Zhang Haihong, Ma Lei, Gao Jinjian, Wang Xuelian, Gao Guodong. Effect of subthalamic nucleus and globus pallidus internus electrical stimulation on Parkinson's disease: a Meta-analysis [J]. Chin J Clinical Neurosurgery,2015,20(12):718-723.

[6] Li Jiajia, Teng Wenhui, Wang Naidong. Effect of subthalamic nucleus deep stimulation on Parkinson's disease: a Meta-analysis [J]. Journal of Medical College of Qingdao University,2016,52(02):201-204+208.

Meta-analysis of deep subthalamic nucleus stimulation for Parkinson's disease in China [J]. Lab Med & Cl,2016,13(08):1049-1051+1054.

[8] Xianwenbiao, Pei Zhong, Zhou Xuyu, Chen Ling. Efficacy and safety of bilateral subthalamic nucleus deep brain stimulation in the treatment of Parkinson's disease: a Meta-analysis [J]. Chin J Neuropsychiatric Disorders,2009,35(05):289-294.

[9]He YB, Liu YL, Yang ZD, Lu JH, Song Y, Guan YM, Chen YM. Effect of ginsenoside-Rg1 on experimental Parkinson's disease: A systematic review and meta-analysis of animal studies. Exp Ther Med. 2021 Jun;21(6):552.

[10]Qureshi AR, Jamal MK, Rahman E, Paul DA, Oghli YS, Mulaffer MT, Qureshi D, Danish MA, Rana AQ. Non-pharmacological therapies for pain management in Parkinson's disease: A systematic review. Acta Neurol Scand. 2021 Aug;144(2):115-131.

[11]Ki-Ho, Cho, Tae-Hun, et al. Moxibustion for idiopathic Parkinson's disease: A systematic review and meta-analysis of randomized controlled trials[J]. European Journal of Integrative Medicine, 2017.

[12] Zhang Xuechun, Jiang Li, Wu Yanan, Luo Ding, Fu Wenbin. Effect of moxibustion on Parkinson's disease based on Meta-analysis [J]. Asia-pacific Traditional Medicine,2016,12(14):96-100.

[13]Zhang G, Xiong N, Zhang Z, Liu L, Huang J, Yang J, Wu J, Lin Z, Wang T. Effectiveness of traditional Chinese medicine as an adjunct therapy for Parkinson's disease: a systematic review and meta-analysis. PLoS One. 2015 Mar 10;10(3):e0118498.

[14] Zhang Yi, Hu Siyuan, Li Xuan, CAI Lili. Efficacy and safety of ointment in the treatment of recurrent respiratory tract infection in children: a systematic review and Meta-analysis [J]. Chinese National and Folk Medicine,2023,32(6):98-103.

[15] Niu Liyun, He Jiani, Zhang Ding, Feng Weixing. Parkinson's disease pathogenesis and acupuncture treatment progress [J]. Journal of Liaoning University of Traditional Chinese Medicine, 2021,24(4):217-220.

[16] Wang Jiabin, Shen Xiaoming, Ma Yunzhi, Su Chaoyang, Bian Songbo. Research progress of Parkinson's disease in traditional Chinese and western medicine [J]. Chinese Journal of Experimental Formula Science, 222,28(1):241-250.

Summary of traditional Chinese medicine rehabilitation model of Parkinson's disease based on brain-gut axis theory [J]. Shandong Journal of Traditional Chinese Medicine, 2021,41(1):120-125.

[18] He Yingli, Liu Xin, Zou Donglei, Li Min, Zhuang Lixing. Analysis of the Etiology of Parkinson's disease myotonia in Traditional Chinese Medicine from the perspective of "clonus syndrome" and treatment based on meridian tendons [J]. Chinese Journal of Traditional Chinese Medicine, 2012,50(9):56-58.

[19] Li Ruiben, Luan Yibo, Guo Shengxuan, CAI Qiuhan, Hu Siyuan. Effect of placebo on tic disorders in children: a systematic review and Meta-analysis [J]. Drug Evaluation Research, 2021,45(4):759-767.

[20] Kong Xiangwei, Kang Simin, Shang Huifang et al. Effect of deep brain stimulation on depression in Parkinson's disease: a network meta-analysis [J]. West China Med,2023,38(03):416-423.

[21] Wu Ming-zhen, Luan Ji-xin, ZHANG Chuan-chen, et al. Diagnostic value of quantitative susceptibility mapping of substantia nigra in Parkinson's disease: a meta-analysis [J]. Magnetic resonance Imaging,2023,14(02):6-11.

[22] Li Ran, Liu Jiangang. Meta-analysis of the effects of traditional Chinese exercise on motor function and related indicators in patients with Parkinson's disease [J]. Shandong Sports Science and Technology, 2021,44(06):57-62.

[23] Hu Yibin, Zhang Lijuan, Ma Yizheng, et al. Meta-analysis of memantine versus donepezil in the treatment of Parkinson's disease dementia [J]. Drug Evaluation Research, 2021,45(12):2564-2573.

[24] Li Ai-ling, WANG Zhen-yu, LI Yong-jie et al. Meta-analysis of effects of deep brain stimulation and optimal drugs on motor ability and quality of life in Parkinson's disease [J]. South China Journal of Defense Medicine, 2021,36(11):908-915.

[25] He Yiting, Bai Yanan, He Liang. A meta-analysis on the relationship between lead, chromium, manganese and Parkinson's disease [J]. Safety and Environmental Engineering, 2021,29(05):36-45.

[26] CAO Meiqun, Feng Yifei, Jiang Ping, et al. Association between HLA-DRA rs3129882 polymorphism and Parkinson's disease: a Meta-analysis [J]. Chongqing Med, 2021,51(24):4252-4257.

[27] Huang Yue, Zhai Zhiyuan, Zheng Jinlong, et al. Olfactory disorder Parkinson's disease and cognitive function relationship of Meta analysis [J]. Journal of Chinese clinical research, 2022, 35 (8) : 1088-1094. The DOI: 10.13429 / j.carol carroll nki CJCR. 2022.08.012.

[28] Panpan Gan, Ling Liu, Yihong Quan. Meta-analysis of clinical efficacy and safety of traditional Chinese medicine combined with conventional western medicine in the treatment of Parkinson's disease with mild cognitive impairment [J]. Journal of Guangzhou University of Chinese Medicine, 22,39(08):1938-1946.

[29] Chen Zhuo, Wang Si-rui, Zhang Xin, et al. Systematic review and Meta-analysis of whole body vibration training for the treatment of motor symptoms in patients with Parkinson's disease [J]. Nerve injury and Functional Reconstruction, 2012,17(07):384-387+395.

[30] Chen Haosheng, Song Jiucun. Effect of Tai chi exercise on balance function in patients with moderate to mild Parkinson's disease: a Meta-analysis [J]. Chinese Journal of Sports Science and Technology Literature, 2020,30(05):128-133.

[31]Cheng I, Sasegbon A, Hamdy S. Dysphagia treatments in Parkinson's disease: A systematic review and meta-analysis. Neurogastroenterol Motil. 2022 Dec 22:e14517.

[32]Yang Y, Wang Y, Gao T, Reyila A, Liu J, Liu J, Han H. Effect of Physiotherapy Interventions on Motor Symptoms in People With Parkinson's Disease: A Systematic Review and Meta-Analysis. Biol Res Nurs. 2023 Apr 18:10998004231171587.

[33]Sun Y, Sheng J, Liu T, Yang G, He M, Huang Y, Luo Z, Zhu J, Jin X.Combination treatment of acupoint therapy and conventional medication for motor function of Parkinson's disease: A systematic review and meta-analysis.Complement Ther Clin Pract. 2023 Feb;50:101677.

[34]Wen X, Liu Z, Liu X, Peng Y, Liu H. The effects of physiotherapy treatments on dysphagia in Parkinson's disease: A systematic review of randomized controlled trials. Brain Res Bull. 2022 Oct 1;188:59-66.

[35]Niu Q, Xu W. Efficacy of Moxibustion in the Treatment of Parkinson's Disease Based on Meta-Analysis under Intelligent Medical Treatment. Appl Bionics Biomech. 2022 Apr 30;2022:8168152.

[36]Lei H, Ma Z, Tian K, Liu K, Wang J, Zhu X, Mi B, Chen Y, Yang Q, Jiang H.The effects of different types of Tai Chi exercises on motor function in patients with Parkinson's disease: A network meta-analysis. Front Aging Neurosci. 2022 Aug 29;14:936027.

[37]Wang K, Li K, Zhang P, Ge S, Wen X, Wu Z, Yao X, Jiao B, Sun P, Lv P, Lu L. Mind-Body Exercises for Non-motor Symptoms of Patients With Parkinson's Disease: A Systematic Review and Meta-Analysis. Front Aging Neurosci. 2021 Dec 3;13:770920.

[38]Wen X, Liu Z, Liu X, Peng Y, Liu H. The effects of physiotherapy treatments on dysphagia in Parkinson's disease: A systematic review of randomized controlled trials. Brain Res Bull. 2022 Oct 1;188:59-66.

[39]Lei H, Ma Z, Tian K, Liu K, Wang J, Zhu X, Mi B, Chen Y, Yang Q, Jiang H. The effects of different types of Tai Chi exercises on motor function in patients with Parkinson's disease: A network meta-analysis. Front Aging Neurosci. 2022 Aug 29;14:936027.

[40]Niu Q, Xu W. Efficacy of Moxibustion in the Treatment of Parkinson's Disease Based on Meta-Analysis under Intelligent Medical Treatment. Appl Bionics Biomech. 2022 Apr 30;2022:8168152.

**No-relevant: (491 articles)**

1. Fan W X, ZHANG J L, Xu C. Research progress on the role of α7 nicotinic acetylcholine receptors in central nervous system diseases [J]. Chin J Clin Pharmacology & Therapeutics,2021,26(09):1065-1072.
2. Wang Mingwei, Xie Ximei, Zhang Xulong, Bai Xiu, Shen Yating. Research on the mechanism of acupuncture in central nervous system diseases based on PI3K/Akt signaling pathway [J]. Clinical Journal of Acupuncture and Moxibustion,2021,37(09):98-101.
3. Wang G Q, Jin P, Yu J, Ma X F, Zhao R, Yang R M. Interpretation of complementary and alternative therapies for Parkinson's disease :2018 evidence-based clinical practice guidelines [J]. Journal of Anhui University of Traditional Chinese Medicine,2019,38(01):1-5.
4. Fang Zheng, Cao Xiaoyu, Gao Xiaoping. Efficacy of percutaneous tibial nerve stimulation in the treatment of neurogenic bladder in patients with central nervous system diseasesa Meta-analysis [J]. Abstracts of Recent World Medical Information (Continuous Electronic Journal),2020,20(19):25-29,32.
5. Klionsky DJ, Abdel-Aziz AK, Abdelfatah S, Abdellatif M, Abdoli A, Abel S, AbeliovichH, Abildgaard MH,Guidelines for the use and interpretation of assays for monitoring autophagy (4th edition)1. Autophagy. 2021 Jan;17(1):1-382.
6. Zhang YH, Hu HY, Xiong YC, Peng C, Hu L, Kong YZ, Wang YL, Guo JB, Bi S, LiTS, Ao LJ, Wang CH, Bai YL, Fang L, Ma C, Liao LR, Liu H, Zhu Y, Zhang ZJ, LiuCL, Fang GE, Wang XQ. Exercise for Neuropathic Pain: A Systematic Review and Expert Consensus. Front Med (Lausanne). 2021 Nov 24;8:756940.
7. Prasad EM, Hung SY. Current Therapies in Clinical Trials of Parkinson's Disease: A 2021 Update. Pharmaceuticals (Basel). 2021 Jul 25;14(8):717.
8. Xiao LY, Wang XR, Yang Y, Yang JW, Cao Y, Ma SM, Li TR, Liu CZ. Applicationsof Acupuncture Therapy in Modulating Plasticity of Central Nervous System. Neuromodulation. 2018 Dec;21(8):762-776.
9. Angelopoulou E, Anagnostouli M, Chrousos GP, Bougea A. Massage therapy as a complementary treatment for Parkinson's disease: A Systematic Literature Review. Complement Ther Med. 2020 Mar;49:102340.
10. Wang H, Liu Y, Zhao J, Guo X, Hu M, Chen Y. Possible inflammatory mechanisms and predictors of Parkinson's disease patients with fatigue (Brief Review). Clin Neurol Neurosurg. 2021 Sep;208:106844.
11. Wu C, Xu Y, Guo H, Tang C, Chen D, Zhu M. Effects of Aerobic Exercise and Mind-Body Exercise in Parkinson's Disease: A Mixed-Treatment Comparison Analysis. Front Aging Neurosci. 2021 Nov 18;13:739115.
12. Asakawa T, Fang H, Sugiyama K, Nozaki T, Kobayashi S, Hong Z, Suzuki K, Mori N, Yang Y, Hua F, Ding G, Wen G, Namba H, Xia Y. Human behavioral assessments in current research of Parkinson's disease. Neurosci Biobehav Rev. 2016 Sep;68:741-772.
13. Asakawa T, Fang H, Sugiyama K, Nozaki T, Hong Z, Yang Y, Hua F, Ding G, ChaoD, Fenoy AJ, Villarreal SJ, Onoe H, Suzuki K, Mori N, Namba H, Xia Y. Animal behavioral assessments in current research of Parkinson's disease. Neurosci Biobehav Rev. 2016 Jun;65:63-94.
14. Rabin ML, Stevens-Haas C, Havrilla E, Rosenstein A, Toffey B, Devi T, Earnhardt MC, Kurlan R. Complementary Therapies for Parkinson's Disease: What's Promoted, Rationale, Potential Risks and Benefits. Mov Disord Clin Pract. 2015 Jun 29;2(3):205-212.
15. Yang X, Zhou R, Di W, He Q, Huo Q. Clinical therapeutic effects of probiotics in patients with constipation associated with Parkinson disease: A protocol for systematic reviewand meta-analysis. Medicine (Baltimore). 2021 Nov 5;100(44):e27705.
16. Subramanian I. Complementary and Alternative Medicine and Exercise in Nonmotor Symptoms of Parkinson's Disease. Int Rev Neurobiol. 2017;134:1163-1188.
17. Lin TY, Hsieh CL. Clinical Applications of Bee Venom Acupoint Injection. Toxins (Basel). 2020 Sep 27;12(10):618.
18. Teixeira MZ, Guedes CH, Barreto PV, Martins MA. The placebo effect and homeopathy. Homeopathy. 2010 Apr;99(2):119-29.
19. Wang Z, Wan H, Li J, Zhang H, Tian M. Molecular imaging in traditional Chinese medicine therapy for neurological diseases. Biomed Res Int. 2013;2013:608430.
20. Jang S, Kim KH. Clinical Effectiveness and Adverse Events of Bee Venom Therapy: A Systematic Review of Randomized Controlled Trials. Toxins (Basel). 2020 Aug 29;12(9):558.
21. Lee H, Park HJ, Park J, Kim MJ, Hong M, Yang J, Choi S, Lee H. Acupuncture application for neurological disorders. Neurol Res. 2007;29 Suppl 1:S49-54. .
22. Chen L, Huang Y, Yu X, Lu J, Jia W, Song J, Liu L, Wang Y, Huang Y, Xie J, Li M. Corynoxine Protects Dopaminergic Neurons Through Inducing Autophagy and Diminishing Neuroinflammation in Rotenone-Induced Animal Models of Parkinson's Disease. Front Pharmacol. 2021 Apr 13;12:642900.
23. Peeraully T, Hameed S, Cheong PT, Pavanni R, Hussein K, Fook-Chong SM, Tan EK.Complementary therapies in hemifacial spasm and comparison with other movement disorders. Int J Clin Pract. 2013 Aug;67(8):801-6.
24. Jang JH, Park S, An J, Choi JD, Seol IC, Park G, Lee SH, Moon Y, Kang W, Jung ES, Cha JY, Kim CY, Kim S, Jung IC, Yoo H. Gait Disturbance Improvement and Cerebral Cortex Rearrangement by Acupuncture in Parkinson's Disease: A Pilot Assessor-Blinded,Randomized, Controlled, Parallel-Group Trial. Neurorehabil Neural Repair. 2020 Dec;34(12):1111-1123.
25. Yu Z, Zhang S, Wang D, Fan M, Gao F, Sun W, Li Z, Li S. The significance of uric acid in the diagnosis and treatment of Parkinson disease: An updated systemic review. Medicine (Baltimore). 2017 Nov;96(45):e8502.
26. Li L, Jin X, Cong W, Du T, Zhang W. Acupuncture in the Treatment of Parkinson's Disease with Sleep Disorders and Dose Response. Biomed Res Int. 2022 Feb 23;2022:7403627.
27. Jia J, Yu Y, Deng JH, Robinson N, Bovey M, Cui YH, Liu HR, Ding W, Wu HG, Wang XM. A review of Omics research in acupuncture: the relevance and future prospects for understanding the nature of meridians and acupoints. J Ethnopharmacol. 2012 Apr 10;140(3):594-603.
28. Fox S H , Katzenschlager R , Lim S Y , et al. The Movement Disorder Society Evidence-Based Medicine Review Update: Treatments for the non-motor symptoms of Parkinson's disease.[J]. Movement Disorders Official Journal of the Movement Disorder Society, 2011, 26(S3):S2-S41.
29. Dong Y, Hu Y, Sarkar S, Zong WX, Li M, Feng D, Song JX, Li M, Medina DL, Tan J, Zhang Z, Yue Z, Lu JH. Autophagy modulator scoring system: a user-friendly tool for quantitative analysis of methodological integrity of chemical autophagy modulator studies.Autophagy. 2020 Feb;16(2):195-202.
30. Zamotrinsky A , Afanasiev S , Karpov R S , et al. Effects of electrostimulation of thevagus afferent endings in patients with coronary artery disease.[J]. Coronary Artery Disease,1997, 8(8-9):551.
31. Yu YP, Ju WP, Li ZG, Wang DZ, Wang YC, Xie AM. Acupuncture inhibits oxidative stress and rotational behavior in 6-hydroxydopamine lesioned rat. Brain Res. 2010 Jun 8;1336:58-65.
32. Sreenivasmurthy SG, Iyaswamy A, Krishnamoorthi S, Senapati S, Malampati S, Zhu Z,Su CF, Liu J, Guan XJ, Tong BC, Cheung KH, Tan JQ, Lu JH, Durairajan SSK, Song JX, Li M. Protopine promotes the proteasomal degradation of pathological tau in Alzheimer's disease models via HDAC6 inhibition. Phytomedicine. 2022 Feb;96:153887.
33. Rui G, Guangjian Z, Yong W, Jie F, Yanchao C, Xi J, Fen L. High frequency electro-acupuncture enhances striatum DAT and D1 receptor expression, but decreases D2 receptorlevel in 6-OHDA lesioned rats. Behav Brain Res. 2013 Jan 15;237:263-9.
34. Kim S Y , Choe B Y , Lee H S , et al. Forelimb akinesia and metabolic alteration in the striatum following unilateral 6-hydroxydopamine lesion in rats: An in vivo proton magnetic resonance spectroscopy study[J]. Neurochemical Journal, 2011, 5(4):270-277.
35. Li Guoxang, Song Wenxin, Zhu Ling, Yu Qi, Pan Qiuxia, Li Jinghua, Wang Yinghui. Review and reflection on the development of meridian theory in traditional Chinese medicine [J]. International Journal of Traditional Chinese Medicine and Chinese Materia Medica,2023,45(3):257-261.
36. Li Baojin, Chen Jianpeng, Xu Runbing, Liang Jingyi, Shi Xiaoxi, Kong Weizheng, Ma Huifang, Li Rui, Liu Cunzhi. Overview of the literature theoretical research on extraordinary points [J]. Chin J Traditional Chinese Medicine,2023,38(3):1305-1312.
37. Lou Jincheng, MIAO Chanyun, Su Jiaqi, Hu Qing, Zhai Chuntao, Lu Yue. Establishment and evaluation of allergic asthma rat model [J]. Chin J Comparative Med,2023,33(1):130-137.
38. Liu Hao, Xu Hanying, Shi Zhong, Yao Fan, Tian Ziyue, LAN Tianye, Zhao Weimin. Association between ferroptosis and central nervous system diseases and progress of traditional Chinese medicine intervention [J]. Chinese Journal of Experimental Formula Science,2023,29(5):246-256.
39. Tang Han, Li Baoying, Lu Pu, Guo Wenhao, Lu Yonghui. Chinese Journal of Acupuncture and Moxibustion,2023,43(1):101-106+108.
40. Wang Qiming, Lu Yiping. Research progress of traditional Chinese medicine in the treatment of senile eczema [J]. Clinical Research of Traditional Chinese Medicine,2023,15(2):126-132.
41. Zhang Shenghong, Zhang Yanbin, Zhao Haiyin. Application of acupoint selection on time based on the rise and fall of qi and blood in insomnia [J]. Modern Distance Education of Chinese Traditional Medicine,2023,21(1):194-196.
42. Zhao Zhibin, Yuan Xuefi, Yan Hongli, Ju Shendan, Li Weihong, Liu Shimin. Application progress of traditional Chinese medicine in patients with PCOS undergoing in vitro fertilization-embryo transfer [J]. Journal of Liaoning University of Traditional Chinese Medicine,2023,25(1):43-47.
43. Huang Fan, Wu Zhennan, Qiu Mingwang, Gu Minqi, Fan Zhiyong, Wu Shan. Treatment of lumbar abdominal pain with massage: a case report and literature review [J]. Modern TCM Clinical,2023,30(1):113-116.
44. Wang Xianning, Zhang Xiaofeng, Cheng Xiaohong, Hu Xinghua, Lu Fenping, Gao Gao, Wang Xiaolin. Discussion on the idea of renal edema based on literature research [J]. Shaanxi Traditional Chinese Medicine,2023,44(2):220-223.
45. Chen Shuang, Yan Pengxuan, Gao Ning, Wang Weiming. Current situation and thinking on the differential treatment of chronic urinary retention with acupuncture and medication [J]. Chin J Traditional Chinese Medicine,2023,38(2):517-520.
46. Zhou Jingling, Liu Fei, Huang Xiaohui. Clinical observation of Wang Juyi meridian diagnosis method in the treatment of stable COPD [J]. Chinese Traditional Medicine Modern distance Education,2023,21(4):126-127+150.
47. Jiang Shangping, Fan Jingjing, Long Yan, Xiao Lingbo, Xie Sijian. Research status of external treatment of dry eye with traditional Chinese medicine [J]. Modern Distance Education of Chinese Traditional Medicine,2023,21(8):193-195.
48. Fan Wanqiong. Current status of traditional Chinese medicine in the treatment of gastroesophageal reflux disease [J]. Guangming Traditional Chinese Medicine,2023,38(1):197-200.
49. Chen Qin, Ji Wei. Research progress of traditional Chinese medicine in ankylosing spondylitis [J]. Clinical Medicine Research and Practice,2023,8(11):183-186.
50. Li Xiao-ying, Dong Xiao-qing, Ma Xiao-peng. Exosome and its research progress in the diagnosis and treatment of inflammatory bowel disease [J]. Modern Immunology,2023,43(1):60-66.
51. Su Ting, Lai Xin, Zhan Meiqi, Lin Jinglin. Progress in the treatment of bile reflux gastritis with traditional Chinese medicine [J]. Guangming Traditional Chinese Medicine,2023,38(5):999-1002.
52. Pang Lijian, Wang Pengqin, Lu Xiaodong, Shao Yan, Wang Linlin. Explanation and application of Peng's eye acupuncture therapy [J]. Chin J Traditional Chinese Medicine,2023,38(1):90-95.
53. Yang X, Luo Y K. Current status and future prospects of the treatment of chronic kidney disease-related skin pruritus [J]. Chinese Journal of Practical Clinical Medicine,2023,27(6):141-144+148.
54. Yuhui He, Mingxing Zhu, Yueyang Liu, Ruowei Zhao, Mingqing Huang, Hong Shi, Yanfang Zheng, Yanxiang Lin. Research progress on the mechanism of curcumin and resveratrol in the treatment of non-alcoholic fatty liver disease [J]. Chinese Medicine Review, 2020,20(1):57-60.
55. Tian Kaidi, Li Huili, Wang Huiwu. Research progress of traditional Chinese medicine on anterior ischemic optic neuropathy [J]. Modern Distance Education of Chinese Traditional Medicine,2023,21(7):206-206+I0001,I0002.
56. Wang Qian, Lin Yuehong, CAI Hong. Research status of traditional Chinese medicine in obese non-alcoholic fatty liver disease [J]. Medical Information,2023,36(1):181-184.
57. Wang Yu, Liu Ying. Clinical research progress of massage in the treatment of allergic rhinitis in children [J]. Guangming Traditional Chinese Medicine,2023,38(3):591-595.
58. Song Haikuo, Zhang Xuan, Du Kai, Nu Na, Li Guilan. Research progress of auricular point diagnosis and treatment technology [J]. China Urban and Rural Enterprise Health,2023,38(2):37-40.
59. Li Li, Li Hong, Zhang Bo, Sun Yizhen, Zhang Jian-guo, Wang Jie. Effect of acupoint application of traditional Chinese medicine combined with inhalation of tiotropium bromide on quality of life in patients with stable chronic obstructive pulmonary disease [J]. Chinese Primary Medicine,2023,30(2):172-176.
60. Wang Yaxin, Lin Yican, Guo Yinuo, Zhang Bo, Ma Yuying, He Xiaoping. Chinese Journal of Tissue Engineering,2023,27(13):2116-2123. (in Chinese)
61. Tang Yandan, Peng Yong, Nie Wei, Deng Xiang, Yang Shanshan. Current situation and thinking of traditional Chinese medicine in the treatment of multiple sclerosis [J]. World Traditional Chinese Medicine,2023,18(4):583-587+592.
62. WU Jia-wei, JIN Tao. Treatment progress of blepharospasm Meige syndrome [J]. Int J Ophthalmol,2023,23(3):421-424.
63. Mengyao Huang, Guozhong Chen, Rihui Zheng, Qi Tan, Li Zhong, Pengen Qin, Dewen Li, Zhangyu Xie. Research progress of common external treatment of traditional Chinese medicine in the treatment of severe acute pancreatitis [J]. Chinese Journal of Traditional Chinese Medicine Information,2023,30(2):178-180.
64. Qin Yining, Zhao Tianyi, Liu Fengbin, Wang Xin, Cao Xue, Sun Minglin, Lai Keyun, Di Luyao, Ge Zhishan, Liu Song, Xing Ying, Yang Lei, Yue Lihong, Zou Meimei, He Liyun, Li Hongjiao. Measurement characteristics of Chinese version of Gastrointestinal Symptom Rating Scale in patients with gastrointestinal diseases [J]. Chin J General Med,2023,26(18):2277-2285.
65. Li Yingcen, Chen Bo, Zhou Liang, Li Qiaonan, Chen Zhihan, Li Hongyang, Lu Fengda, Zhou Dan, Li Mingyue, Lu Zhongqian, Dou Baomin, Guo Yi. Development and promotion strategy of Guo 's hand-twelve well acupoint exercises [J]. Chin J Traditional Chinese Medicine,2023,38(3):1312-1316.
66. Liu Juncheng, Tan Chunlin, Ai Min, Li Zongao, Li Xigu, Zhong Qiping. Research progress of literature and treatment of knee osteoarthritis [J]. Guangming Traditional Chinese Medicine,2023,38(6):1197-1200.
67. Wang Li, Zhang Wei. Research progress on clinical application of Ciliao points [J]. Chinese Journal of Library and Information of Traditional Chinese Medicine,2023,47(1):104-107.
68. (in Chinese with English abstract) Progress in pathogenesis and traditional Chinese Medicine treatment of cerebral hemorrhage [J]. Journal of Liaoning University of Traditional Chinese Medicine,2023,25(1):103-106.
69. Li Q. Clinical research progress of traditional Chinese medicine rehabilitation technology in the treatment of post-stroke insomnia [J]. Guangming Traditional Chinese Medicine,2023,38(5):976-979.
70. Deng Wenwen, Du Junneng, Xiao Hui, Wang Shuhui. Clinical effect of Wang's round suppression massage in the treatment of neck pain of cervical spondylosis of neck type [J]. Journal of Guangzhou University of Chinese Medicine,2023,40(4):928-934.
71. Lu Yaoyao, Qian Yue, Chen Dexuan, Ma Chaoqun. Research progress on the differentiation and treatment of Hashimoto's thyroiditis by traditional Chinese Medicine [J]. Western Chinese Medicine,2023,36(1):153-156.
72. Haitao Jiang, Chenyu Fei, Rui CAI, Zhenlong Wang. Research progress of syndrome differentiation and treatment of children with ADHD [J]. Modern Distance Education of Chinese Traditional Medicine,2023,21(7):203-205.
73. Wang Chan, Wang Junhua, Zuo Wei, Xu Mingjun, Zhang Gang. Effects of intestinal microecological modulators on intestinal microbiome and immune microenvironment in patients with rheumatoid arthritis [J]. Journal of Hebei Medical University,2023,44(1):25-29+61.
74. CAI Zhihao, Xie Zhaoyong. Measurement and evaluation of femoral neck anteversion Angle: how to establish a unified method and standard [J]. Chinese Tissue Engineering Research,2023,27(9):1448-1454.
75. Liu Cui, DU Xiaozheng, Liu Limei. Research progress on the role of non-coding Rnas in exosomes in rheumatoid arthritis [J]. Chinese Journal of Pathophysiology,2023,39(2):359-365.
76. Wang Zitong, Hai Ying. Clinical research on the immediate effect of eye acupuncture therapy [J]. Massage and Rehabilitation Medicine,2023,14(4):67-70.
77. Wang Sijia Pan Jin, Zhao Yi, Tian Xuanhe, Ma Ming Han, HuiShan, Guo Dongjing, Song Xiaobin, Ma Ke. Current situation and prospect of treating bipolar disorder with TCM syndrome differentiation [J]. Journal of Chinese Medicine,2023,51(3):99-106.
78. Li Gehe, Cui Xiaoping, Yi Li, Zhang Juanjuan, Huang Mingzhu. Research progress of traditional Chinese medicine on premature ovarian failure [J]. Shaanxi Traditional Chinese Medicine,2023,44(4):534-537.
79. Fan Xiran, Li Duoduo, Wang Shuangshuang, Guo Ye, Yu Changhe. A qualitative study on the influencing factors of tuina manipulation in the treatment of musculoskeletal pain [J]. Chinese General Medicine,2023,26(2):219-224.
80. Peng Qinghua, Zhong Huiqiu, Lei Danhong, Luo Hongqiang. Individualized treatment of residual symptoms after repositioning maneuver in elderly patients with benign paroxysmal positional vertigo [J]. Journal of Jiangxi University of Traditional Chinese Medicine,2023,35(1):61-64.
81. Fu Qiao, Zhao Wenhao, Dai Hailong. Prevention, treatment and rehabilitation of COVID-19 with traditional Chinese medicine [J]. Journal of Practical Cardio-Cerebro-Pulmonary Vascular Disease,2023,31(1):8-11+21.
82. Li Fuxin, Du Xiaozheng, Zhang Xinghua, Zhang Fengfan, Chen Ping, Liu Qiang, Liu Limei, Zhang Xiauna. The role of HIF-1α in regulating glycolysis in the pathogenesis of rheumatoid arthritis [J]. Chinese Journal of Osteoporosis,2023,29(2):226-231.
83. Ma Yanmiao, Zhang Lihuan, Dang Qiong, Wei Yuyu, Qin Dynasty. Research progress of traditional Chinese and western medicine in the treatment of vulvar leukoplakia [J]. Chinese and Foreign Medical Research,2023,21(3):162-166.
84. He Zhanxue, Zhu Taifu, Li Xin, Su Xiaolan, Liu Huimin, Wang Lianchun. Differences in resistance and mechanism of different combinations of rootstock and scion to kiwifruit cankers [J]. Scientia Agricultura Sinica,2023,52(1):95-107.
85. Liang Hui Nie, Shuai Wang, Libin Yang, Rong Ma, Hanjiang Chen, Ping Rong Rong Rong. Research progress on genetic etiology, diagnosis and treatment of infantile spasms [J]. China Medicine Guide,2023,20(5):33-37.
86. Rui Ou, Song Wei, Zhihuang Chen, Xiaohao Li, Jun Liu, Xianxian Zhang, Yingwan Liu, Chunfu Hou, Shaowei Li. Clinical effect of Shuijin acupuncture on rheumatoid arthritis [J]. Chin J Traditional Chinese Medicine,2023,38(1):441-445.
87. Yang Wei, Wang Zidong, Jiang Nan, Li Zhen ©, Jiao Analysis and clinical application of Epigastric acupoints in Shangwan Zhongwan [J]. Guangming Traditional Chinese Medicine,2023,38(1):35-38.
88. Meng Sixuan, Yao Xingmei, Wu Xinye, Li Zhaodong, Wang Hao. Research progress on the mechanism of active ingredients of Astragalus membranaceus in the treatment of diabetic nephropathy [J]. Chinese Medicine Review,2023,20(4):60-63.
89. Chen Xiaojin, Liu Xiaodi, Wu Mingxin, Mou Ruiyu, Li Xiaojiang. Efficacy and safety of Xiaoyan decoction combined with camrelizumab and anlotinib hydrochloride in the treatment of advanced non-small cell lung cancer [J]. Chinese Journal of Traditional Chinese Medicine,2023,51(1):73-78.
90. Xu Dayong, Li Yunpeng, Wei Jingmei, Liu Ruyin. Mechanism of rutin ameliorating intervertebral disc degeneration in Rats [J]. Chinese Journal of Tissue Engineering Research,2023,27(14):2139-2145.
91. Cheng Shuo, Tan Yan, Ding Chengcheng, Chen Weihang, Peng Tiantian, Zhang Yali, Wang Yailei, Zhang Huawei, Li Haiyan, Zhang Jiani, Liu Zhaoheng, Wang Xu, Hua Qian. Research on the mechanism of Huanglian-Ejiao decoction in the treatment of depression based on network pharmacology and in vitro experiments [J]. Chinese Hospital Medication Evaluation and Analysis,2023,23(2):139-146.
92. Zhang Yan-fang. Application effect and influence of laparoscopic surgery and abdominal surgery in the treatment of uterine fibroids with diabetes mellitus [J]. Heilongjiang Med,2023,47(2):163-165.
93. Li H Y, Chen Y Q, Li Q L, Xie D Y, Chen R X (guidance). Chen R X's academic view and clinical application of "The essence of moxibustion, qi reaching and effective" [J]. Chinese Journal of TCM,2023,64(2):119-123.
94. Zefeng Zhao, Kaiwen Kang, Meng Nian, Qiang Wang, Haifa Qiao, Mingcheng Qian. Virtual screening and molecular dynamics study on the inhibitory components of superoxide dismutase 1 in Eu仲 [J]. Northwest J Pharmacy,2023,38(1):124-132. (in Chinese)
95. Wang X W, Wang X, Zhu T, Wang H D, Jiang H, Chen G Q, (Review). The role of NFATc 1 in the regulation of bone metabolism by microRNAs [J]. Journal of Southwest Medical University,2023,46(2):180-184+F0003.
96. [Tao J Y, Yuan X Y. Effect of montelukast sodium combined with Xiaoerkechuanling granule on mDCs and pDCs in peripheral blood of children with cough variant asthma and its curative effect [J]. Journal of Guizhou Medical University,2023,48(1):119-124.
97. Li Baoying, Tang Han, Guo Wenhao, Lu Pu, Lu Yonghui. Influence factors and diagnostic model of excessive residual urine volume in patients with benign prostatic hyperplasia [J]. Guangxi Medicine,2023,45(3):272-276.
98. Jie Jia, Huiyi Huang, Yanlong Li, Yixuan Cui, Jingwen Ding, Xueqing Chen. Application of superb microvascular imaging technology in syndrome differentiation of rheumatoid arthritis [J]. Imaging Research and Medical Application,2023,7(2):50-53.
99. Xu Xiangru, Zhang Wen, Zhang Xiangyu, Wu Xinxin, Sun Yuting, Yang Hongqiang, Pu Yuting, Zhou Shuang, Huang Xiaomin, Li Fang, Han Xiaotong, Shao Zhong, Fang Bangjiang. Efficacy and safety of Chaiqin Qingning capsule in the treatment of acute upper respiratory tract infection complicated with fever [J]. Shanghai Medicine,2023,44(1):25-29. (in Chinese)
100. Han Lin, Su Xiuzhen, Zhang Zhongyuan, Liu Ying, Wei Zhongxiang, Zhang Qinglan, Zhang Junping, Wang Chenxi, Su Quande. Effect of Jipilagium sp. on motor dysfunction in Parkinson's disease with hepatorenal insufficiency syndrome: a randomized controlled trial [J]. Chinese Acupuncture and Moxibustion, 2012,42(5):493-497.
101. Zheng Hesheng, Wu Yumei. Research progress on the mechanism of ferroptosis in the treatment of Parkinson's disease with traditional Chinese Medicine [J]. Chin J Traditional Chinese Medicine, 2021,40(10):20-27.
102. Yang Jiwei, Xiu Wencui, Gang Weijuan, Jing Xianghong. Chinese Journal of Acupuncture and Moxibustion, 2012,42(6):707-711.
103. Fan Jingqi, Lu Weijing, Wang Yuting, Yan Mingyue, Chen Yuanyuan, Li Yingjia, Tan Weiqiang, Wang Lili, Zhuang Lixing. A randomized controlled trial of governor vessel three-needle combined with hand-hand-clonic three-needle in the treatment of myotonia in early and middle stage of Parkinson's disease [J]. Chin J TCM, 2021,63(17):1662-1667.
104. Hu Xiyou, Chen Bo, Chen Ze-lin, Guo Yi, Chen Yong, Li Ling-cen, ZHANG Jingyu, Yang Ke-jian, XING Hao-Bo, Cen Yuan-yuan, Wang Li-fen, Guo Tao, Yang Chao-bo. Visual analysis of Cochrane systematic reviews of acupuncture and moxibustion in recent 10 years [J]. Tianjin Traditional Chinese Medicine, 2021,39(10):1241-1248.
105. Kelu Yang,Jiaoyan Zhang,Liang Zhao,Luying Cheng,Yuanyuan Li,Yuchen Kang,Xiangyu Zhang,Yingying Kang.An umbrella review of Lianhua Qingwen combined with Western medicine for the treatment of coronavirus disease 2019[J]. Acupuncture and Herbal Medicine (English), 2021,2(3):143-151.
106. Xu Guoqiang, Zhao Qiliang, Wang Xiaoyu, Zhang Dong, Fan Shuang, Zhang Huiqi, Wang Mutian, Chen Minghu, Liu Min. Reevaluation of the systematic review of Shufeng Jiedu capsule [J]. Chin J Materia Medica, 2012,47(4):1103-1113.
107. Qingyong Zheng,Ya Gao,Lu Xiong,Hengyi Huang,Junfen Li,Guoyuan OuYang,WulayinSaimire,Jingjing Yang,Yu Zhang,Xiaopeng Wang,Xiaofeng Luo.Chinese herbal medicine and COVID-19: quality evaluation of clinical guidelines and expert consensus and analysis of key recommendations[J]. Acupuncture and Herbal Medicine (English), 2021,2(3):152-161.
108. Feng Jingyi, Zhang Zhiwen, Lao Jinxiong. Clinical observation of warm acupuncture and moxibustion in the treatment of acute peripheral facial paralysis [J]. Chinese Journal of Traditional Chinese Medicine, 2012,50(9):80-83.
109. Zhao Hongyu, Xiong Feng, Liu Xinmin. Systematic review and Meta-analysis of Kangfu Xiaoyan suppository combined with antibiotics in the treatment of chronic pelvic inflammatory disease [J]. World Journal of Traditional Chinese Medicine, 2012,17(22):3180-3188+3199.
110. Zhang Zijing, Gao Feng. Clinical research progress of special acupuncture for hemifacial spasm in recent 5 years [J]. Chinese Materia Medica, 2012,11(2):189-195.
111. Chenfang Hu, Qiong Xu, Huanguan Wu, Jing Li, Zhaoqin Wang, Qin Qi, Rui Zhong, Huirong Liu, Kunshan Li, Luyi Wu. Research progress on the therapeutic mechanism of acupuncture and moxibustion in the treatment of non-amanitic mild cognitive impairment [J]. World Journal of Traditional Chinese Medicine, 2021,17(20):2959-2964.
112. Mengzhu Zhao, Yujia Zhang, Lv Cheng, Yan Li, Henghe Wang. Efficacy and safety of Shexiang Baoxin pills in the treatment of coronary heart disease: a systematic review [J]. Chin J Evidence-based Cardiovascular Med, 2021,14(2):137-142+156.
113. Li Li, Li Zao, Wang Yinghao, Huang Mei, Xiao Xiao, Fan Mengdi, Guo Chunyan, Yang Yue, Wang Zuhong. Clinical study on bee acupuncture and acupoint scattered needling combined with acupuncture for peripheral facial paralysis in recovery stage [J]. Journal of Clinical Acupuncture and Moxibustion, 2021,38(2):17-20.
114. Zhang Miao, Qu Yang, Liu Hongyu. Clinical effect of warm acupuncture and moxibustion in the treatment of chronic functional constipation [J]. Chinese Medicine Guide, 222,20(15):97-99.
115. Fan Xueming, Shen Wei, Wei Jing-jing, Fu Guo-jing, Liang Xiao, Gong Xiao, Chen Wen-jie, Park Jing-ze, Yan Yu, Guo Chun-li, Teng Jing, Zhang Hui, Zhang Yun-ling. Summary evaluation of clinical research on acupuncture for tension-type headache [J]. Chinese Journal of Traditional Chinese Medicine, 222,63(6):572-580.
116. Ma Zhongyi, Li Shujuan, Sha Yuping. Clinical research progress of classical acupuncture in the treatment of rheumatoid arthritis [J]. Progress in Clinical Medicine, 2021,12(8):7332-7337.
117. Zhang Li, Yuan Yi-yun, Wu Chang-le, Wang Qiang, Wang Yuan, Xing Wenwen, Qiao Hai-fa, Yang Xiao-hang, Liu Qi. The relationship between mitophagy and neurodegenerative diseases and the regulatory mechanism of acupuncture and moxibustion [J]. World Science and Technology: Chinese Medicine Modernization, 2021,24(8):3242-3248.
118. Li Yi, Li Shenghua, Zhou Mingwang, Chen Wei, Lu Guanjin, Yue Baichuan, Wang Pengzhi, Zhao Qiuyue. Research progress on analgesic mechanism of warm acupuncture in the treatment of knee osteoarthritis in early and middle stage [J]. Clinical Research of Traditional Chinese Medicine, 2021,14(24):108-111.
119. Riliang Su, Cui Li, Nan Yang, Xiuyang Li. Research progress of acupuncture and moxibustion in the treatment of polycystic ovary syndrome [J]. Henan Traditional Chinese Medicine, 2021,42(7):1114-1118.
120. Xie Xingguo, Dong Xu, Zhang Hongyan, Wang Ruoyu, Tong Dan, Wang Dongyan. Clinical study of repeated transcranial acupuncture at the motor area of head acupoints in the treatment of limb motor dysfunction in acute stage of stroke [J]. Clinical Journal of Acupuncture and Moxibustion, 2021,38(9):6-10.
121. Zhaochen Ji,Haiyin Hu,Danlei Wang,Marco Di Nitto,Alice Josephine Fauci,Masayoshi Okada,Kai Li,Hui Wang.Traditional Chinese medicine for promoting mental health of patients with COVID-19:a scoping review[J]. Acupuncture and Herbal Medicine (English), 2021,2(3):184-195.
122. Liang Hui, Long Lideng. Research progress on the application of brain-gut axis theory in acupuncture and moxibustion [J]. Clinical Research of Traditional Chinese Medicine, 2012,14(36):92-96.
123. Zhang Jie, Zhang Xuezhu. Xingnao Kaiqiao acupuncture improves endothelial function in patients with cerebral small vessel disease by regulating FGF23-αKlotho pathway [J]. Tianjin Traditional Chinese Medicine, 2021,39(1):53-57.
124. MA Q L, HU B, WANG P, LI Y H, Yang F. A preliminary study on the systematic thought of acupuncture and moxibustion in the Jingui Yaolue [J]. Modern distance education of traditional Chinese Medicine in China, 2020,20(10):65-68.
125. Du Ting, Wang Jing. Correlation analysis between magnetic resonance measurement parameters of infrapatellar fat pad and clinical efficacy of warm acupuncture in the treatment of knee osteoarthritis [J]. Shanxi Journal of Medicine, 2021,51(17):1939-1943.
126. Wang Yi-I, Li Bin, Wang Lin-peng, Jing Xiao-hong, Huang Yi, Hu Hui, Liu Zhi-shun, FU Yuan-bo, Liu Qingquan. Chinese Journal of Acupuncture and Moxibustion, 2021,42(6):634-638.
127. Chen Xuekai, Liang Hui. Research progress of acupuncture and exercise therapy in the treatment of pain [J]. Clinical Research of Traditional Chinese Medicine, 222,14(36):97-100.
128. He Qiuxia, Du Yuzheng, Meng Xianggang, Shi Xuemin. Real world study on the effect of Huoxue Sanfeng acupuncture on blood pressure in patients with cerebral infarction complicated with hypertension [J]. Chin J General Med, 2021,25(5):577-583.
129. Wang H, Xu W H. Observation on the effect of warm acupuncture combined with focused ultrasound Fu talin latex introduction on sports injury [J]. Journal of Nanjing University of Physical Education, 22,21(3):44-47.
130. Li Ran, He Jiakai, Jiang Yuhang, Jia Baohui. Experimental research progress of acupuncture intervention for Alzheimer's disease based on SAMP8 mice [J]. Acupuncture Research, 22,47(5):466-470.
131. Zhao Xuchun, Zhao Yaodong, Gao Pengpeng, Yue Na, Meng Lijuan, Fang Xiaoli. Clinical research overview of acupuncture in the treatment of tinnitus and deafness [J]. Journal of Practical Traditional Chinese Medicine Internal Medicine, 2021,36(5):43-45.
132. Liu ANI. Effect of traditional Chinese medicine constitution identification nursing intervention combined with warm acupuncture and moxibustion on patients with chronic obstructive pulmonary disease and the prevention of pneumonia [J]. Capital Food & Medicine, 2012,29(8):121-123.
133. Yang Yuanyuan, Zhou Haichun, Wang Di, Cheng Weiping. Clinical study on Huayu Ditan Xingnao Acupuncture in the treatment of chronic cerebral artery insufficiency with mutual accumulation of phlegm and blood stasis [J]. Chinese Medicine Information, 2021,39(1):40-45.
134. Gao Pan, Hu Hong-da. Effect of warming acupuncture combined with Buzhong Yiqi decoction on 45 cases of stomach pain of spleen-stomach deficiency and Cold and its influence on TCM syndromes [J]. Drug Evaluation, 2012,19(9):561-563.
135. Huang Jufang, Luo Ting, Luo Weisheng. Research progress on the mechanism of acupuncture in the treatment of ischemic stroke [J]. Acupuncture Research, 222,47(1):78-82.
136. He Meiqin, Li Zhiyong, Liang Fengming, Li Qing. Research progress of acupuncture in the treatment of glaucoma [J]. China Journal of Traditional Chinese Medicine Ophthalmol, 2021,32(1):66-69.
137. Fan Su, room Yi purge. Research progress of acupuncture regulating different signal pathways to improve ovarian reserve function [J]. Acupuncture Research, 222,47(7):644-648.
138. JIN C, WU Y, QIN Y X, LI X N. Clinical research progress of the "nine needling" method in the "Official Acupuncture of Lingshu" [J]. Clinical Journal of Acupuncture and Moxibustion, 2021,38(2):83-87.
139. Jin Xianghui, Wang Fuchun. Research overview of acupuncture in the treatment of dysmenorrhea [J]. Chinese Medicine Information, 2012,39(8):80-84.
140. Yu Ziwei, Lu Mengjiang, Yu Zhi, Xu Bin. To explore the mechanism and optimization of therapeutic effect of acupuncture on obesity from sympathetic nervous system [J]. Acupuncture Research, 222,47(8):744-748.
141. Wang Qi, Shao Jing, Liu Mingwei, Shi Huaying. Effect of herbal tea drinking combined with Back-shu point warming acupuncture on blood flow and blood lipid in patients with coronary heart disease complicated with hyperlipidemia [J]. Chin J of Rare Diseases, 2020,29(2):99-102.
142. Wen Da, Li Chunmei. Overview of clinical research on acupuncture of sphenopalatine ganglion [J]. Chinese Journal of Acupuncture and Moxibustion Electronic Journal, 2021,11(3):113-115.
143. Wang Yue, Wang Liping, Wang Yahui, Chen Chen, Yu Xin, Wu Linlu, Zou Yihuai. Hotspots and trends of magnetic resonance research of acupuncture based on CiteSpace visualization [J]. Chinese Medicine Review, 2012,19(32):108-111+121.
144. Song Kai, Wang Yating, Xiong Fan-jie, Huang Ai-ling, Zhang Hong. Analysis of the current situation and rules of overseas acupuncture and moxibustion mechanism research in the past ten years [J]. China Journal of Basic Medicine of Traditional Chinese Medicine, 2021,28(5):780-784+795.
145. Chen Jialian, Zhao Zhongting, Zhu Tiantian, Yang Dongyu. Clinical application of acupuncture and moxibustion in the treatment of rheumatoid arthritis by rehabilitation assessment technique [J]. Journal of Practical Traditional Chinese Medicine Internal Medicine, 2021,36(11):24-27.
146. Tao Bo, Zeng You, Xie Qiang. Research progress on acupotom-needle-knife therapy as a treatment for pharyngeal diseases in recent five years [J]. Journal of Jiangxi University of Traditional Chinese Medicine, 2021,34(5):115-117+124.
147. Chen Chunhai, Cong Qianhe, Liu Chunyu, Wang Zhiying, Gong Haiping. Xu Guangli's experience in the treatment of low back pain with acupuncture [J]. Journal of Changchun University of Traditional Chinese Medicine, 2021,38(6):614-616.
148. Ke Chao, Cao Yang, Xia Yewan, Fang Chuang, Shi Senjie, Shi Wenying, Pan Jiang, Zhang Wei. Brief analysis of the research progress of different acupuncture methods in the treatment of Alzheimer's disease [J]. Journal of Hunan University of Chinese Medicine, 222,42(2):337-342.
149. Li Yijun, Liang Xingsen, Wang Huajun, Gao Yanping, Li Ling. Research progress of acupuncture and moxibustion in the treatment of osteoarthritis [J]. Chinese Journal of Acupuncture and Moxibustion Electronic Journal, 2021,11(4):146-148.
150. Jiang Xiaoli, Wang Shengchun. Clinical research progress of acupuncture in the treatment of post-stroke motor aphasia [J]. International Journal of Traditional Chinese Medicine and Chinese Materia Medica, 2021,44(7):830-833.
151. Ji Huijie, Sun Xinyi, Shi Lixin, Gao Weiping. Research progress on the types of needles and acupuncture techniques in the treatment of dry eye [J]. China Journal of Traditional Chinese Medicine Ophthalmology, 2021,32(10):823-826.
152. Li Huimei, Qin Xiaoguang, Sun Yuyu, Zhang Yi, Gao Yan, Fan Jing. Clinical research progress of Zheng's acupuncture in the treatment of eye diseases in the past 15 years [J]. Clinical Research of Traditional Chinese Medicine, 222,14(9):6-9.
153. Wang Xina, Gao Ling, Liu Congying, Gao Xiyan. Chinese Journal of Traditional Chinese Medicine, 2012,37(1):214-217.
154. Huang Fen, Fu Jiajun, Yan Juan, Yuan Xian, Huang Gang. Clinical research progress of acupuncture in the treatment of chloasma [J]. Journal of Jiangxi University of Traditional Chinese Medicine, 2021,34(6):112-115.
155. Cao Yuchun, Zu Yang, Li Baijun, Zheng Jie. Application of surface electromyography in acupuncture research [J]. Shanghai Journal of Traditional Chinese Medicine, 2021,56(10):95-98.
156. Gao Yan, Qin Xiaoguang. Summary of acupoint selection in the treatment of glaucoma with acupuncture [J]. Guangming Traditional Chinese Medicine, 222,37(15):2785-2787.
157. Wang Songtao, Li Yanwei, Zhao Yadan, Wang Jiaqi, Lu Shanshan, Li Wei, Tang Hui-ling, Shang Xiu-kui, Guo Yi, Xu Zhi-fang. Effect of acupuncture on cardiovascular disease and its central autonomic nervous mechanism [J]. Shandong Journal of Traditional Chinese Medicine, 2021,41(7):795-800.
158. Lin He, Ting Guo, Binye Zhu, Xue Li, Bo Ren, Hui Li, Lei Xu, Qiang Wang, Yuan Wang, Wensheng Wang. Study on the mechanism of acupuncture in the treatment of vascular dementia [J]. Journal of Liaoning University of Traditional Chinese Medicine, 2021,24(6):201-204.
159. Peng Kou, Yonghong Lei, Zhigao Sun, Chen Shan. Application progress of acupuncture and moxibustion in military training-related psoas muscle injury [J]. Journal of Changchun University of Traditional Chinese Medicine, 2021,38(4):455-460.
160. Ji Huijie, Sun Xinyi, Gao Weiping. Research progress of acupoint selection rules and syndrome differentiation in the treatment of dry eye with acupuncture [J]. China Journal of Traditional Chinese Medicine Ophthalmology, 2021,32(12):978-981.
161. Liu Yong, Yu Heng. Clinical effect of electroacupuncture at Yishen acupoints for Alzheimer's disease with sea of marrow deficiency [J]. China Medicine Review, 2012,19(4):136-139.
162. Meng Chao, Tu Ya, Li Jun, Jiao Qian, Tan Yuqing, Jiang Huili. Research hotspots and trends of acupuncture antidepressant based on CiteSpace visualization [J]. Chinese Medicine Review, 22,28(5):138-146.
163. Liu Cuili, Zuo Tao. Research progress of acupuncture in the treatment of glaucoma optic atrophy [J]. Massage and Rehabilitation Medicine, 2012,13(10):77-80.
164. Chen Hongyu, Shao Yan, Wang Pengqin. Clinical study on acupuncture treatment of spastic hemiplegia after stroke [J]. Chinese Journal of Clinical Medicine, 2021,34(7):1362-1367.
165. Wen Qiao, LAN Lei, Liu Yalan, Xiao Qingqing, Zeng Fang. Analysis of the research status of acupuncture mechanism based on functional magnetic resonance imaging [J]. Chin J Traditional Chinese Medicine, 2021,37(9):5463-5466.
166. Zhang Q, ZHU C, ZHANG X, HU R, WEN J, Liu K, SHEN H. Treatment of 23 athletes with knee tendon around the insertion point by "acupuncture at the diseased site" [J]. Chinese Journal of Orthopedics and Traumatology, 2021,30(10):48-51.
167. Li Pengcheng, Wang Fei, Hu Yucai. Effect of self-made Wubi decoction combined with warming acupuncture on stable angina pectoris of coronary heart disease [J]. Chin J Practical Med, 222,49(24):116-119.
168. Li Lei, Su Li. Exploration on the acupoint probing and needling Method of Canggui [J]. Modern Distance Education of Chinese Traditional Medicine, 2012,20(22):120-122.
169. Rongjia Xu, Zellu Xia, Yishan Qin, Fangzhou Jin, Chen Jiang. Meta-analysis of clinical efficacy and safety of external treatment of traditional Chinese medicine in the treatment of hyperthyroidism and hyperthyroid exophthalmos [J]. Journal of Zunyi Medical University, 2021,45(2):231-241.
170. Ma Shuai, Han Meng, Yang Dianhui, Ma Zubin. Clinical research status of acupuncture and moxibustion in the treatment of benign prostatic hyperplasia [J]. International Journal of Traditional Chinese Medicine and Chinese Materia Medica, 222,44(12):1455-1459.
171. Zhou Yuting, Yang Yaozhong (guidance), Shen Teli. Clinical research progress of acupuncture in the treatment of diabetic peripheral neuropathy [J]. Guangming Traditional Chinese Medicine, 222,37(1):118-121.
172. Fan Yizi, Zhang Zhen. Research progress of different acupuncture methods in the treatment of postoperative pain of anorectal disease [J]. Chinese National and Folk Medicine, 2021,31(18):57-62.
173. Huang Xiarong, Peng Ting, Zhou Jun. Research progress of acupuncture in the treatment of knee osteoarthritis [J]. Massage and Rehabilitation Medicine, 2012,13(9):68-71+76.
174. Luo Rong, Shi Wenying, Ye Yong, Bao Chengtong, Fan Sheng, Zhang Wei. Evidence map and association rule analysis of acupuncture for lumbar spinal stenosis [J]. World Journal of Traditional Chinese Medicine, 2021,17(7):988-992+997.
175. Li Ying-xiao, Zhang Kai-bo, Zhang Lu-peng, Li Xian. Research progress of acupuncture in the treatment of gastroesophageal reflux disease [J]. Shaanxi Traditional Chinese Medicine, 2021,43(8):1147-1149.
176. Xu Miao, Chen Fuyan, Zhang Xiangxiang, Wang Fang, Chen Kaiyi, Han Lin, Wu Bangqi. Research progress of acupuncture and syndrome differentiation in the treatment of diarrhea-predominant irritable bowel syndrome [J]. Information of Traditional Chinese Medicine, 222,39(4):81-84.
177. Li Guiyuan, Tong Juan, He Ying, Su Junlong, Liu Changsheng, Chen Jianxiong. Evaluation and analysis of short-term practice effect of acupuncture in Hong Kong university students [J]. Modern Distance Education of Chinese Traditional Medicine, 2021,20(15):152-154.
178. Wang Yi-xiang, Kang Xiao-na, RAN Bin-yan, Chen Yi-ze, SUN Zhao, Shen Wei. Research progress of functional magnetic resonance mechanism of acupuncture in the treatment of generalized anxiety disorder [J]. Guide to Traditional Chinese Medicine, 222,28(12):227-232.
179. Zheng Luo, Zhichao Guo, Xiaolin Lv, Ji Liang, Xinyuan Cao, Xing Wang, Hongna Yin. To explore the role of acupuncture in central nervous system diseases based on NLRP3 mediated pyroptosis pathway [J]. Clinical Journal of Acupuncture and Moxibustion, 2021,38(12):89-94.
180. Ma Yao, Bu He, Chen Qingwei, Yan Huiming, Zhang Xue, Lu Hongyan, Wang Zixuan. Effect of acupuncture combined with governor vessel moxibustion on ankylosing spondylitis in early and middle stage and its effect on bone marrow edema of sacroiliac joint [J]. Chinese Acupuncture and Moxibustion, 2012,42(9):971-976.
181. Zhang Bo, Yang Tiansong, Li Meng, Sun Xiaowei, Feng Chuwen, Sun Zhongren, Zhou Xiaoqing. Research progress on the mechanism of acupuncture regulating multiple types of autophagy in Alzheimer's disease [J]. Journal of Clinical Acupuncture and Moxibustion, 2021,38(3):97-101.
182. Zhao Ni, Li Jing. Research progress of acupuncture in the treatment of hemifacial edema after stroke [J]. Guangming Traditional Chinese Medicine, 2021,37(23):4406-4406+F0003,F0004.
183. Zhang Wenyu, Li Yibing, Li Lixian, Han Lin. Research progress of angiogenesis and acupuncture intervention after ischemic stroke [J]. Chinese Journal of Traditional Chinese Medicine Information, 222,29(4):137-144.
184. Lin Zhongsi, Yu Dongsong, Zhao Jinlong, Shi Huiyang, Zhang Zhuqiang, Zhao Lei, Ju Pin. Chinese Journal of Acupuncture and Moxibustion, 2012,42(12):1379-1383.
185. Yuan Hang, Yu Xiaohua, Li Xiang, Qin Sijun, Liang Guixiang, Bai Tianyu, Wei Benzheng. Analysis of spontaneous brain activity and functional connectivity in resting state after acupuncture at the uterine point [J]. Digital Chinese Medicine (English), 2021,5(1):59-67.
186. Wei Yuting, Zhu Tiantian, Jia Jing, Yan Xingke. Research progress on the mechanism of acupuncture and moxibustion in the intervention of Alzheimer's disease [J]. Acupuncture Research, 222,47(4):362-368.
187. Han Huan, Wang Kangfeng, Li Mingxiang, Guan Xiuju, Zhang Lijuan. Research progress of acupuncture regulating GSK-3β in the treatment of Alzheimer's disease [J]. Journal of Clinical Acupuncture and Moxibustion, 2021,38(9):86-91.
188. Sun Jifei, Wang Zhi, Chen Limei, Guo Chunlei, He Jiakai, Gao Deqiang, Fang Jiliang. Clinical research progress of acupuncture and moxibustion in the treatment of insomnia [J]. World Journal of Traditional Chinese Medicine, 2012,17(10):1475-1480.
189. Zhong Pei-ling, Liu Lin-hua, HE Jin-song. Research progress of acupuncture and moxibustion in the treatment of non-alcoholic fatty liver disease [J]. Journal of Guangzhou University of Chinese Medicine, 2021,39(3):727-734.
190. Yin Tao, Teng Yuke, Zeng Fang. Current status and prospect of neuroimaging research on acupuncture and moxibustion [J]. Life Science, 2021,34(5):517-524.
191. Liu Shiwei, Huang Xiaojin, Guo Yaoguang. Clinical study on the treatment of tinnitus of liver qi stagnation type with Shugan Lidan acupuncture [J]. Clinical Research of Traditional Chinese Medicine, 2012,14(22):73-76.
192. Su Nan, Zhang Kai. Research progress of acupuncture and moxibustion in the treatment of diarrhea-predominant irritable bowel syndrome [J]. China Urban and Rural Enterprise Health, 2021,37(6):79-82.
193. Wang Zelan, Bart, Meng Zhihong, Zhao Xiaofeng. Effect and research progress of acupuncture on collateral circulation in acute ischemic stroke [J]. Journal of Clinical Acupuncture and Moxibustion, 2021,38(5):98-101.
194. Wu Yue, Cao Danna. Research progress of functional magnetic resonance imaging on the brain central mechanism of acupuncture for insomnia [J]. Clinical Research of Traditional Chinese Medicine, 2012,14(34):86-89.
195. Jinqiu, Silunge, Iragu, Xiaohong, Batunashun, Alus, Bao Aruhan, Agula. Clinical observation on 32 cases of sciatica with lumbar disc herniation treated with warm needle of Mongolian medicine [J]. Chinese National and Folk Medicine, 2021,31(14):105-109.
196. Sun Haojie, Wang Zhou, Xia Changjun. Rules of acupoint selection in the treatment of postoperative gastroparesis syndrome with acupuncture [J]. China Medicine Guide, 222,19(21):17-20+35.
197. Wang LAN, Chen Zhili, Xu Liang, Xu Xiaoming. Effect of scalp acupuncture combined with comprehensive rehabilitation training on cognitive function in patients with early Alzheimer's disease [J]. Liaoning Journal of Traditional Chinese Medicine, 222,49(3):178-181.
198. Yu Huiling, Zheng Danyu, Zhou Haiyan. Application of classical acupuncture in the treatment of knee osteoarthritis [J]. Asia-pacific Traditional Medicine, 2012,18(10):220-224.
199. Qi Yulin, Jia Qianyu, Ye Hejiang. Research progress of acupuncture in the treatment of retinitis pigmentosa [J]. Chin J Traditional Chinese Medicine, 2021,37(4):2148-2151.
200. Zhou Lijun, Mu Yanli, Wu Meng. Clinical effect of Tongyuan governor moxibustion combined with acupuncture on cervical spondylosis [J]. Chinese Convalescence Medicine, 2021,31(5):528-531.
201. Zeng Liang, Sheng Li, Yang Shan-ying, Yao Jia, Zeng Juan-ni. Meta-analysis of the effect of auricular bean-pressing on postoperative analgesia in anorectal disease [J]. Chinese Journal of Library and Information of Traditional Chinese Medicine, 2012,46(2):22-28.
202. Hu Bingyu, Xu Tao, Zhao Ling. Research hotspots and trends of acupuncture and moxibustion in the treatment of functional dyspepsia in the past decade: based on literature keyword cluster analysis [J]. Chin J TCM, 2021,63(14):1327-1332.
203. The mechanism and clinical research progress of acupuncture and moxibustion in the treatment of depression and insomnia comorbidity in recent years [J]. Asia-pacific Traditional Medicine, 2012,18(8):219-222.
204. Cui Ying, Ma Lihong, Dong Xu. Comparison and research progress of common filiform needle acupuncture and Western "dry needle therapy" for pain treatment [J]. Shandong Journal of Traditional Chinese Medicine, 2021,41(2):239-244.
205. Zhang Ying. Clinical study on acupuncture combined with Fangfeng decoction in children with febrile convulsion [J]. Wisdom Health, 2021,8(15):94-96.
206. Tan Qiarui, Wang Yu, Zhao Xiaofeng. Research progress of brain functional magnetic resonance imaging in Taichong acupuncture [J]. Chinese Rehabilitation Medicine, 2021,31(6):573-576.
207. Wang Feng, Hu Saiqin, Li Sha, Lv Jing, Zhang Yi, Lin Nan, Qiao Yingbo, Peng Cailiang. Research progress on the central effect mechanism of acupuncture in the treatment of mild cognitive impairment based on rs-fMRI [J]. Liaoning Journal of Traditional Chinese Medicine, 222,49(9):214-217.
208. Xu Zhou, Yin Jichao, Hu Xinglv, Cheng Yuan, Wang Leilei, Wang Zhili. Research progress on molecular biological mechanism of acupuncture in the treatment of skeletal muscle injury [J]. Journal of Liaoning University of Traditional Chinese Medicine, 2021,24(4):192-196.
209. Zhang Hui, Zhang Xuemei, Zhang Fengzheng, Jiang Hua. Effect of acupuncture combined with temporal three-needle on postpartum hypogalactia migraine based on the theory of "nourishing the liver and strengthening the spleen" [J]. Western Chinese Medicine, 2012,35(11):128-131.
210. Ma Jing, Ye Gaxi. Clinical effect of stroke rehabilitation capsule combined with acupuncture in the treatment of peripheral facial paralysis [J]. Progress in Clinical Medicine, 2012,12(11):9789-9795.
211. Zhong K, Huang Q, Fang Y X, Ni Q Y, HE Z Y, Yang B. Meta-analysis of the diagnostic efficacy of miRNA-21 for diabetic nephropathy [J]. Chinese Journal of Medical Research, 2021,51(7):47-52.
212. Wang Yiting, Fang Yongjiang, Luan Sha, Guo Chunyan, Sun Junjie, Li Shaorong. Research progress of acupuncture and moxibustion in the treatment of knee osteoarthritis [J]. Chin J Traditional Chinese Medicine, 2021,37(6):3356-3358.
213. Junyi Long, Zhaoqin Wang, Zheng Shi, Mu 'en Gu, Yan Huang, Huan-gan Wu. Research progress of acupuncture and moxibustion in the treatment of adverse reactions of tumor chemotherapy [J]. World Journal of Traditional Chinese Medicine, 2021,17(10):1470-1474+1480.
214. Li Xiao-ning, Jin Cheng, Chen Fu-you, Li Rui, Liu Liangyu, Wang Sen, Qin Ye-xiao. Visualization analysis of acupuncture and moxibustion for bulbar paralysis based on CiteSpace [J]. Journal of Clinical Acupuncture and Moxibustion, 2021,38(1):47-53.
215. Kang Li. Research progress of acupuncture and moxibustion in the treatment of migraine [J]. Chinese National and Folk Medicine, 2021,31(21):53-56.
216. Lu Xianqiu, Dai Linglin, Chen Lifang, Zhai Dong. Clinical effect of acupuncture combined with acupoint application in the treatment of breast hyperplasia [J]. Journal of Zhejiang Chinese Medical University, 222,46(11):1278-1282.
217. Liu Qianli, Tang Yizhou, Shi Shuai, Liu Shuang, Han Mengmeng, Liu Ya. Effect of massage on constipation after stroke: a Meta-analysis [J]. Chin J General Med, 2021,20(9):1593-1597.
218. Wu Rong, Wang Fang, Wang Han. Meta-analysis of heat-sensitive moxibustion in the adjuvant treatment of chronic pelvic inflammatory disease [J]. Chinese Folk Therapy, 2012,30(22):37-40.
219. Wu Chaochun, Huang Zhengde, Pang Yu, Wei Xiaoying, Li Wei. Effect of health management on hyperuricemia population: a Meta-analysis [J]. Health Physical Examination and Management, 2021,3(1):40-45.
220. Niu Ye, Wei Lijuan, Wang Fuchun. Research progress of protein molecules related to acupuncture treatment of amblyopia [J]. China Journal of Traditional Chinese Medicine Ophthalmology, 2021,32(3):237-241.
221. Liu Jin-Zhan. Application of acupuncture and moxibustion in the treatment of ischemic stroke [J]. Medical Information, 2021,35(4):65-67.
222. Hu Runan, Zhang Chu, Song Yufan, Zhang Mingmin, Huang Guangying, Dong Haoxu. Review on the clinical application of acupuncture and moxibustion in different patient groups in the field of assisted reproduction [J]. Clinical Journal of Acupuncture and Moxibustion, 2021,38(12):83-88.
223. Ding Hui, Tang Wei, Li Mengxing. Acupuncture and moxibustion for rehabilitation of dysphagia after stroke [J]. Journal of Anhui University of Traditional Chinese Medicine, 2021,41(2):30-34.
224. Ma Qiaolin, Yang Fan, Hu Bin, Li Yanhui, Wang Xinkao. Real world study on systematic rehabilitation program of sleep disorders after stroke [J]. Clinical Research of Traditional Chinese Medicine, 2012,14(23):108-112.
225. Zhang Dongxue, Zou Wei. Research progress on the mechanism of acupuncture inhibiting neurovascular unit injury after stroke [J]. Chinese Medicine Information, 222,39(3):79-84.
226. Miao-Miao Hong, Encong Zhao, Limin Chen, Feng Wang, Wanqing Guo, Xue-hua Zheng, LAN Lin, Chang-Zheng Li, Wei-guo Dong. The mechanism of electroacupuncture on complement and phagocytosis of microglia in hippocampus of SAMP8 mice [J]. Acupuncture Research, 222,47(6):479-484.
227. (Kong Fandan, Zhang Wei. Research progress of acupuncture and moxibustion in the treatment of migraine [J]. Chinese National and Folk Medicine, 2021,31(23):64-66.
228. Zhong Li people, zhang school Yun, yuki. Effect of Tongmai Huoluo Qutan decoction combined with scalp acupuncture and body acupuncture on hemiplegia after stroke [J]. Chinese and Foreign Medical Research, 2012,20(22):137-140.
229. Gao Ya, Liu Guiying. Nursing experience of intradermal acupuncture for a patient with insomnia after stroke [J]. Nursing of Integrated Traditional Chinese and Western Medicine, 222,8(8):96-99.
230. Songjiang Zhang, Jianfeng Gao, Ningning Sun, Longyang Li, Yanhui Li. Effect of electroacupuncture on the proliferation of endogenous neural stem cells in the hippocampus of young rats with Alzheimer's disease [J]. Chinese Acupuncture and Moxibustion, 2012,42(2):167-172.
231. Li Hui-hui, Wang Ying, Ji Hai-sheng, Liu Xiu-xiu, Han Wei. Effect of Qinglong wagging tail acupuncture in the treatment of shoulder-hand syndrome after stroke [J]. Clinical Journal of Traditional Chinese Medicine, 2021,34(1):159-163.
232. Zhang You, Yin Yalong, Wu Xingui. Research progress on the recovery of neurological function after cerebral infarction based on electroacupuncture therapy [J]. International Journal of Neurology and Neurosurgery, 2021,49(1):79-86.
233. Hua H Y. Clinical observation of acupuncture combined with Shenlingbaizhu powder in the treatment of ulcerative colitis with spleen deficiency and damp-heat [J]. Journal of Guangzhou University of Chinese Medicine, 2021,39(3):586-593.
234. Zhang Dan, Wang Enlong. Research progress of acupuncture combined with neuromuscular electrical stimulation in the treatment of post-stroke dysphagia [J]. Chin J Clin Med, 2021,34(11):2196-2200.
235. Dongqiang Luo, Ying Shao, Feng Liu. Effect of external treatment of traditional Chinese medicine on patients with stable chronic obstructive pulmonary disease: a network Meta-analysis [J]. Journal of Advanced Nursing Education, 2021,37(2):151-158.
236. Han Na-Na, Ji Bo, Fang Yang, Zhao Guo-zhen, Liu Yi-tian, Wang Yi-fei, Wu Le, Guo Ting-ting. Efficacy and safety of multiple interventions in the treatment of mild to moderate Alzheimer's disease: a network Meta-analysis [J]. China Medicine Review, 2021,19(32):67-74+79.
237. Research progress of acupuncture and moxibustion in the treatment of decreased ovarian reserve function [J]. Clinical Research of Traditional Chinese Medicine, 2012,14(16):128-131.
238. Zixuan Wu, Minjie CAI, Peidong Huang, Jiayun Chen, Zhaohui Lv, Yuyan Huang. Meta-analysis of the correlation between depression and constitution of traditional Chinese medicine [J]. World Journal of Traditional Chinese Medicine, 2012,17(23):3366-3372.
239. Jingjing Li, Zhizhong Ruan, Cairong Zhang. Research progress of electroacupuncture in the treatment of chronic atrophic gastritis [J]. Massage and Rehabilitation Medicine, 2012,13(22):66-69.
240. Kong Zhen-Zhu, Zhou Tian-yu. Discussion on the treatment of loose diarrhea in Acupuncture and Moxibustion A and B Jing [J]. Chin J Basic Medicine of Traditional Chinese Medicine, 22,28(3):432-434+481.
241. Qingqing Guo, Gaiqin Yang, Weixun Qin Qin, Sa Zhang, Hui Sun, Yonggang Yu, Kaihuan Zeng. Research progress on the pathogenesis of functional constipation and acupuncture intervention [J]. Journal of Liaoning University of Traditional Chinese Medicine, 2021,24(11):203-206.
242. Gao Xiaoxia. Progress of traditional Chinese medicine treatment of essential tremor [J]. Massage and Rehabilitation Medicine, 2012,13(8):57-59.
243. Xue Si Hou, Fang Yuan, Jingqing Sun, Shaosong Wang, Xu Ji, Hongfang Tian, Cheng Tan. Based on the construction of clinical thinking of acupuncture and moxibustion, improving the medical record writing ability of standardized training doctors: the teaching experience of Acupuncture and Moxibustion, a textbook for national standardized training of TCM residents [J]. Chinese Acupuncture and Moxibustion, 2012,42(11):1306-1310.
244. Guo Ziyi, Nie Qiaofeng, Zhang Jicheng, Wang Lanlan, Yang Wanfang, Tan Shun 'e. Clinical effect of conventional acupuncture combined with traditional Chinese medicine fumigation on postherpetic neuralgia [J]. Jilin Med, 2021,43(11):3057-3060.
245. Du Zhongming, Fang Jiliang, Lu Mengxin, Jiang LAN, Zhao Jiping, Zou Yihuai. fMRI study on the variation coefficient of posterior degree centrality and movement attitude centrality of Yanglingquan in stroke patients with acupuncture [J]. Chin J Medical Physics, 2012,39(1):38-43.
246. Yuan Yi-yun, Zhang Li, Wu Chang-le, Zhang Ning, Lu Pei-ran, Xing Wenwen, Qiao Hai-fa, Yang Xiao-Hang, Liu Qi. Research progress of acupuncture and moxibustion regulating brain energy metabolism to prevent and treat neurodegenerative diseases [J]. Journal of Zhejiang Chinese Medical University, 222,46(9):1002-1007+1014.
247. Pei-wei Xian, Li-Mei Gao, Yuke Yang. Therapeutic effect and mechanism of electroacupuncture on Alzheimer's disease model mice [J]. Beijing Traditional Chinese Medicine, 2021,41(2):136-140.
248. Fanjie Xiong, Wei Zhao, Kai Song, Ailing Huang, Hong Zhang. Traditional Chinese medicine therapy and cholinesterase inhibitors in the treatment of Alzheimer's disease: a network Meta-analysis [J]. Journal of Chengdu University of Traditional Chinese Medicine, 2021,45(2):104-112.
249. Liu Cunzhi. Current situation and thinking on the reliability of clinical research evidence of acupuncture and moxibustion [J]. Journal of Beijing University of Chinese Medicine, 2021,45(10):1018-1023.
250. Hong Zhou, Chen Zhao, Haochen Tang. Application of removing phlegm, removing blood stasis and curing essence combined with acupuncture at Zheng's experience points in middle-aged and elderly patients with patellofemoral arthropathy [J]. Sichuan Traditional Chinese Medicine, 2012,40(5):155-159.
251. Zhang Jing, Cheng Lianshun. Clinical observation on the treatment of acute exacerbation of knee osteoarthritis pain with fire needle retention [J]. Chin J TCM Emergency Medicine, 2021,31(2):281-284.
252. Xue Yu Ma, Chao Ming Chen. Clinical research overview of acupuncture and moxibustion in the treatment of Alzheimer's disease [J]. Acta Medicae Sinica, 2021,20(3):9-11.
253. Li Bangwei, Zhou Chuanlong, Wang Chao, Liu Yingjun, Fang Jianqiao. Professor Fang Jianqiao's experience in the treatment of postoperative urinary retention with acupuncture and moxibustion [J]. Journal of Zhejiang Chinese Medical University, 222,46(8):835-838.
254. Application progress of electroacupuncture in the rehabilitation of dysphagia after stroke [J]. Wisdom Health, 2021,8(1):25-28.
255. Zhou Chuntong, Wang Ying, Liu Huanyi. Research progress of acupuncture and moxibustion in the treatment of coronary heart disease [J]. Journal of Practical Traditional Chinese Medicine Internal Medicine, 2021,36(6):70-72.
256. Xinyu Zhang, Yunqi Lu, Hanbing Shen, Jianhua Chen, Haiyin Zhao. Clinical observation of acupuncture combined with conventional therapy in the treatment of antipsychotic drug-related metabolic syndrome [J]. Shanghai Journal of Traditional Chinese Medicine, 2021,56(1):58-62.
257. Ye Guancheng, Zhu Zhiwei, Chen Jiaqi, Zhang Zehan, Miao Ruiheng, Li Ting. Chinese Journal of Acupuncture and Moxibustion, 2012,42(9):1059-1063.
258. Cui Hailing, Li Yanju, Chen Xingsheng, Wang Minjun, Li Liaoyuan, Hu Ling, Wu Zijian. Efficacy and safety of elongated needle in the treatment of lumbar disc herniation: a Meta-analysis [J]. Journal of Hainan Medical College, 2021,28(24):1904-1911+1916.
259. Liu Sixi, Zhong Yuxian, Ma Guanghao, Liu Feng, Chen Tingting, Yang Zhaoqiang, Fu Bensheng. Clinical efficacy of ultrasound-guided acupuncture combined with functional rehabilitation in the treatment of pes anserinus bursitis caused by exercise training [J]. Journal of Naval Medical University, 2012,43(9):1081-1085.
260. Shufen Yuan, Jing Ye, Liqin Wang, Hong Liu. Clinical efficacy and safety evaluation of cynanbosum herbal tea combined with acupuncture in the treatment of non-alcoholic fatty liver disease [J]. Chinese Modern Physicians, 2021,60(9):137-139+167.
261. Li Y J. Research progress of nourish blood and strengthen the liver acupuncture therapy in the treatment of patients with depression after coronary heart disease intervention [J]. Capital Food & Medicine, 2012,29(6):14-16.
262. Lin Jieping, Zheng Xuefeng. To review the clinical experience sharing of acupuncture and moxibustion in the treatment of insomnia [J]. Chin and Foreign Med, 2021,41(13):195-198.
263. Lei Banglin, Xie Zhuoyu, Yuan Fengguo. Research on the progress of acupuncture and moxibustion in the treatment of depression [J]. World Composite Medicine, 2012,8(8):192-194+198.
264. Xiaobin Xie, Yamin Chen, Wei Zhang. Research progress of acupuncture and moxibustion in the treatment of post-stroke depression in recent 5 years [J]. Clinical Research of Traditional Chinese Medicine, 2021,14(16):142-145.
265. Jingjing He, Weizhong Hua, Lu Gao, Chengqiang Zheng. Progress of the mechanism of electroacupuncture in promoting the repair of sciatic nerve injury [J]. Chinese Journal of Cell Biology, 2021,44(11):2183-2189.
266. Liu B X, Li J Q, You T Y, Ma J J, Wang L, Ma T M. Clinical observation of "body and spirit regulating" acupuncture combined with Danggui Yinzi in the treatment of chronic urticaria with blood deficiency and wind dryness [J]. Chin J Traditional Chinese Medicine, 2021,40(5):77-80.
267. Yu Liying, Zhou Hongfei. Clinical research progress of electroacupuncture in the treatment of sequelae of stroke [J]. Journal of Practical Traditional Chinese Medicine Internal Medicine, 2021,36(2):29-32.
268. Zhang Bihua, Zheng Ling, Shao Hui, and Yang Liping. Rapid health technology assessment of Xiyanping injection in the treatment of respiratory diseases in children [J]. Chinese Hospital Drug Evaluation and analysis, 2012,22(2):208-212.
269. Xing Qingchang, Wang Weiwei, Hu Wenhui. Efficacy of acupuncture combined with traditional Chinese medicine in the treatment of idiopathic subjective tinnitus [J]. Chinese Armed Police Medicine, 2021,33(11):938-940+944.
270. Li Ziyun, Gu Renjun, Yang Yan, Hu Jingqing. Correlation between homocysteine and coronary heart disease phlegm and blood stasis syndrome [J]. World Science and Technology: Traditional Chinese Medicine Modernization, 2012,24(8):3149-3158.
271. Jiamin Zhu, Zhongren Sun, Yang Cui, Hongna Yin. Clinical research progress of acupuncture at Baliao points in the treatment of urinary incontinence [J]. International Journal of Traditional Chinese Medicine and Chinese Materia Medica, 2021,44(7):825-829.
272. Liang Li, Shaoyuan Li, Peijing Rong. Research progress on the regulation effect of acupuncture meridians and brain [J]. World Traditional Chinese Medicine, 2012,17(2):261-264.
273. Liu Yang, Zhang Lanxi, Tian Yan-ge, Zhao Gao-yuan, Chen Kai, Li Jian-sheng. Effect of Bufei-Yishen formula combined with acupuncture on chronic obstructive pulmonary disease (COPD) in rats based on onset time and late effect [J]. Chin J Traditional Chinese Medicine, 222,37(10):5664-5670.
274. Yan S Z. Clinical observation of electroacupuncture at tender points combined with spinal nerve root stimulation in the treatment of lumbar disc herniation [J]. Chinese Medicine Guide, 2021,20(25):129-131+135.
275. Yanji Zhou, Changxin Liu, Jiajia Zhang, Sheng Chen, Zhiwen Weng, Ying Wan, Changhe Yu. Analysis of prevention and treatment of epidemic disease and novel coronavirus pneumonia based on acupuncture and moxibustion [J]. Western Traditional Chinese Medicine, 2021,35(8):6-10.
276. Zhao Zhong. Study on acupuncture and moxibustion for vascular dementia [J]. Medical Information, 2012,35(15):175-178.
277. Chenxi Xu, Wei Yuan, Xinyi Chen, Haifa Qiao, Xinyan Gao. Effect of electroacupuncture on mood and estrogen receptor α in mice with polycystic ovary syndrome induced by bisphenol A exposure [J]. Acupuncture Research, 222,47(5):377-385.
278. Zhenxing Chen, Hesong Wang, Lidan Zhang, Xinhua Chen. Research progress on the mechanism of acupuncture and moxibustion in the treatment of Alzheimer's disease [J]. International Journal of Geriatrics, 2021,43(6):738-741.
279. Liao Dongmei, Pang Fang, Zhou Min, Li Yi, Yang Yunhao, Guo Xiao, Tang Chenglin. Effect of electroacupuncture on cognitive impairment in mice with Alzheimer's disease based on TLR4/NF-κB/NLRP3 pathway [J]. Acupuncture Research, 222,47(7):565-572.
280. Junhua Wang, Ying Yuan, Meiling Zeng, Bin Zhou. Study on the value of Qingnao Zhuyu decoction combined with acupuncture in promoting the recovery of neurological deficit in the recovery period of stroke [J]. Chin J Traditional Chinese Medicine, 2012,40(8):236-239.
281. Zhang Haiyan, Xiao Hongbo, Chen Yafeng. Phase Ⅰ clinical observation of shoulder intra-articular injection combined with acupuncture at Dong's Qi point in the treatment of shoulder-hand syndrome after stroke [J]. Journal of Anhui University of Traditional Chinese Medicine, 2021,41(4):65-68.
282. Rao Qian, Tan Zhiwen. Effect of QufengHuatan Tongluo decoction combined with acupuncture and massage on upper limb muscle strength in patients with acute cerebral infarction and hemiplegia of wind-phlegm stasis type [J]. Sichuan Traditional Chinese Medicine, 2012,40(3):163-166.
283. Yang Meng, Zhou Peng. Discussion on the prevention and treatment effect of acupuncture and moxibustion on coronavirus disease 2019 (COVID-19) based on the concept of "preventive treatment for disease" in traditional Chinese medicine [J]. Clinical Journal of Acupuncture and Moxibustion, 2021,38(1):82-85.
284. Wang Ping, Chen Huaizhen, Li Luoyi, Yang Jun, Yang Wenming, Wang Pin. Acupuncture for cerebral dystonia of hepatolenticular degeneration with damp-heat accumulation: a randomized clinical trial [J]. World Journal of Acupuncture and Moxibustion: English edition, 22,32(3):193-198.
285. Wu Changle, Zhang Li, Yuan Yyun, Li Yongfeng, Wu Yuwei, Ha Lue, Yang Haiyong, Xing Wenwen, Qiao Haifa, Yang Xiaohang, Liu Qi. Effect of acupuncture and moxibustion on mitochondrial dynamics [J]. Chin J Traditional Chinese Medicine, 2021,37(4):2152-2156.
286. Qiu Li, Gong Dun, Zhao Jialiang, Yang Shengli, Ke Dongfen, Jiang Lei, Hu Na, Wei Rong. Clinical study of external diaphragm pacing combined with electroacupuncture in the treatment of chronic obstructive pulmonary disease [J]. Clinical Journal of Traditional Chinese Medicine, 2021,34(3):531-534.
287. Weina Xu, Shuangjia Chen, Shenfeng Gu, Yijia Chen. Clinical effect of Penyan decoction combined with acupuncture on sequelae of pelvic inflammatory disease of Qi stagnation and blood stasis type [J]. Journal of Shanghai University of Traditional Chinese Medicine, 2012,36(2):26-30.
288. Yuan Zhang, Qiarui Tan, Yu Wang, Ziling Qin, Hai Lu, Chunhong Zhang, Xiaofeng Zhao. Research progress on the brain effect of laser acupuncture in recent 20 years [J]. Journal of Molecular Imaging, 2021,45(1):146-150.
289. Jie Li, Ping Wang, Jing Le, Huiping Wei, Hui Zhao. Effect of acupuncture combined with modified Maxing-Shigan decoction on severe acute pancreatitis-related lung injury and NGAL and TLR4 in peripheral blood [J]. Chin J TCM Emergency Medicine, 2021,31(12):2132-2135+2143.
290. Manlu. Research overview of acupuncture and moxibustion in the treatment of primary dysmenorrhea from 2015 to 2019 [J]. Modern distance education of traditional Chinese Medicine in China, 2020,20(15):203-205.
291. Zhaoliang Li, Liqian Yan, Ruqi Zhang, Xuan Wang, Zhen Wang, Tingli Dong, Guizhi Zheng. Analysis of the characteristics and rules of acupuncture and moxibustion in irritable bowel syndrome based on data mining technology [J]. Journal of Jining Medical University, 2021,45(4):265-270.
292. Dean Zhang, Zhihong Li, Mei Wu. Clinical application status of internal heat acupuncture and exploration of traditional Chinese Medicine guidance Theory [J]. Chinese Recuperative Medicine, 2021,31(10):1061-1065.
293. Dan Li, Tengyu Chen, Yafen Huang, Min Zhou, Yixing Zhou, Yan Yan, Yajie Yan. Research on olfactory disorders in China: a visual analysis based on Citespace [J]. Journal of Otolaryngology and Ophthalmology, Shandong University, 2012,36(4):40-48.
294. Yin Di, Dong Min, Guo Fengjiao. Research progress of acupuncture and moxibustion in the treatment of postpartum urinary retention [J]. Basic Chinese Medicine, 2021,1(1):81-86.
295. Geng Yanxia, Jiang Hua, Lv Hai, Chen Dong, Chen Qiuhua, Zhou Haiqi, Pei Yinghao, Yuan Mengqian. Clinical observation of electroacupuncture intervention on ventilator-induced diaphragmatic dysfunction [J]. China Emergency Medicine of Traditional Chinese Medicine, 222,31(3):490-493.
296. Wang Xugang, Zhang Xiaoping, Yang Liping, Mo Yafeng, Sheng Shujie, Xuan Ziqi, Wang Lili. Wang Li-li's experience of treating refractory scapular periarthritis based on dynamic acupuncture [J]. Journal of Zhejiang Chinese Medical University, 222,46(1):69-73.
297. Xu Yingjie, Yao Xiaoqiang, Zheng Xianli, Li Dingpeng, Huo Linyu. Research progress on the mechanism of acupuncture and moxibustion in the intervention of rheumatoid arthritis based on NF-κB signaling pathway [J]. Journal of Massage and Rehabilitation Medicine, 2012,13(17):50-54+60.
298. Zhou Dan, Chen Lu, Wang Xuedong, Song Xiaoxiang, Wang Fuchun. Research progress on the mechanism of acupuncture and moxibustion in the prevention and treatment of gastric ulcer [J]. Journal of Changchun University of Traditional Chinese Medicine, 2021,38(5):573-576.
299. Jie Li Dai, Zhizhong Ruan. Research on the status of acupuncture and moxibustion therapy in the treatment of polycystic ovary syndrome [J]. Journal of Contemporary Medicine, 2012,20(1):30-32.
300. Yanan Qian, Dongsheng Qiu. Clinical research progress of acupuncture and moxibustion in the treatment of peripheral facial paralysis in recent five years [J]. Massage and Rehabilitation Medicine, 2012,13(7):48-50.
301. Wan Qing, Wei Qingzhong, Zou Chengsong, Ning Huijun, Shao Shuiyan, Chen Tao, Yi Hongchi. Effect of SuperPATH hip replacement combined with acupuncture and giant needling movement in the treatment of femoral neck fracture [J]. Journal of Yunnan University of Traditional Chinese Medicine, 2021,45(1):40-46.
302. Wan Qing, Ma Jin. Research progress of acupuncture and moxibustion in the treatment of post-stroke dysarthria [J]. Journal of Practical Traditional Chinese Medicine Internal Medicine, 2021,36(7):97-99.
303. Wang Chengxin, Chen Feng, Liao Jiabao, Chen Fengjuan. Effect of electroacupuncture combined with early probiotics on acute gastrointestinal injury secondary to severe neurological disease [J]. Chin J TCM Emergency Medicine, 22,31(11):1986-1990.
304. Wang Chaoan, Xu Junfeng, He Jun. Research status of clinical randomized controlled trials of placebo acupuncture [J]. Chinese Journal of Acupuncture and Moxibustion Electronic Journal, 2021,11(4):162-164.
305. Jie Tang, Ping Wu, Zeyun Yu, Yun Luo, Ciyu Tao, Yini Wang. Research progress on the effect of acupuncture and moxibustion on pain regulation neurotransmitters in the reward system in the treatment of chronic pain [J]. Chin J Traditional Chinese Medicine, 222,37(3):1570-1573.
306. Yaling Zheng, Dongling Zhong, Rongjiang Jin, Yijie Huang, and Juan Li. Overview of the research on the treatment of hypertension from Yangming based on the theory of "pulse distention" [J]. World Science and Technology: Traditional Chinese Medicine Modernization, 22,24(9):3631-3637.
307. Han Xu, Wang Mo, Li Yan. Clinical study on Tongdu Xingshen acupuncture in the treatment of cognitive impairment of cerebral small vessel disease [J]. Journal of Clinical Acupuncture and Moxibustion, 2021,38(2):25-28.
308. Wang Quanzhong, Tang Rui, Li Kun, Xiao Liqin, Wu Jun, Hu Wei, Tian Tian. Clinical study on the effect of Yangming meridian row acupuncture combined with electroacupuncture on the early rehabilitation of patients with cerebral infarction [J]. Chinese Medical Innovation, 2021,19(31):117-121.
309. Wang Jiange. Clinical research progress of acupuncture and moxibustion in the treatment of knee osteoarthritis [J]. Chinese Rehabilitation Medicine, 2021,31(11):1161-1164.
310. Xingguo Xie, Xu Dong, Hongyan Zhang, Xin Yin, Ying Zhang, Dongyan Wang. Effect of alternating Fu's Fu's acupuncture and electroacupuncture on upper limb dysfunction after stroke [J]. Chinese Journal of Traditional Chinese Medicine, 2012,50(10):60-65.
311. Xue Jiaxin, Cui Yanan, Gao Ziyin, Ma Xingwu, Wu Zhenqi, Xu Ke, CAI Guofeng. Research progress on the dominant diseases of crossing np-EA treatment [J]. Chinese Medicine, 2021,11(6):1175-1181.
312. Wen Xu, Wang Dongxin. Effect of Lingguizhugan Decoction combined with Wenyang Tongluo acupuncture on pulmonary function in patients with chronic obstructive pulmonary disease [J]. Chinese and Foreign Med, 2021,41(9):190-194.
313. Zhou Zhien, Lu Ping, Yao Juan, Yang Liang. Clinical study of Shenfu injection combined with electroacupuncture in the treatment of septic shock [J]. New Traditional Chinese Medicine, 2021,54(19):116-120.
314. Lin Yan, Zhuang Jie, Ding Yong. Research progress of external treatment of traditional Chinese medicine based on acupuncture and Tuina in the treatment of children with Toutic syndrome [J]. Collection of Contemporary Medicine and Medicine, 2021,20(1):147-150.
315. Chen Shangyun, Zhong Guilin, Song Peirong, Zheng Shijiang, Li Jianwei, Wang Fei, Liu Qiang. Research progress of acupoint catgut embedding therapy for allergic rhinitis [J]. Clinical Research of Traditional Chinese Medicine, 2012,14(18):59-61.
316. Guo Chunlei, Ma Yue, He Jiakai, Sun Jifei, Wang Zhi, Wang Lei, Zhang Jinling, Fang Jiliang. Experimental protocol of resting-state fMRI study on the brain mechanism of auricular concha electroacupuncture for mild cognitive impairment [J]. Chin J Imageology of Integrated Traditional Chinese and Western Medicine, 2021,20(1):21-24.
317. Tang Haochen, Hu Rui, Tang Liugang, Wang Biao, Cheng Yuandong, Kang Huimin. Kinesiogram combined with electroacupuncture for the treatment of type Ⅰ acromial impingment syndrome [J]. Chin J Orthopedics, 2012,35(10):957-962. (in Chinese)
318. Li Xiaolin, Wan Hongmian. Clinical application progress of Fengchi point in recent 10 years [J]. Journal of Liaoning University of Traditional Chinese Medicine, 2021,24(1):147-150.
319. Wang Chao, Li Yingchun, Zhu Junchen, Xiong Yingzong, Nie Yong, Ma Xingfu, Su Yi, Zheng Zhiwen. Clinical effect of "correcting tendon and setting bone method" in the treatment of knee osteoarthritis [J]. Journal of Anhui University of Traditional Chinese Medicine, 2021,41(5):80-84.
320. Qin Zhengwei, Wang Shun, Bai Yan, Li Yuan. Research progress of traditional Chinese medicine in the treatment of levodopa-induced dyskinesia [J]. Chinese Journal of Traditional Chinese Medicine, 2021,37(5):985-990.
321. Liu Haiyan, Yu Ze. Clinical research progress of acupuncture and moxibustion in the treatment of endometriosis infertility [J]. Massage and Rehabilitation Medicine, 2012,13(14):74-77.
322. Jia Kunping, Wu Jianli, Liu Guanping, CAI Guofeng, Liang Qun. Research progress of acupuncture and moxibustion in the treatment of sepsis based on cholinergic anti-inflammatory pathway [J]. Chin J Traditional Chinese Medicine, 2021,37(8):4277-4280.
323. Wen-ran Qiu, Zen-Min Xu, Wei Shen, Guo-Jing Fu, Liu-Ding Wang, Shao-Jiao Liu, Hongxi Liu, Yue Liu, Ye-Fei Wang, Ling-Ling Dai, Chun-Yan Guo, Weiwei Jiao, Xing Liao, Yu Liu, Xiao Liang, Yun-ling Zhang. Overview of the evaluation of the advantages of traditional Chinese medicine in the treatment of ischemic stroke [J]. Chinese Journal of Experimental Formula Science, 222,28(12):225-232.
324. Chen Jianlin. Clinical research on the treatment of shoulder pain in stroke patients with ultra-micro acupotome combined with easy cupping method [J]. New Traditional Chinese Medicine, 2012,54(18):128-132.
325. 4Xin Hu, Zhengying Luo, Peifang Zhao, Xin Lu, Liping Zhao, Hongbo Liu, Zhudi Wu, Yong Zhao, Jing Zhang, Shenglin Ren, Caiwen Wu, Jiayong Liu. Identification and evaluation of smut resistance of wild sugarcane species of scission and large stem [J]. China Sugar Crops, 2021,44(3):54-59.
326. Ning Gao, Yufeng Guo, Yue Meng, Xinqiao Chu, Peichu Zhang. Bibliometric study on the clinical application of the old ten-needle [J]. International Journal of Traditional Chinese Medicine and Chinese Materia Medica, 222,44(4):438-442.
327. Ying-jie Fan, Shu-min Huang, Xiao-ying Xie, Zhuo-Hua Chen, Yanbing Li, Bin Nie. Modern clinical application and research of Shuliao points [J]. Clinical Research of Traditional Chinese Medicine, 2012,14(36):106-109.
328. Bo Wang, Xin Xu, Nan Sui, Lide Zhang. Clinical discussion on the combined treatment of acupuncture and medicine based on "Kaixuanfu method" in Switzerland [J]. Liaoning Journal of Traditional Chinese Medicine, 222,49(11):44-47.
329. Application value of acupuncture combined with conventional western medicine in postoperative treatment of critically ill patients with cerebral hemorrhage [J]. Chin J Practical Med, 2021,49(3):120-122+F0003.
330. Li X M, Han L L, He Y N, Zhang X C, Chen W M. Evaluation of resistance of 20 apple varieties (types) to pear fire blister [J]. Plant Quarantine, 2012,36(4):6-12.
331. Yuchan Zhang, Yujun Wan, Juan Zhang, Yi Tan. Treatment of stress urinary incontinence and pelvic organ prolapse with transvaginal Er:YAG laser combined with electroacupuncture [J]. Chinese and Foreign Med Research, 2012,20(13):37-41.
332. Zhou G P. Treatment of neuromyelitis optica with acupuncture and medication: a case report and literature review [J]. Chinese Primary Medicine, 222,29(9):1383-1385.
333. Zhang Lingjia, Cheng Zedong. Clinical research progress of acupuncture combined with medicine in the treatment of atherosclerosis [J]. Journal of Practical Chinese Medicine Internal Medicine, 2021,36(6):95-97.
334. She Kai, Ma Danmei, He Jun. Clinical research progress of auricular acupoint therapy [J]. Chinese Journal of Acupuncture and Moxibustion Electronic Journal, 2021,11(3):111-112+122.
335. Liu Yue, Gao Shangshu, Yang Nan, Tan Yuanfei, Chu Yunjie. Clinical research progress of governor vessel moxibustion in the treatment of ankylosing spondylitis [J]. Journal of Changchun University of Traditional Chinese Medicine, 2021,38(1):105-108.
336. Ciyu Tao, Ping Wu, Jie Tang, Nannan Jiang, Xue Wang, Luoxian Shuai, Xinyue Hu. Research status and thinking of central regulatory network of chronic pain [J]. World Science and Technology: Traditional Chinese Medicine Modernization, 2012,24(1):258-264.
337. Zhang Xin-Pu, Zhong Hui, Zheng Xue-Na, Xie Yin-Rou, Sun Bing, Li Xiao-di, Zhou Cheng, Cheng Lina, Luo Yong, Lv Hui-sheng, Shen Wei, Li Xiao-Li, Xu You-Cheng, Chi Pei-Pei, Rao Li-bin, Chen Zhen-cheng, Ye Zhi-hui, Li Jia-min, Wang Lin. Conception and design of the research program on the mechanism of functional plasticity of electroacupuncture at giant Yangming meridian in the treatment of hemiplegia after ischemic stroke based on the central-peripheral-central closed-loop rehabilitation theory [J]. Liaoning Journal of Traditional Chinese Medicine, 222,49(7):165-169.
338. Liu Ying-zhe, Song Luyao, Xing Si-ning, CAO Yu-Fang, Pan Xiang-bin. Clinical research progress of traditional Chinese medicine in the treatment of Graves ophthalmopathy [J]. Chinese Journal of Traditional Chinese Medicine, 2012,50(6):112-115.
339. Menglong Zhang, Bifang Zhuo, Chenyang Qin, Bomo Sang, Zhihong Meng. Common evaluation methods of aphasia and their reliability and validity [J]. Jilin Traditional Chinese Medicine, 2012,42(3):366-369.
340. Tan, Dai Ming. Research progress of characteristic therapy of Guangxi Zhuang medicine in the treatment of insomnia [J]. Clinical Research of Traditional Chinese Medicine, 222,14(14):142-145.
341. Gu Tian, Yan Weiping, Ma Derui, Zhang Xinlong, Huang Chen. Progress of traditional Chinese medicine treatment for adolescent idiopathic scoliosis [J]. Clinical Research of Traditional Chinese Medicine, 2012,14(4):126-129.
342. Chen Xie, Yixin Zheng, Chaojun Fang, Yunfei Chen. Clinical study on the factorial design of acupoint application in the treatment of insomnia [J]. Clinical Journal of Acupuncture and Moxibustion, 2021,38(6):20-26.
343. Zhang Shicai, Li Yi-fei, Wang Chun-ping, Yang Xiao-miao, Huang Qi-zhong, Huang Ren-zhong. Identification and evaluation of resistance to Colletotrichum oxysporum in pepper germplasm [J]. Acta Hortologica Sinica, 2012,49(4):885-892.
344. Peng Suiying, Wang Xiaolan, Zhou Xiaoming, Feng Beibei, Wang Weiming, Liu Entong. Comprehensive rehabilitation training for complex dysfunction in a child with giant gyria: a case report and literature review [J]. Journal of Massage and Rehabilitation Medicine, 2012,13(20):68-72.
345. Ling Haiyan, Dai Yunxia. Application effect of blunt needle button-hole puncture technique in hemodialysis patients with arteriovenous fistula [J]. Chinese Primary Medicine, 2021,29(7):1039-1043.
346. Yao Chongjie, Kong Lingjun, Zhu Qingguang, Cheng Yanbin, Lu Zhizhen, Tang Cheng, Fang Min. Research progress of external treatment of traditional Chinese medicine in the treatment of non-alcoholic fatty liver [J]. Journal of Hainan Medical College, 2021,28(6):476-480.
347. Xu Aili, Li Yuan, Yang Jianqin, Su Xiaolan, Zhang Xiaohong, Liu Tao, Sun Xiaohong, Wei Wei. Research progress of Tianshu and Shangjuxu as basic acupoints in the treatment of gastrointestinal diseases [J]. World Journal of Traditional Chinese Medicine, 2021,17(21):3096-3100.
348. Wang Siqi, He Zhuojuan, Hua Yunzhen, Shan Exian, Tai Xiantao. Analysis of the current situation of three methods of external treatment of traditional Chinese medicine in the treatment of cognitive dysfunction in cerebral palsy [J]. Massage and Rehabilitation Medicine, 2012,13(6):71-73+77.
349. Chen Likun, Xu Yeting, Wang Yongpeng, He Linzi, Zeng Bin, Ai Shajiang · Mai Mai Ti. Evaluation of fire blister resistance of pear germplasm resources in Xinjiang [J]. China Fruit Trees,2022(8):16-22+F0003.
350. Liu Jun. Non-pharmacological treatment of gastroesophageal reflux disease in the elderly [J]. Chin J Clin Health Care, 2012,25(2):168-171.
351. Yong Li, Weifeng Wang. Establishment of evaluation system for gastrointestinal dysfunction after stroke [J]. Journal of Practical Traditional Chinese Medicine Internal Medicine, 2021,36(3):82-83.
352. Xiaotong Zuo, Qiaofeng Wu. Research progress on the relationship between intestinal diseases and circadian rhythm disorder [J]. Chinese Journal of Practical Medicine, 2021,38(18):2363-2366.
353. Sheng Nan, Tian Hui, Ma Tieming. Research progress on clinical application of giant needling technique [J]. Chinese Contemporary Medicine, 2012,29(6):20-23+31,F0004.
354. Jingyue Gao, Wei Liu, Aihua Wang, Peihao Li, Shumin Zhang. Research progress on the mechanism of pyroptosis in rheumatic immune diseases [J]. Chinese Journal of Clinical Health Care, 2021,25(4):562-567.
355. Yu-hai he, lily, lunch, Dai Lianqing Lu Chang 碒, high, xian Huang Xingxian, LanKai, Huang Zeng ping. Effect of electroacupuncture on postoperative urination function in patients with anorectal disease based on the concept of preventive treatment [J]. Modern Journal of Integrated Traditional Chinese and Western Medicine, 2021,31(13):1765-1768+1774.
356. Cheng Ling, Xiong Wei, Deng Changmao, Li Huannan. Progress in clinical application of acupotomology in orthopedic diseases [J]. China Med Sci, 2012,12(14):19-22+26.
357. Yan Xiangyun, Yao Junpeng, Zhang Wei, Yang Yuqing, Mu Linxuan, Chen Min, Li Ying.microRNA and functional bowel disease [J]. J Practical Med, 2021,38(2):243-248.
358. Wu Yu, Zhang Xin, Yuan Qing. Clinical research of scalp acupuncture in different schools for children with cerebral palsy [J]. International Journal of Traditional Chinese Medicine and Chinese Materia Medica, 2021,44(7):834-837.
359. Chen Yuzhu, Zhou Xingyu, Li Lei, Wang Jue. Research progress of acupoint autohemotherapy in the treatment of eczema in recent 10 years [J]. Clinical Research of Traditional Chinese Medicine, 2021,14(36):16-19.
360. Zheng Lifang, Sun Zhanling, Liu Chenghao, Zhang Jiamin, Jin Yabel. Professor Jin Yabei's clinical feature of selected acupuncture treatment of pregnancy disease based on the theory of "a cause without a victim" [J]. Journal of Zhejiang Chinese Medical University, 222,46(3):248-251+263.
361. Wang Yulin, Liu Tongyan, Sun Wei, Qu Yuanyuan, Feng Chuwen, Wang Zongxing, Gu Ye, Yang Tiansong. Current status and thinking on the evaluation methods of animal models of chronic fatigue syndrome [J]. Chinese Medicine Review, 2021,28(14):2884-2888.
362. Huang Yuxiu, Li Bushuang. Research progress of intradermal needling in the treatment of allergic rhinitis [J]. Clinical Research of Chinese Medicine, 2012,14(34):105-107.
363. Research progress of acupoint catgut embedding therapy for hyperlipidemia [J]. Clinical Research of Traditional Chinese Medicine, 2012,14(33):18-20.
364. Gao Simin, Wu Songbai. Research progress of traditional Chinese medicine treatment of chronic atrophic gastritis [J]. Clinical Research of Traditional Chinese Medicine, 2012,14(18):71-73.
365. Sun Kunkun, Han Xuechao, Sun Xiaofeng, Xu Wan-Li, Zhan Zhao-shuang, Wang Jia-feng. Research progress of traditional Chinese medicine in the prevention and treatment of atopic dermatitis [J]. Chinese Journal of Experimental Formulology, 222,28(5):266-273.
366. (in Chinese) Hua Ye, Zhang Liping. Application of traditional Chinese medicine nursing in neuroskin diseases [J]. Guangming Traditional Chinese Medicine, 2021,37(17):3217-3219.
367. Pang Jing, Yin Hongna, Sun Zhongren, Xia Kunpeng, Hong Jue. Clinical study on the treatment of senile Alzheimer's disease with acupuncture and Chinese medicine [J]. Acupuncture and Tuina Medicine: English edition, 2021,20(6):464-469.
368. Li Xia, Ouyang Xiali, Wang Xinxing, Liu Changsong, Mu Xiaohong, Tian Xiangdong, Yang Jizhou, Deng Bowen, Zhang Houjun, Zhou Liqun, Xu Lin, Zhao Jiping. Safety evaluation of Wudang medicine electric conduction therapy [J]. Chin J Traditional Chinese Medicine, 2021,37(10):5979-5983.
369. Yang Qunliu, Quan Jianfeng. Research progress of external treatment of traditional Chinese medicine in the treatment of malignant tumors and related diseases [J]. Jiangsu Traditional Chinese Medicine, 2012,54(2):77-81.
370. Guo Mengwen, Wu Pengfei, Xue Hongfeng. Current status of functional anorectal pain treatment: a literature review [J]. Chinese Journal of Painology, 2021,18(4):561-565.
371. Xue Ying, Wang Hao, Liu Yajie, Zhao Baixiao. Research progress of moxibustion in the treatment of endometriosis [J]. World Journal of Traditional Chinese Medicine, 2012,17(6):891-894.
372. Effect evaluation of different moxibustion modes on the intervention of chronic diseases in a small town with heat-sensitive moxibustion [J]. Jiangxi Traditional Chinese Medicine, 2021,53(11):39-43.
373. General situation and prospect of traditional Chinese medicine in the treatment of Alzheimer's disease [J]. Hainan Medicine, 2021,33(7):926-929.
374. Li Aijuan, Yang Juan, Xu Shuying. Effect of scraping with copper bianstone along meridians on relieving TCM clinical symptoms in female patients with cervical arthralgia [J]. Chinese and Foreign Women's Health Study,2022(7):25-28.
375. Jia Jing, Ma Chongbing, Wang Jue, Yan Xingke. Relationship between the nature of acupuncture sensation and the organization structure of acupoint area in doctors and patients [J]. Chin J Traditional Chinese Medicine, 222,37(10):5573-5576.
376. Liu Y F, Wang T F. Review of integrated traditional Chinese and western medicine in the treatment of perimenopausal syndrome [J]. Journal of Beijing University of Chinese Medicine, 2021,45(1):15-20.
377. Wang Dahai, Guo Hui. Advances in the diagnosis and treatment of primary biliary cholangitis with traditional Chinese and western medicine [J]. Frontiers in Medicine, 2012,12(21):39-42.
378. Panting, Wang Ying, Fan Dongying, Chen Yan. Evaluation of the effect of fire dragon therapy on patients with early knee osteoarthritis [J]. Western Chinese Medicine, 2012,35(6):118-120.
379. Tian Ziyu, Kong Lingbo, Zhang Chongyang, Pan Ting, Li Tingting, Liang Junjie, Li Yang, Hong Yuying, Liu Jingyi, Feng Zhiwei, Gao Ying, Liao Xing. A general review of clinical research evidence of oral Chinese patent medicine in the treatment of ischemic stroke [J]. Chinese Journal of Experimental Formulology, 2021,28(20):154-160.
380. Zhao Qi, Li Haisong, Wang Jisheng, Dai Hengheng, Zhang Huanan, Wang Bin, Zhang Xiaoxiao, Dang Jin. Clinical research review of traditional Chinese medicine in the treatment of benign prostatic hyperplasia [J]. Chinese Journal of Experimental Formulary Science, 222,28(2):236-241.
381. Lu Hong, Zhao Lingxiao, Yan Yongmei. Research progress of traditional Chinese medicine diagnosis and treatment of insomnia with anxiety and depression [J]. Journal of Liaoning University of Traditional Chinese Medicine, 222,24(6):101-105.
382. Wu Yongqiang, Feng Yue, Zhang Xiong, Zhao Yajun, Chen Dongnan, Ye Sillin. Research progress of Pushpi soil in the treatment of children's diseases [J]. Guide to Women and Children's Health, 2012,1(12):22-25.
383. Xu Guixing, Zhou Yumei, Sun Ning, Cui Jin, Chang Xiaorong, Ji Laixi, Liu Siyu, Luo Liaojun, Liu Xiaojia, Wang Dan, Zhao Ling, CAI Dingjun, Zheng Hui, Sun Mingsheng, Geng Guoyan, Cheng Jian, Liang Fan-rong. Chinese Journal of Acupuncture and Moxibustion, 2012,42(1):51-57.
384. He Wenqing, Li Jiansheng, Wang Minghang. Literature analysis of acupoint catgut embedding in the treatment of chronic obstructive pulmonary disease [J]. China Journal of Basic Medicine of Traditional Chinese Medicine, 2021,28(7):1140-1146.
385. Xue Qing, Cong Zhufeng, Xiang Zedong, Yu Xiaojun, He Mengyuan, Gao Peng, Dai Long, Gao Shuzhong. Review on the research of acupoint application preparations of traditional Chinese Medicine in the past ten years [J]. China Journal of Basic Medicine of Traditional Chinese Medicine, 2021,28(5):785-791.
386. Luo Lei, Wang Jialin, Ning Sisi, Wang Wanting, Zhong Yifei, Li Yi. Clinical observation on the treatment of chronic kidney disease stage 2-3b with depression with acupuncture and medicine [J]. Shanghai Journal of Traditional Chinese Medicine, 2021,56(12):61-67.
387. Xu Mei. A medical case of novel coronavirus pneumonia and literature review [J].TMR Classic Traditional Chinese Medicine Research, 222,5(2):23-28.
388. Jiang Piao, Su Leiyan, Zang Yuling, Zhang Lixia. Clinical application progress of external treatment of traditional Chinese medicine for prevention and control of juvenile myopia [J]. China Journal of Traditional Chinese Medicine Ophthalmol, 2021,32(4):325-328.
389. Zheng Xiaoting, Ma Ling, Li Junliang, Chen Shenghui, Yao Wenliang, Zhang Mingliang, Zhang Duanjun, Xiong Qi, Xiong Yong. Application progress of acupoint catgut embedding therapy in andrology [J]. Modern Diagnosis and Treatment, 2021,33(4):488-491.
390. Chin J TCM, 2021,63(7):601-607.
391. Gao Wen, Liang Fengming, Chen Tao. Advances in the genetics of myopia [J]. Int J Ophthalmol, 2021,22(7):1074-1078.
392. Qiu Shuwei, Liang Guanghe. Research progress of traditional Chinese medicine in the treatment of peptic ulcer [J]. Clinical Research of Traditional Chinese Medicine, 2012,14(21):108-112.
393. Xiong Guoxing, Zhang Guozhong, Wang Zemao, Sun Guorui, Li Yuna, Xue Qian. Clinical study on the treatment of knee osteoarthritis with acupotomery at Shenshu point [J]. Health Medical Research and Practice, 2012,19(11):74-78.
394. Zhang X P, Bai X H (Reviewing). Research progress on the mechanism of gastroesophageal reflux disease and its traditional Chinese medicine treatment based on autonomic nerve [J]. Chinese Journal of Integrated Traditional Chinese and Western Medicine Digestion, 222,30(7):531-535+540.
395. Zhai Yang. Research progress of traditional Chinese medicine in the treatment of ischemic stroke based on "brain-gut axis" [J]. Journal of Liaoning University of Traditional Chinese Medicine, 222,24(4):130-134.
396. Liang Lixin, Guo Yuhong. Research progress on the clinical application of the old ten needles in the treatment of digestive system diseases [J]. World Traditional Chinese Medicine, 2012,17(16):2370-2372+2377.
397. Gao Sen, Meng Xiaonan, Li Chunying, Sun Jie, Yu Haikuo. Phase Ⅰ clinical observation of Wang Juyi meridian diagnosis method combined with Bobath rehabilitation training in the treatment of shoulder-hand syndrome after stroke [J]. Chinese Acupuncture and Moxibustion, 2012,42(1):28-32.
398. Evaluation of the clinical application of the modified Minnesota Living with Heart Failure Questionnaire based on Chinese culture [J]. Journal of Integrated Traditional Chinese and Western Medicine Cardio-Cerebrovascular Disease, 2021,20(13):2309-2314.
399. Lu Zhimai, Huang Dandan, Xie Dingyi, Yue Ruizhen, Wang Jinwei, Luo Weifeng, Chen Rixin. Effect of moxibustion at "Deqi" on the expression of Aβ receptor-mediated transport and enzyme degradation related proteins in the hippocampus of Alzheimer's disease model rats [J]. Chinese Acupuncture and Moxibustion, 2012,42(8):899-906.
400. Wu Zhiwei, Zhang Shuaipan, Fang Min, Zhu Qingguang, Kong Lingjun, Cheng Yanbin. On the etiology and treatment of meridian tendon diseases [J]. Liaoning Journal of Traditional Chinese Medicine, 2012,49(1):53-55.
401. Zhang Yuan, Liu Yuan, Ren Qin, Zhang Qin, Li Li. Progress of traditional Chinese medicine treatment of allergic rhinitis in children [J]. Journal of Tianjin University of Traditional Chinese Medicine, 2021,41(1):131-136.
402. Ren Zhengkun, Wang Ying. Research progress of traditional Chinese medicine therapy in the treatment of coronary heart disease with depression [J]. Journal of Practical Chinese Medicine Internal Medicine, 2021,36(6):62-65.
403. Wei Yuting, Zhu Tiantian, Su Mingli, Jia Jing, Yan Xingke. Classification summary and preliminary evaluation of AD animal model prepared by D-galactose method [J]. Chin J Lab Zool, 2021,30(6):846-856.
404. Lai Xinxing, Tian Ziyu, Li Tingting, Qin Mingzhen, Cao Kegang, Gao Ying. Research progress on the "combination of disease and syndrome" of traditional Chinese medicine in the prevention and treatment of stroke [J]. Biomedicine Translation, 2012,3(3):22-30.
405. Zhang Nan, Cheng Yanmei, Wang Lei. Research progress on the mechanism of traditional Chinese medicine in the treatment of depression based on intestinal flora [J]. Chinese Journal of Experimental Formulary, 222,28(19):267-273.
406. Liu Shengwang, Chen Die ice, Lin yi, he feng. Mechanism of Prunella vulgaris on breast diseases [J]. Chinese Journal of Experimental Pharmaceutics, 2012,28(5):250-255.
407. Wu Sijia, Li Xiao. Research progress of traditional Chinese medicine in the treatment of ventricular premature contraction [J]. Clinical Research of Traditional Chinese Medicine, 222,14(33):28-31.
408. Research status of moxibustion therapy in the treatment of angina pectoris of coronary heart disease [J]. Tianjin Traditional Chinese Medicine, 2021,39(7):945-952.
409. Jiang Fei, Li Hongliang, Zhou Ran, CAI Haobing, Huang Qing, Yang Huan, Li Jing, Zhou Hao, Zeng Qiuming. Bilateral diffuse symmetrical white matter lesions in neuromyelitis optica spectrum disorders: a case report and literature review [J]. Chin J Neuroimmunology and Neurology, 2012,29(2):108-114.
410. Wang Yu, Bao Yehua, Huang Jinxiu, Yuan Jianhui, Wang Zhe, Lu Manman, Zhou Hongfei, Lin Xiaolin. Time-effect relationship and related factors analysis of muscle strength reconstruction in patients with acute ischemic stroke treated with eye acupuncture [J]. Liaoning Journal of Traditional Chinese Medicine, 222,49(11):183-187.
411. Wang Xuedong, Zhou Dan, Xu Ran, Chen Lu, Song Xiaoxiang, Wang Yukun. Application of biological ultra-weak luminescence in medical field [J]. Journal of Laser Biology, 2021,31(4):289-294.
412. Xue Xiaoyan, Chi Xiansu, Pan Jin, Wang Yong, Gao Zili, Lu Yanting, Pan Wenchao, Ma Ke, Ma Ting. Research progress of traditional Chinese medicine in the treatment of bipolar disorder [J]. World Science and Technology: Traditional Chinese Medicine Modernization, 2021,24(7):2817-2824.
413. Wang Hanlin, Chen Yu, Guo Xinfeng, Kang Fuqin, Chen Jiaxin, Chen Zhaoyue, Zhao Shunying, Yin Lihong, Wang Ji, Li Yingshuai, Luo Jincao, Cui Haiying, Li Kangli, Xie Changcai. Correlation between constitution and body composition in obese outpatients with endocrine acupuncture and moxibustion based on Internet weight management database [J]. Journal of Beijing University of Chinese Medicine, 2021,45(5):500-506.
414. Wang Shuguang, Guo Feng, Ding Shilei, et al. Longitudinal study on the change trajectory and predictive factors of medication adherence in patients with Parkinson's disease [J]. Journal of Qiqihar Medical College,2023,44(06):583-588.
415. Wang Wenjie. Current status and influencing factors of psychological resilience in patients with Parkinson's disease [J]. Evidence-based Nursing,2023,9(06):1124-1126.
416. Sun Kangming, Ji Hong, Song Feifei, et al. Research progress on the application of telemedicine in patients with Parkinson's disease [J]. Nursing Research,2023,37(05):860-864.
417. Mo Xianrong, Wang Yi, Wu Danrong et al. Research progress on the effect of dance therapy on motor dysfunction in patients with Parkinson's disease [J]. Shanghai Medicine,2023,44(05):7-10+18.
418. Yao Xiaoyan, Zhang Dongdong, Jin Qingsong, et al. The relationship between cognitive impairment and serum levels of Hcy, BDNF, ghrelin and obestatin in patients with Parkinson's disease and type 2 diabetes mellitus [J]. J Clin & Lab Med,2023,22(05):461-465.
419. Li Hanrui, Qin Liwei. The value of transcranial Doppler ultrasound in the differential diagnosis of vascular Parkinson's disease and primary Parkinson's disease [J]. Medical Information,2023,36(05):143-146.
420. Wang Qiahong, Zhang Xiaoqing, Guo Jing, et al. Research progress on influencing factors and intervention of home-based rehabilitation exercise in patients with Parkinson's disease [J]. Nursing Research,2023,37(03):471-477.
421. Yang Liming, Zhao Yang. Analysis of the relationship between olfactory dysfunction and smoking in Parkinson's disease [J]. Asia-pacific Traditional Medicine,2023,19(02):226-229.
422. Xu Xiaoyong, Yu Di, Li Jiaojiao. Design of walking AIDS for the elderly with Parkinson's disease based on ergonomics [J]. Design,2023,36(02):136-138.
423. Wei Yanli, Ji Yuan, Wang Chaofan, et al. Optimization design of Parkinson's walking aid based on QFD&TRIZ [J]. Packaging Engineering,2023,44(02):158-166.
424. Yao Kaifeng, Zhu Xiangyang, Li Jia, et al. The diagnostic value of serum MIF and LRRK2 expression combined with UPDRS score in Parkinson's disease and its relationship with disease stage [J]. J Clin & Lab Med,2023,22(02):120-123.
425. Zhang Yuemei, Wang Han. From the five zang-organs understanding olfactory disorder Parkinson's disease [J]. Journal of traditional Chinese medicine clinical journal, 2023, 35 (01) : 9-12. DOI: 10.16448 / j.carol carroll JTCM. 2023.0103.
426. Wang Wei, Cao Qinghua, Sun Guangling, et al. The differences of cognitive and motor functions in Parkinson's disease patients with cerebral microbleeds and their influencing factors [J]. Chin J Med,2023,18(01):38-42.
427. Yu Junwei. Clinical effect of selegiline combined with levodopa in the treatment of Parkinson's disease [J]. Clinical Research,2023,31(01):74-77.
428. Sun Huiqin, Li Chenxu, Bai Shengjie. Quantitative study of diffusion kurtosis imaging in Parkinson's disease patients with olfactory dysfunction [J]. Journal of Stroke and Neurological Diseases, 2021,39(12):1086-1090.
429. Shen Qi, Li Zhe, Su Qiaozhen, et al. Theory and practice of Chaihu plus Longgu Oyster decoction in the treatment of Parkinson's disease [J]. Chin J Clin New Med, 2012,15(12):1112-1116.
430. Liu Xiaoling, Wei Rui, Zhao Fengli. Changes of serum Lp-PLA2, S100β and sCD40L levels in patients with Parkinson's disease and their correlation with disease stage and cognitive impairment [J]. J Clin & Lab Med, 2021,21(23):2487-2491.
431. Huang Meng, Yan Yanna, Zhang Hui, et al. Clinical value of transcranial sonography in the diagnosis of Parkinson's disease [J]. Journal of Cardio-Cerebrovascular Disease of Integrated Traditional Chinese and Western Medicine, 2021,20(23):4376-4379.
432. Wang Yiying, Li Ruiqing, Li Jingwen, et al. Exosome-mediated cell communication: a potential biomarker analysis of Parkinson's disease [J]. Chinese Tissue Engineering Research,2023,27(24):3883-3891.
433. Li Lei. Clinical effect of benzhexol hydrochloride in the treatment of Parkinson's disease and Parkinsonism [J]. Chinese Journal of Wisdom and Health, 2021,8(33):94-98.
434. Guo Hailing, Chen Yiping, Wang Qiahong, et al. Current status and influencing factors of social participation in patients with Parkinson's disease [J]. Nursing Research, 2021,36(22):4018-4024.
435. Li Min, Li Yuhuan, Luo Jie, et al. The relationship between serum ESM-1, ET-1 expression and cognitive function in patients with Parkinson's disease [J]. Chin J Refractory Disease, 2012,21(11):1124-1128.
436. Zhang Yutong, Wang Qiuqin, Xu Yuchen, et al. A systematic review of the influencing factors of pain in patients with Parkinson's disease [J]. Chinese Journal of General Practice,2023,26(14):1766-1774.
437. Tang Wenjing, Luo Yongjie. Association between excessive daytime sleepiness and symptoms of Parkinson's disease [J]. Modern Clinical Medicine, 2021,48(06):412-415.
438. Peng Kai, Yan Wei-hong. Clinical research progress of Parkinson's disease with rapid eye movement sleep behavior disorder [J]. Journal of Chinese and Western Medicine Cardio-Cerebrovascular Disease, 2021,20(21):3934-3937.
439. Zhang Tingting, Lu Xiaobo, Liu Cailing. To investigate the relationship between ApoA-1, homocysteine, Cys-C and cognitive function in Parkinson's disease [J]. Systems Med, 2021,7(21):143-146+151.
440. Huang M Z, Liao X L, Huang Z J, et al. Research progress on the application of singing therapy in patients with Parkinson's disease [J]. Guangxi Medicine, 2021,44(19):2300-2303.
441. Zhao Zhihong, Wang Lijuan, Li Wenhui, et al. Research progress on the mechanism of curcumin in the treatment of Parkinson's disease [J]. Modern Medicine and Clinic, 2021,37(10):2390-2396.
442. Gong Siyuan, Liu Jihong, Li Jia, et al. High and low frequency repetitive transcranial magnetic stimulation improves motor and depressive symptoms in patients with Parkinson's disease: a Meta-analysis [J]. Evidence-based Nursing, 2012,8(19):2583-2589.
443. Sun Huiqin, Song Pu, Zhao Li, et al. Research progress of diffusion kurtosis imaging in Parkinson's disease [J]. Chinese Journal of Applied Neurology, 2021,25(10):1298-1303.
444. [39] Pang W Y, Zhu B, Li J, et al. Application and economic research of drugs for delaying the progression of Parkinson's disease in 30 tertiary hospitals from 2014 to 2019 [J]. Chinese Medicine Review, 2021,41(10):1513-1518.
445. He Yiting, CAI Canjia, Zeng Dongyu, et al. Study on the mechanism of Dading Fengzhu in the treatment of Parkinson's disease based on network pharmacology [J]. Journal of Shantou University Medical College, 2012,35(03):158-165.
446. Zhang Siyu. Correlation analysis of intestinal flora changes with clinical symptoms and therapeutic drugs in 70 patients with Parkinson's disease in a hospital [J]. Anti-infective Pharmacy, 222,19(09):1290-1293.
447. Zhu Jiaying, Xu Zhijing. Parkinson's disease recognition based on dynamic attention mechanism and multimodal cycle fusion [J]. Application Research of Computers,2023,40(02):481-487.
448. [J].Parkinson's disease and Parkinsonism [J]. Jiangsu Health Care,2022,No.297(09):51.
449. Chen Zhijun, Ma Jian, Tang Na, et al. Trend analysis and prediction of the disease burden of Parkinson's disease in China [J]. China Chronic Disease Prevention and Control, 2021,30(09):649-654.
450. Zhu Chunmu. Efficacy and adverse reactions of levodopa and benserazide combined with pramipexole in the treatment of Parkinson's disease [J]. Medical Information, 2021,35(18):112-114.
451. Yang Haohui, Liu Bin. Research progress of microglia in Parkinson's disease with depression model [J]. Hainan Medicine, 2021,33(17):2267-2270.
452. Zhang Chi, Yu Fan, Huang Xinxin, et al. Meta-analysis of risk factors for stroke complicated with aspiration pneumonia in Chinese population [J]. Chin J Gerontology, 2021,42(17):4137-4141.
453. Wang Qiahong, Qiao Caihong, Chen Yiping, et al. Research progress of Parkinson's disease patients' follow-up research [J]. Nursing Research, 2021,36(16):2949-2953.
454. Zhang Zhongwen, Chen Guohua, Huang Luqi, et al. Study on the protective effect of active ingredients of Huanglian-Jiedu Decoction on brain nerve cell damage in Parkinson's disease [J]. Chin J Comparative Med, 2012,32(10):17-23.
455. Baoxi, Bao Xiao-dong, HUANG Yun. Understanding of the relationship between Suwen · Arthralgia Theory and Parkinson's disease [J]. Zhejiang Journal of Traditional Chinese Medicine, 222,57(08):570-571.
456. Miao Weijie, Song Gang, Li Bing, et al. Exercise improves Parkinson's Disease: Research progress on the mechanism of intestinal flora [J]. Contemporary Sports Science and Technology, 2021,12(23):6-9.
457. Fan M D, Huang S, Wang Q, et al. Research progress of signaling pathways related to the intervention of traditional Chinese medicine on Parkinson's disease [J]. Chinese Journal of Traditional Chinese Medicine, 222,37(09):1869-1876.
458. Yu Li-chun, LIU Yuan-yuan, ZHAO Yue-qiu et al. Nursing Research, 2021,36(14):2572-2576.
459. Qin Liqing, Wu Lin, Pan Yibin, et al. Treatment of Parkinson's disease from spleen and stomach based on intestinal flora [J]. Journal of Liaoning University of Traditional Chinese Medicine, 2021,24(10):98-102.
460. CAI G L, Lin L C, Lin S J, et al. Clinical effect of Tiaoshen acupuncture combined with Bushen Huoxue Shugan decoction on Parkinson's disease with depression [J]. Chin J Traditional Chinese Medicine, 2012,40(12):248-251.
461. Zhang Xiaojun, Li Jue, Sun Hong. Humanistic thinking in the treatment of Parkinson's disease [J]. International Journal of Geriatrics, 2021,43(04):385-388.
462. Lin Pei. Research progress of traditional Chinese medicine in the treatment of depression in Parkinson's disease [J]. Popular Science and Technology, 2021,24(07):106-108+91.
463. Ren Z X, Zhang H M, Fu Z G, et al.miR-124-5p promotes the polarization of microglia to M2 type and reduces brain damage in Parkinson's disease rats by targeting STAT1 [J]. Advances in Anatomy, 2020,28(04):405-408+412..
464. Yu Jie, Li Qi, Zhao Feiyan. Gut microbiota as a new target for the treatment of Parkinson's disease [J]. Science and Technology of Food Industry, 2021,43(21):1-8.
465. Sun Yanan, Hao Yifeng, Li Xiaohong. Meta-analysis of the value of anal sphincter electromyography in the differential diagnosis of multiple system atrophy and Parkinson's disease [J]. Clinical Meta, 2021,37(06):490-496.
466. Liao Yang, Chen Jiao, Yang Dongdong, et al. Research progress on the relationship between ferroptosis and Parkinson's disease and the intervention mechanism of traditional Chinese medicine [J]. Guangxi Medicine, 2021,44(11):1276-1280.
467. Optimal target selection strategy for deep brain stimulation in Parkinson's disease based on simulated annealing particle swarm optimization [J]. Mathematical Modeling and its Application, 2012,11(02):49-60.
468. Improving the management of sleep disorders in Parkinson's disease in China [J]. Chin J Med Information Guide, 2012,37(11):8-8.
469. Peng Jiandong. Treatment of Parkinson's disease based on syndrome differentiation of viscera and meridians [J]. Shanghai Journal of Traditional Chinese Medicine, 2021,56(06):33-37.
470. Li Qin, Song Caiping, Chen Yulu, et al. Development and reliability and validity test of community nursing needs Scale for patients with middle and late Parkinson's disease [J]. Chin J Nursing, 2021,57(11):1351-1358.
471. Huang R O, Wang S, Sheng L, et al. Thoughts and methods of traditional Chinese medicine differentiation and treatment of depression in Parkinson's disease based on "liver dominating tendons and vessels, regulating emotions" [J]. World Science and Technology-Traditional Chinese Medicine Modernization, 2012,24(03):933-938.
472. Yang Peng, Mo Ying-min. Advances in genetic studies of sporadic Parkinsonism [J]. Chin J Applied Neuropathies, 2021,25(06):763-767.
473. Wang Meng, Li Yuan-Bo, Li Yi-fang, et al. Phospholipid remodeling and Parkinson's disease [J]. Acta Pharmacologica Sinica, 2021,57(06):1557-1564.
474. Qi Zhengqing, Xu Hong. Research progress on the application of remote monitoring in patients with Parkinson's disease [J]. Nursing Research, 2021,36(09):1607-1610.
475. Study on the best method of rotenone-induced Parkinson's disease model [J]. Journal of Integrated Traditional Chinese and Western Medicine Cardio-Cerebrovascular Disease, 2021,20(09):1611-1616.
476. Yao M F, Zhao Y. The basis of oral flora in traditional Chinese medicine tongue diagnosis and its correlation with Parkinson's disease [J]. Clinical Research of Traditional Chinese Medicine, 2012,14(13):89-92.
477. A new pathological study of mitochondrial lysosomal exocytosis in Parkinson's disease [J]. High Technology and Industrialization, 2020,28(04):66.
478. 1Yang Pan, Mao Lingyu, Liu Changqing, Liu Gaofeng. Research progress on the pathogenesis and treatment of Parkinson's disease [J]. Journal of Qiqihar Medical College, 2023,44 (05): 474-479
479. Bapir R, Bhatti KH, Eliwa A, García-Perdomo HA, Gherabi N, Hennessey D, Magri V, Mourmouris P, Ouattara A, Perletti G, Philipraj J, Stamatiou K, Trinchieri A,Buchholz N. Efficacy of overactive neurogenic bladder treatment: A systematicreview of randomized controlled trials. Arch Ital Urol Androl. 2022 Dec 28; 94(4):492-506.
480. Cordani C, Young VM, Arienti C, Lazzarini SG, Del Furia MJ, Negrini S, Kiekens C. Cognitive impairment, anxiety and depression: a map of Cochrane evidence relevant to rehabilitation for people with post COVID-19 condition. Eur J Phys Rehabil Med. 2022 Dec; 58(6):880-887.
481. Raoul S, Brissot R, Lefaucheur JP, Nguyen JM, Rouaud T, Meas Y, Huchet A,Razafimahefa N, Damier P, Nizard J, Nguyen JP. Additional Benefit of Intraoperative Electroacupuncture in Improving Tolerance of Deep Brain Stimulation Surgical Procedure in Parkinsonian Patients. J Clin Med. 2022 May 10; 11(10):2680.
482. Yang GY, Hunter J, Bu FL, Hao WL, Zhang H, Wayne PM, Liu JP. Determining the safety and effectiveness of Tai Chi: a critical overview of 210 systematic reviews of controlled clinical trials. Syst Rev. 2022 Dec 3; 11(1):260.
483. Zhang X, Wang S, Li X, Li X, Ran W, Liu C, Tian W, Yu X, Wu C, Li P, Li N,Wei Y, Wang Y, Yu S, Chen Z. Hemoglobin-binding α- synuclein levels in erythrocytes are elevated in patients with multiple system atrophy. Neurosci Lett. 2022 Oct 15; 789:136868.
484. Jang JH, Kim J, Kwon O, Jung SY, Lee HJ, Cho SY, Park JM, Ko CN, Park SU, Kim H. Effectiveness and Therapeutic Mechanism of Pharmacopuncture for Pain in Parkinson's Disease: A Study Protocol for a Pilot Pragmatic Randomized,Assessor-Blinded, Usual Care-Controlled, Three-Arm Parallel Trial. Int J Environ Res Public Health. 2023 Jan 18; 20(3):1776.
485. Carter AM, Dioso ER, Romero B, Clinker CE, Lucke-Wold B. Complementary Medicine and Expressive Arts Therapy: Adjuvant for Recovery Following Neurosurgical Procedures. OBM Integr Compliment Med.2023; 8(1):10.21926/obm.icm.2301007.
486. Cheng I, Sasegbon A, Hamdy S. Dysphagia treatments in Parkinson's disease: A systematic review and meta-analysis. Neurogastroenterol Motil. 2022 Dec 22:e14517.
487. Cao J, Chai-Zhang TC, McDonald CM, Kong J. Scalp Stimulation Targets for Neurological Conditions-Evidence from Large-Scale Meta-Analyses. J Integr Neurosci. 2022 Apr 15;21(3):83.
488. Raoul S, Brissot R, Lefaucheur JP, Nguyen JM, Rouaud T, Meas Y, Huchet A, Razafimahefa N, Damier P, Nizard J, Nguyen JP. Additional Benefit of Intraoperative Electroacupuncture in Improving Tolerance of Deep Brain Stimulation Surgical Procedure in Parkinsonian Patients. J Clin Med. 2022 May 10;11(10):2680.
489. Yang GY, Hunter J, Bu FL, Hao WL, Zhang H, Wayne PM, Liu JP. Determining the safety and effectiveness of Tai Chi: a critical overview of 210 systematic reviews of controlled clinical trials. Syst Rev. 2022 Dec 3;11(1):260.
490. Wu C, Guo H, Xu Y, Li L, Li X, Tang C, Chen D, Zhu M. The Comparative Efficacy of Non-ergot Dopamine Agonist and Potential Risk Factors for Motor Complications and Side Effects From NEDA Use in Early Parkinson's Disease: Evidence From Clinical Trials. Front Aging Neurosci. 2022 Apr 22;14:831884.
491. Bapir R, Bhatti KH, Eliwa A, García-Perdomo HA, Gherabi N, Hennessey D, Magri V, Mourmouris P, Ouattara A, Perletti G, Philipraj J, Stamatiou K, Trinchieri A, Buchholz N. Efficacy of overactive neurogenic bladder treatment: A systematic review of randomized controlled trials. Arch Ital Urol Androl. 2022 Dec 28;94(4):492-506.

**Exclusion criteria (13 articles)**

[1] The effect of non-pharmacological intervention on Parkinson's disease: a network meta-analysis [D]. China University of GeoSciences (Beijing),2021.

[2] LI M Y. Clinical research and mechanism exploration of acupuncture and moxibustion for Parkinson's disease myotonia based on "Cong Yang Lun zhi" [D]. Guangzhou University of Chinese Medicine,2020.

[3] JI X Y. Clinical efficacy of non-drug therapy in the treatment of Parkinson's disease [D]. Beijing University of Chinese Medicine,2018.

[4]Lee SH, van den Noort M, Bosch P, Lim S. Sex differences in acupuncture effectiveness in animal models of Parkinson's disease: a systematic review. BMC Complement AlternMed. 2016 Nov 3;16(1):430.

[5]Yang H J , Gao Y , Yun J Y , et al. Acupuncture does not protect against 1-methyl-4-phenyl-1,2,3,6-tetrahydropyridine-induced damage of dopaminergic neurons in a preclinical mouse model of Parkinson's disease[J]. Neuroreport, 2017, 28(1):50-55.

[6]Ko JH, Lee H, Kim SN, Park HJ. Does Acupuncture Protect Dopamine Neurons in Parkinson's Disease Rodent Model?: A Systematic Review and Meta-Analysis. Front Aging Neurosci. 2019 May 8;11:102.

[7] Wang Y Q. Acupuncture and moxibustion therapy for Parkinson's disease: a network Meta-analysis [D]. Xinjiang Medical University,2020.

[8]Gai C, Qiang T, Zhang Y, Chai Y, Feng W, Sun H. Electroacupuncture in treatment ofParkinson disease: A protocol for meta-analysis and systematic review. Medicine (Baltimore). 2021 Jan 22;100(3):e23010.

[9]Kim JI, Choi TY, Jun JH, Kang H, Lee MS. Acupuncture for management of lower urinary tract symptoms in Parkinson's disease: A protocol for the systematic review of randomized controlled trials. Medicine (Baltimore). 2018 Feb;97(6):e9821.

[10]Xu W, OuYang S, Chi Z, Wang Z, Zhu D, Chen R, Zhong G, Zhang F, Zhou G, Duan S, Jiao L. Effectiveness and safety of electroacupuncture in treating Parkinson disease: A protocol for systematic review and meta-analyses. Medicine (Baltimore). 2021 Mar 12;100(10):e25095.

[11]Zhi Y, Gao C. Acupuncture in the treatment of fatigue in Parkinson's disease: A protocol for systematic review and meta-analysis. Medicine (Baltimore). 2020 Nov 25;99(48):e23389.

[12] Buxiangjun, Gao Zhiqiang. Effect of acupuncture and moxibustion on Parkinson's disease: a systematic review [J]. Abstract of World Update Medical Information,2016,16(96):38-39.

[13]Fang Y, Xu Y, Liu Z, Dong S, Su Y. Efficacy and safety of abdominal acupuncture in Parkinson's disease: A protocol for systematic review and meta-analysis. Medicine (Baltimore). 2022 Nov 25;101(47):e31804.

**Did not meet the inclusion criteria (5 articles)**

[1] Xu Wei, Xiong Jun, Chen Rixin, Ouyang Sha, Yang Yanan, Liu Zuqin. Quality evaluation of randomized controlled trials of acupuncture and moxibustion for Parkinson's disease [J]. Chin J Traditional Chinese Medicine,2017,35(03):562-565.

[2] Huang Zhijun, Xiong Jun. Quality evaluation of clinical randomized controlled trials of acupuncture and moxibustion in the treatment of Parkinson's disease [J]. Journal of Shaanxi University of Traditional Chinese Medicine,2017,40(05):89-92.

[3]Cho KH, Kim TH, Jung WS, Moon SK, Ko CN, Cho SY, Jeon CY, Choi TY, Lee MS, Lee SH, Chung EK, Kwon S. Pharmacoacupuncture for Idiopathic Parkinson's Disease: A Systematic Review of Randomized Controlled Trials. Evid Based Complement Alternat Med. 2018 Jun 25;2018:3671542.

[4]Huang G , Guo M . Meta-analysis of the efficacy of acupuncture and moxibustion in the treatment of non-motor symptoms of Parkinson's disease[J]. Neural Computing and Applications:1-13.

[5]Chen W , Lian X . Systematic evaluation of traditional Chinese medicine for treating Parkinson's disease[J]. Chinese Journal of Neural Regeneration, 2010, 5(8):9.
