# Supplementary material for: Research methods and efficacy of acupuncture in the treatment of Parkinson's disease: a scoping review of systematic reviews and meta-analyses
Source: Front Neurol. 2023 Jun 2;14:1196446. doi: 10.3389/fneur.2023.1196446 (PMC10272821; doi:10.3389/fneur.2023.1196446)
Supplement: Supplementary file 3 [file Data_Sheet_3.docx]

| **Supplementary Material 3:Basic characteristics of included documents** | | | | | | | |
| --- | --- | --- | --- | --- | --- | --- | --- |
| **Included study** | **Sudy type** | **Therapy group** | **Control group** | **Number of Studies (Patients)** | **Methodology Evaluation Tools** | **Outcomes** | **Main conclusion** |
| Li2020 | System  evaluation | Acupuncture  Electroacupuncture  Acupuncture+western medicine  Electroacupuncture+western medicine  Abdominal acupuncture+western medicine | Western  medicine | 922 | Tool for risk of bias assessment | Webster scale  UPDRS scale  The curative effect | Acupuncture has a significant positive effect on the clinical effect of Parkinson's disease, but the future research of Parkinson's disease should be explored according to a more rigorous method. |
| Sun2013 | Meta  analysis | Acupuncture  Acupuncture+western medicine  Acupuncture+Chinese medicine  Acupuncture+Chinese medicine+Western medicine | Western  medicine  Fake stitch  Coupon  ning | 1325 | The Cochrane  partial  Risk assessment  tool | HAMD and SDS  UPDRS Ⅰ score  Webster volume  table  UPDRS Ⅱ  evaluation  The curative effect | Acupuncture and moxibustion therapy has a certain effect on some non-motor symptoms of PD, but it still needs to be further confirmed. |
| Yin2016 | Meta  analysis | Acupuncture  Acupuncture+ medication  Electroacupuncture+medication | medicine | 665 | The Cochrane  partial  Tool for risk of bias assessment | The curative effect | Acupuncture is effective in the treatment of Parkinson's disease, but there are methodological problems in the included randomized controlled trials. It is recommended to carry out rigorous and high-quality randomized controlled trials to further verify the curative effect. |
| zhou2020 | Meta  analysis | Acupuncture  Acupuncture+western  medicine  Electroacupuncture+western medicine | Western  medicine  Sham  acupuncture+western medicine | 632 | RCT bias risk assessment tool  recommended by Cochrane  Handbook 5.1.0 | UPDRS score Ⅲ  UPDRS Ⅲ  difference  UPDRSⅢ motor  symptom score  Adverse reactions | Acupuncture treatment can effectively improve the primary motor symptoms of Parkinson's disease, and the effect of acupuncture combined with western medicine is better than that of western medicine alone, but the above results need to be verified. |
| Yang2010 | System evaluation | Acupuncture  Electroacupuncture  Acupuncture+western  medicine  Electroacupuncture+western medicine  Abdominal acupuncture+western medicine | Western medicine | 832 | Cochrane  Reviewers'  Handbook 4.2.8 combined with  Jadad  recommended  quality a  ssessment criteria | To be efficient  Webster ratings  UPDRS score  Adverse reactions | Acupuncture and moxibustion is safe and effective in the treatment of Parkinson's disease. Acupuncture combined with western medicine may be superior to western medicine alone. However, these conclusions still need to be further verified. |

| Liu2019 | Meta  analysis | Electroacupuncture  Electroacupuncture+western medicine  Scalp acupuncture+western medicine  Scalp acupuncture+eye  acupuncture+conventional  western medicine treatment | Western  medicine  Conventional  treatment of western medicine | 864 | The Cochrane  partial  Tool for risk of bias assessment | Webster Scale  integral  Total UPDRS score  PDSS scale score | Scalp acupuncture (or combined with western medication) is superior to western medication alone in the improvement of motor symptoms and activities of daily living in PD patients, but there is no significant difference in the improvement of sleep between the two groups, which still needs to be further confirmed. |
| --- | --- | --- | --- | --- | --- | --- | --- |
| sui2016 | Systematic review  Meta  analysis | Acupuncture+western  medicine | Western  medicine | 590 | Risk of bias  assessment  criteria  developed by the Cochrane  Collaboration | The curative effect  UPDRS scores  Adverse reactions  Results follow-up | The efficacy, safety and side effects of ordinary acupuncture combined with madopar in the treatment of Parkinson's disease need to be further studied. |
| Liu2018 | Meta  analysis | Acupuncture  Acupuncture+western  medicine  Electroacupuncture+western medicine | Western  medicine | 892 | Risk of bias  assessment tool  provided by the Cochrane  Handbook for  Systematic  Reviews  (version 51.0) | Total effective rate  Webster scale  UPDRS score  Improvement IN  SYMPTOMS | Acupuncture is effective in the treatment of Parkinson's disease, which can improve the clinical symptoms of Parkinson's disease, delay the progression of the disease and improve the quality of life of patients to a certain extent. It is recommended to be widely used in clinical practice. |
| OuYang2017 | System  Evaluation | Acupuncture  Electroacupuncture  Acupuncture+medication  Electroacupuncture+medication  Coarse penetrating needle+medicine | medication | 1397 | Jadad scale | To be efficient  UPDRS score  Webster ratings  Adverse reactions | Acupuncture may be an effective and safe treatment for PD  treatment |
| Wen2021 | Meta  analysis  Qualitative review | Acupuncture+bee sting  (BV)  Acupuncture+Chinese  medicine  Acupuncture+western  medicine  Acupuncture+warm  acupuncture+Chinese  medicine  Head electroacupuncture+warm acupuncture+Chinese medicine  Acupuncture+Chinese medicine+Western medicine  Scalp acupuncture+traditional Chinese medicine  Electroacupuncture+Chinese medicine  Acupuncture  Acupuncture+Chinese medicine+triple bacteria  Electroacupuncture  Electroacupuncture plus western medicine  Acupuncture+electroacupuncture+Western medicineScalp electroacupuncture+Chinese medicine+Western medicine+cognitive training | Traditional Chinese medicine (TCM)  Waiting for the group  Western medicine  Sham acupuncture 1  Sham acupuncture+Chinese medicine  Chinese medicine+triple bacteria  Including false  Chinese medicine+Western medicine Chinese medicine+Western medicine +cognitive training | 4035 | Cochrane Risk of Bias assessment (version 5.1.0) | UPDRS scale  UPDRS Ⅱ  evaluation  UPDRS Ⅲ  evaluation  UPDRS Ⅰ score  UPDRSIV score  A 39-item  Parkinson's disease questionnaire  survey  Hamilton  Depression Scale  Minor mental  status examination | Acupuncture-related therapy combined with traditional Chinese medicine is safe and feasible. But given the limitations, the results should be interpreted with caution. In the future, large randomized trials with rigorous methodological quality and long-term follow-up are warranted. |
| Kwon2021 | Network Meta-  analysis | Electroacupuncture +conventional medication treatment  Hand acupuncture+conventional medication treatment  Bee venom needle+conventional medicine treatment | Conventional drugherap alone Sham acupuncture+conventional drug treatment | 1071 | Cochrane Risk of bias tool | UPDRS-III  UPDRS-II | Together with conventional levodopa therapy, bee venom acupuncture, electroacupuncture, and hand acupuncture may be more effective than single drug therapy in clinical practice |
| Lam2008 | System  evaluation | Acupuncture+western  medicine  Acupuncture +PD drug  treatment  Acupuncture | Western medicine  Sham acupuncture +PD drug treatmen No treatment | 580 | Not reported | webster Symptom Score  UPDRS | Acupuncture may be beneficial for patients with IPD, but there is insufficient evidence to support this conclusion due to methodological shortcomings of the study |
| Lee2008 | System  evaluation | electroacupuncture  Acupuncture  Scalp electroacupuncture + acupuncture  Abdominal needle | Sham  acupuncture  Western  medicine  No treatment | 484 | Modified Jadad  scale | UPDRS  Total effective rate  Webster scale | The evidence for the effectiveness of acupuncture for PD is not convincing, and further rigorous randomized controlled trials are needed. |
| Lee2013 | System  evaluation | Scalp electroacupuncture+ western medicine  Scalp acupuncture+western medicine | Western  medicine | 184 | Cochrane risk of bias | UPDRS  Webster Symptom Score | Acupuncture as a possible supportive or alternative treatment for PD, but the intensity of PD is not sufficiently determined. It is recommended to conduct high-quality, sham-controlled RCTS or comparative effectiveness studies with sufficient statistical sample sizes |
| Lee2017 | Systematic review  Meta  analysis | Acupuncture  Electroacupuncture  Electroacupuncture+medication  Acupuncture+medication | Sham  acupuncture  No treatment  medication | 1616 | Physical  treatment  evidence database (pedroscale)  The "rane" risk  scale (robc)  evaluation | Total effective rate  UPDRS  Webster scale | Acupuncture has a significant positive effect on relieving PD symptoms. For PD patients, acupuncture can be combined with conventional treatment. |
| Liu2017 | Systematic review  Meta  analysis | Acupuncture+medicine | medicine | 831 | Modified Jadad  scale | Clinical curative  effect  UPDRS  Adverse reactions | Compared with madopar alone, acupuncture combined with madopar can improve the clinical efficacy and safety in the treatment of Parkinson's disease. But this conclusion should be considered with caution. In the future, the effect of acupuncture combined with madopar on Parkinson's disease needs to be further clarified. |
| Noh2017 | Systematic review  Meta  analysis | Acupuncture  Electroacupuncture+acupuncture  Acupuncture+medication  electroacupuncture  Electroacupuncture plus medication  Medication+electroacupuncture+acupuncture | No treatment Drug  Acupoint  injection  Sham acupuncture  Including  false  Medication+  sham EA | 2625 | The Cochrane  Risk of Bias  Tool | UPDRS  Webster scale  Clinical effect | Acupuncture may be a safe and effective adjuvant treatment for PD patients. Future well-designed large-scale studies are needed to draw robust conclusions. |
| Qiang2019 | Systematic review  Meta  analysis | Medication + scalp electroacupuncture | Medication | 474 | The Cochrane  Risk of Bias Tool | UPDRS  Webster scale  To be efficient  Adverse reactions | SEA combined with drug therapy is effective in the treatment of PD, and the improvement of motor function in the SEA adjuvant therapy group is better than that in the drug therapy group, but the results should be viewed with caution. |
| Sun2023 | Meta  analysis | Acupuncture + western medicine  Acupuncture + Chinese medicine  Acupuncture plus repetitive transcranial magnetic stimulation  Acupuncture + Chinese medicine + Western medicine  Electroacupuncture + western medicine  Electroacupuncture + cognitive function training + western medicine  Scalp acupuncture + western medicine  Auricular acupuncture + traditional Chinese medicine  Acupuncture + repetitive transcranial magnetic stimulation + western medicine | Western medicine  Traditional Chinese medicine  Chinese medicine + Western medicine  Transcranial magnetic stimulation was repeated  Repetitive transcranial magnetic stimulation plus western medicine  Cognitive Training  Cognitive function training + Western medicine  Placebo acupuncture plus western medicine | 2349 | ROB Scale  Modified JADAD scale | Total effective rate  HAMD  PDSS  MMSE  MOCA  PSQI | The efficacy of acupuncture combined with other therapies in the treatment of Parkinson's disease with non-motor symptoms such as depression, cognitive impairment, sleep disorders and constipation is better than that of other therapies alone. The overall quality of this study is low, and more high-quality randomized controlled trials are needed to verify it. |
| Fu2022 | Meta  analysis | Acupuncture;Electroacupuncture + western medicine;Scalp acupuncture + western medicine ;Acupuncture + western medicine;Acupuncture + Chinese medicine;Warm acupuncture and western medicine | Sham acupuncture  Western medicine  Traditional Chinese medicine | 938 | Cochrane Handbook for Systematic Reviews (Version 5.1.0) | Total clinical effective rate  PDSS score  PSQI score  UPDRS score  PDQ-39 score  Adverse reactions | Acupuncture and moxibustion can significantly improve the sleep quality, PD-related symptoms and quality of life of PD patients, without obvious adverse reactions. |
| Pereira2022 | Systematic Review | Acupuncture + medicine  electroacupuncture  Acupuncture  Electroacupuncture plus medication  Acupuncture + bee venom needle  Acupuncture + Qigong meditation  Auricular acupuncture plus conventional treatment | Drugs  Sham acupuncture  No treatment  Qigong meditation | 845 |  | UPDRS  PDQ  Effective rate | Although all the literature pointed out that acupuncture has a positive effect on improving motor and non-motor symptoms of PD, it is difficult to make comparisons due to large differences in study design and methodology. |
| Li2022 | Systematic Review  Meta  analysis | electroacupuncture  Acupuncture  Electroacupuncture plus medication  Acupuncture + medicine | Drugs  Sham acupuncture  No treatment  Sham acupuncture plus medication | Not reported | Revised Cochrane Risk of Bias Tool (RoB 2) | PSQI  PDSS  HAMD  HADS  MoCA  UPDRS I and II | Acupuncture treatment can improve depressive symptoms, quality of life, cognition, general mental state, behavioral emotion and activity of daily living in PD patients. More prospective, large sample size, well-designed RCTS are still needed to confirm our findings. |
| Wei2022 | Meta  analysis | Acupuncture  Acupuncture + medicine  Electroacupuncture plus medication | Drugs  Sham-controls | 1008 | The Cochrane Collaboration | UPDRS-III  Parameters of gait  PDQ-39  MOCA | Acupuncture can improve motor function, gait disorder, cognitive function and quality of life in PD patients. However, in the present study, there were no significant changes between acupuncture monotherapy and sham controls or standard Parkinson medications. |
| UPDRS,Unified Parkinson’s Disease Rating Scale scores;HAMD，Hamilton Depression Scale；SDS,Self-rating Depression Scale;PDSS，Parkinson zs Disease Sleep Scale;MMSE,Mini-mental State Examination;MOCA,Montreal Cognitive Assessment;PSQI,Pittsburgh Sleep Quality Index; PDQ-39,Parkinsons’s disease questionnaire-39;HADS,Hospital Anxiety and Depression Scale | | | | | | | |
